# COACH Study: COVID-19 Influence on Cardiorespiratory Fitness in Athletes—A Systematic Review and Meta-Analysis

**DOI:** 10.3390/jcm15135133

**Published:** 2026-07-01

**Authors:** Przemysław Kasiak, Grzegorz Procyk

**Affiliations:** 1 3rd Department of Internal Medicine and Cardiology, Medical University of Warsaw, Żwirki i Wigury 61 Street, 02-091 Warsaw, Poland; 2 First Department of Cardiology, Medical University of Warsaw, Banacha 1A Street, 02-097 Warsaw, Poland; grzegorz.procyk@wum.edu.pl

**Keywords:** cardiorespiratory fitness, athletes, sports cardiology, COVID-19, peak oxygen uptake, endurance capacity, SARS-CoV-2

## Abstract

**Objectives**: We aimed to systematically review and meta-analyze the impact of COVID-19 infection on cardiorespiratory fitness (CRF): (1) within-athlete (the same participants before and after infection), and (2) between-athlete (infected vs. healthy reference participants). **Methods**: In this systematic review (PROSPERO Registry: CRD42024540430) we included observational studies enrolling recreational or competitive athletes ≥18 years old with laboratory confirmation of SARS-CoV-2 infection. The primary outcome was change in relative maximal oxygen uptake (VO_2max_). Secondary outcomes included changes in absolute VO_2max_, maximal ventilation (VE_max_), and maximal heart rate (HR_max_). We searched Embase, PubMed, Medline, Scopus, and Web of Science up to August 9th, 2025. Risk of bias was assessed with the JBI critical appraisal tool. Meta-analyses were performed with a random-effects model. **Results**: Twelve studies enrolling a total of 1595 participants met the eligibility criteria. COVID-19 infection was associated with lower relative VO_2max_ (MD = −1.83 mL·kg^−1^·min^−1^; 95%CI [−3.16, −0.49]; *p* = 0.007; I^2^ = 54%) and absolute VO_2max_ (MD = −0.15 L·min^−1^; 95%CI [−0.29, −0.01]; *p* = 0.03; I^2^ = 0%). COVID-19 infection was associated with lower VE_max_ (MD = −7.99 L·min^−1^; 95%CI [−12.94, −3.04]; *p* = 0.002; I^2^ = 0%) but not with HR_max_ (MD = −0.34 bpm; 95%CI [−1.54, 0.86]; *p* = 0.58; I^2^ = 0%). High heterogeneity of included studies was addressed with subgroup analyses. The risk of bias in most studies was high. The certainty of evidence was very low for each outcome. **Conclusions**: COVID-19 infection in athletes was associated with reduced VO_2max_ and VE_max_. The relationships were highly dependent on the quality of the studies. CRF and athlete profile should be considered when making shared decisions regarding safe return to sport after infection.

## 1. Introduction

Coronavirus disease 2019 (COVID-19) posed a challenge for the athletic community, with public restrictions leading to the suspension of sports competitions and the disturbance of regular training practice [1]. Despite athletes’ infections usually having a mild course, they still aggravate performance and COVID-19-associated sequelae occur [2,3]. About 1.5% of athletes could suffer from persistent symptoms lasting >3 weeks, and ~0.1% could have prolonged symptoms for >12 weeks. It should be underscored that exertional cardiopulmonary symptoms are more frequent, although still rare, and typically observed in 4.0% of athletes. Cardiovascular complications might be diagnosed in 1 of 11 athletes (~8.8%), and of athletes with chest pain who underwent cardiac magnetic resonance imaging during recovery from infection, one in five (~20.8%) had cardiac involvement (probable or confirmed) [3].

Cardiorespiratory fitness (CRF) depends on an individual’s endurance capacity and health state [2,4]. Maximal oxygen uptake (VO_2max_) is a gold-standard measure of cardiorespiratory fitness (CRF) [5,6]. In sports diagnostics, VO_2max_ is a strong predictor of athletic performance [5,7]. In clinical settings, VO_2max_ can be used to stratify risk and evaluate cardiovascular and respiratory system impairments [5,6]. Maximal heart rate (HR_max_) is also one of the most important variables used in clinical medicine and physiology [8]. Past COVID-19 infection can affect the heart [4,9]. Measured HR_max_ compared to age-predicted HR_max_ is the criterion used to assess whether the cardiopulmonary exercise test (CPET) outcome is maximal [10]. Maximal exercise ventilation (VE_max_) is the most important contributor to VO_2max_ and could be a limiting factor in CRF [11].

COVID-19 impairs lung function and respiratory muscle strength and promotes endothelial dysfunction, systemic inflammation, cytokine-mediated injury, myocardial injury, and immune-mediated effects [12,13,14,15,16]. Moreover, hypertension could aggravate the course of COVID-19 [17]. All of the factors described above lead to cardiovascular, respiratory, and vascular complications and, ultimately, aggravate CRF. Therefore, it remains crucial to assess changes in VO_2max_, HR_max,_ and VE_max_ during infection [18], additionally to other markers such as cardiovascular calcifications [17]. Negative changes have been noted even after a mild course, typical for athletes [2,19]. Higher CRF is inversely linked with the risk of hospitalization due to severe COVID-19 in athletes [18]. Grading the impairment in CRF due to infection is crucial for targeting treatment, adjusting safe exercise intensity, and developing a return-to-sport strategy [20].

Assessing changes in CRF before and after COVID-19 and comparing them to a reference population of healthy individuals is problematic [21]. There are noticeable limitations in recruiting athletes who have retrospective access to CPET data and reliable confirmation of COVID-19 infection [22]. Furthermore, it is difficult to recruit large samples and keep within the restricted timeframe between pre-COVID-19 and post-COVID-19 CPET to minimize the impact of detraining and focus on the impact of infection [3].

The degree of aggravation in CRF due to infection with COVID-19 is still controversial. Therefore, in this systematic review, we performed a meta-analysis of cross-sectional and longitudinal studies comparing VO_2max_, HR_max,_ and VE_max_ within the same athletes before and after COVID-19 infection, as well as between healthy, uninfected athletes and athletes who suffered from COVID-19. We aimed to assess how having had COVID-19 infection impacts CRF in athletes and what is the degree of such an impairment.

## 2. Materials and Methods

### 2.1. Eligibility Criteria

We performed this systematic review and meta-analysis according to the PRISMA 2020 Statement [23] (Supplementary Material S1, Table S1 and Supplementary Material S2, Table S2). We included observational studies and case series with at least 5 participants who were athletes at various competitive levels. We considered only papers that reported the VO_2max_ (either absolute or relative value) of both participants previously infected with COVID-19 and those not infected (or if pre-infection VO_2max_ values were reported). We considered only VO_2max_ (alternatively named as peak VO_2_) values obtained from the CPET. Other forms of testing, such as submaximal VO_2_, or VO_2max_ estimates, were not considered. We considered only studies including athletes aged 18 or older, of both sexes. An athlete was defined according to the Bethesda Conference guidelines as “one who participates in an organized team or individual sport that requires regular competition against others as a central component, places a high premium on excellence and achievement, and requires some form of systematic (and usually intense) training”. We excluded case reports, commentaries, conference abstracts, editorials, guidelines (also statements, consensuses, and position papers), letters to editors, protocols, and reviews (systematic reviews, narrative reviews, and meta-analyses). We excluded studies not written in English and studies in which COVID-19 infection was self-reported by participants (not confirmed by PCR, antigen test, or other reliable method).

### 2.2. Information Sources

We searched five databases: Embase, Medline Ultimate, PubMed, Scopus, and Web of Science. Each database was searched from inception to 9 August 2025. Gray literature searches, citation tracking or manual bibliography reviews were not performed.

### 2.3. Search Strategy

For each database, we used the following search strategy: “(COVID-19 or coronavirus or SARS-CoV-2 or SARSCoV2 or SARSCoV-2 or SARS-CoV2) and (VO2max or VO2 max or V02max or V02 max or maximal oxygen consumption or maximal oxygen uptake or maximal aerobic capacity or cardiopulmonary exercise test or CPET or cardiorespiratory fitness) and (athlete or athletes or player or sportsmen or sportswomen)”. We did not use any filters.

### 2.4. Selection Process

We used the EndNote (version 20.6) automation tool to identify duplicates. Afterwards, GP and PK screened all the records independently to identify the remaining duplicates. Next, GP and PK screened independently and excluded records based on article type. Then, the remaining articles were screened independently by GP and PK by title and abstract for meeting the eligibility criteria. Eventually, GP and PK assessed full-text articles for inclusion. At each step, all the discrepancies between GP and PK were identified and discussed until consensus was reached.

### 2.5. Data Collection Process

Working together, GP and PK extracted data from each eligible study and entered it into the data extraction form using Cochrane RevMan Version 9.8.2.

### 2.6. Data Items

We collected all available data for the post-COVID and control groups (either the same athletes before COVID-19 infection or non-infected healthy controls) for the following outcomes: relative VO_2max_ [mL·kg^−1^·min^−1^], absolute VO_2max_ [L·min^−1^], HR_max_ [bpm], and VE_max_ [L·min^−1^]. For each variable, we collected the mean values, standard deviation, and the number of participants in each group. In the same manner, for each group we also collected data regarding body mass index (BMI) [kg·m^−2^] and age [years].

If available, we also extracted the following covariates for each group in all studies: competition level (recreational, competitive, or mixed), CPET modality (cycle ergometer, treadmill, other, mixed), sex distribution (only male, mixed, not reported), comparison type (post-COVID athletes vs. healthy reference athletes, post-COVID athletes vs. the same athletes before infection), symptoms in athletes during COVID-19 infection (only symptomatic athletes, only asymptomatic athletes, mixed, not reported), time from COVID-19 infection to CPET (up to 1 month after infection, more than 1 month after infection, and not reported). “Healthy reference athlete” was defined as an individual who was not infected/diagnosed with COVID-19 in the original study. The non-COVID group was defined by collective comparisons to healthy reference athletes and pre-COVID values in infected athletes.

### 2.7. Endpoint Definition

The primary outcome was the mean difference [MD] in relative VO_2max_ between post-COVID athletes and the non-COVID group (collective comparisons to healthy reference and pre-COVID values in the same infected athletes). The secondary outcomes included the following, all compared between the groups as per the primary outcome: (1) mean difference in absolute VO_2max_, (2) mean difference in HR_max_, and (3) mean difference in VE_max_.

### 2.8. Study Risk of Bias Assessment

The risk of bias for the primary outcome was assessed by GP and PK working together with the use of JBI’s critical appraisal tool for analytical cross-sectional studies. Risk of bias was assessed in the following domains: (1) eligibility and sampling, (2) population and setting reporting, (3) exposure ascertainment, (4) outcome definition (VO_2max_), (5) confounders—identification, (6) confounders—control/adjustment, (7) outcome measurement validity/reliability (CPET description), and (8) statistical methods appropriateness. The assessment can be found in all forest plots regarding relative VO_2max_ and in Supplementary Material S3, Figure S13.

### 2.9. Effect Measures

We compared all continuous variables (relative VO_2max_, absolute VO_2max_, HR_max_, VE_max_, age, and BMI) by calculating the mean differences with 95% CI.

### 2.10. Synthesis Report

At the stage of the synthesis process, we considered all eligible studies that reported the outcome of interest. If the number of participants (e.g., symptomatic) was not given, we calculated it based on percentages and sample size, if possible. Since not all studies reported continuous variables as means and standard deviations, we made an assumption that the median is equal to the mean, and the standard deviation was calculated by dividing by 1.35 the difference between the upper and lower limits of the interquartile range. In those cases, we have unequivocally made a footnote remark on each relevant figure presenting this data.

For each data synthesis, we used forest plots to present the data from individual studies and syntheses. Next to each eligible study, we made a visual presentation of the risk of bias assessment for the primary outcome. Studies were sorted by the size of the study effect within each plot.

Meta-analyses were performed using Cochrane RevMan Version 9.8.2. We used a random effects model for each analysis. The inverse variance method was used to calculate the overall effect, and the between-study variance was estimated with the Restricted Maximum Likelihood method. Heterogeneity was formally assessed with the chi-square test, and I^2^ statistics were used to quantify it.

We performed subgroup analyses for relative VO_2max_, absolute VO_2max_, HR_max_, and VE_max_ to explore possible causes of the heterogeneity. We analyzed subgroups for the following covariates: competition level, CPET modality, sex distribution, comparison type, symptoms in athletes during COVID-19 infection, and time from COVID-19 infection to CPET.

For relative VO_2max_, to perform a sensitivity analysis, we sequentially excluded each study and assessed its influence on the overall effect. We also performed sensitivity analysis for relative VO_2max_ by excluding studies with a high risk of bias in 4 or more domains.

### 2.11. Reporting Bias Assessment

For relative VO_2max_, absolute VO_2max_, HR_max_, and VE_max,_ we generated funnel plots and assessed them visually to evaluate the potential publication bias.

### 2.12. Certainty Assessment

The certainty of evidence was assessed for relative VO_2max_, absolute VO_2max_, HR_max_, and VE_max_ using five GRADE considerations: risk of bias, inconsistency, indirectness, imprecision, and publication bias. The certainty of evidence was assessed as high, moderate, low, or very low. The summary of findings table was prepared with GRADEpro GDT software [24].

## 3. Results

### 3.1. Study Selection

**Figure 1:**
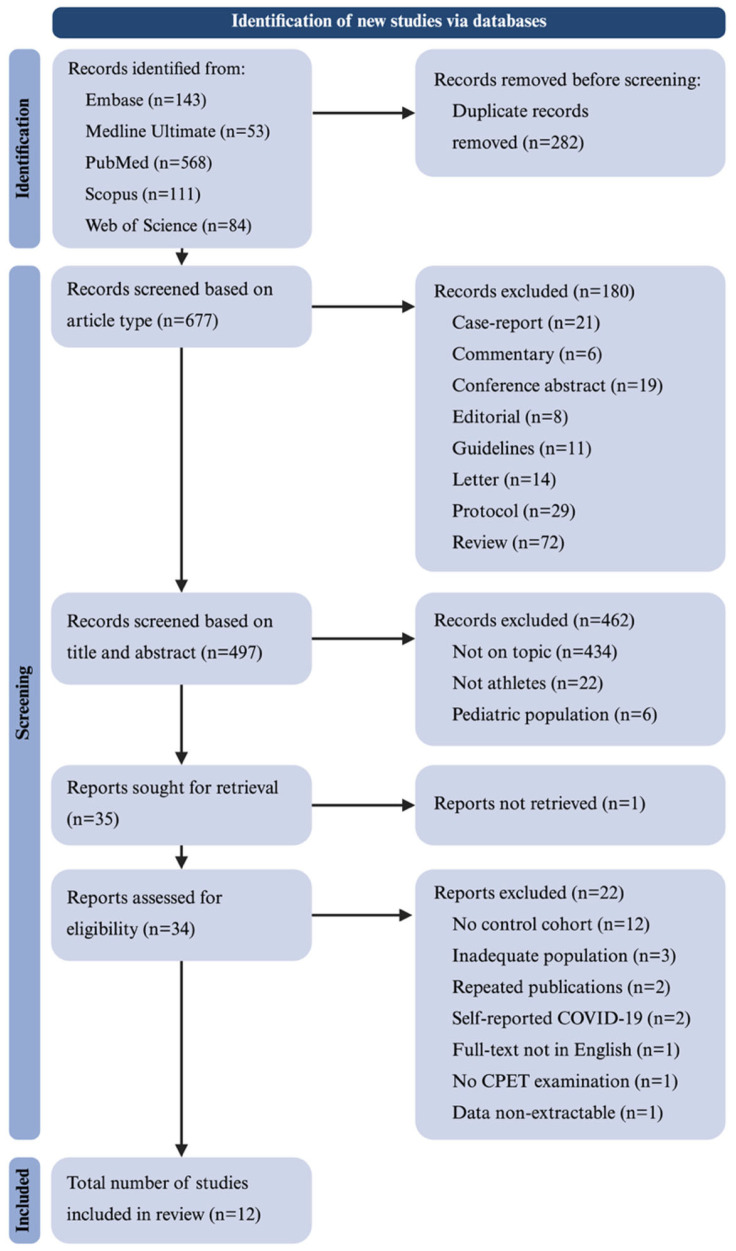
The flowchart for the selection process. Abbreviations: COVID-19—coronavirus disease 2019; CPET—cardiopulmonary exercise test; n—number of studies.

The initial search yielded a total of 959 records. After we removed 282 duplicates, we screened the remaining 677 records by article type. We excluded 180 records based on article type. The remaining 497 records entered the screening stage based on title and abstract. It yielded 35 articles that were acknowledged as appropriate and were chosen for full-text search. One study was not retrieved in complete form. We assessed the complete data reports for the remaining 34 articles and we evaluated them for eligibility, excluding 22 studies due to (1) no control group for outcome comparison [2,3,25,26,27,28,29,30,31,32,33,34], (2) inadequate population [35,36,37], (3) repeated publications [38,39], (4) self-reported COVID-19 infection [40,41], (5) full-text not in English [42], (6) no CPET examination [43], and (7) data non-extractable [44]. Eventually, twelve studies were included [21,45,46,47,48,49,50,51,52,53,54,55] (Figure 1).

### 3.2. Study Characteristics

The characteristics of each included study are reported in Table 1. There was no association between having had COVID-19 infection and age (MD: 0.60 years, 95%CI: [−0.32, 1.51], *p* = 0.20, I^2^ = 19%, Supplementary Material S3, Figure S1) or body mass index (MD: 0.19 kg·m^−2^, 95%CI: [−0.53, 0.91], *p* = 0.60, I^2^ = 64%, Supplementary Material S3, Figure S2).

### 3.3. Relative VO_2max_

**Figure 2:**
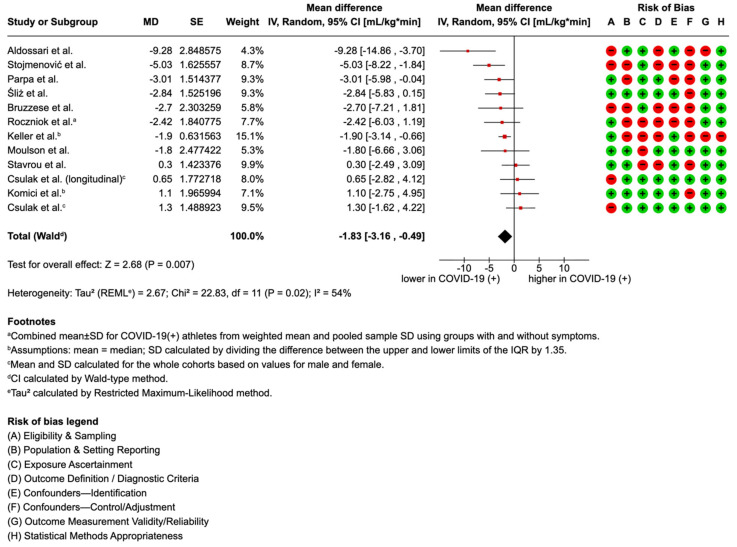
Forest plot comparing relative VO_2max_ between post-COVID and non-COVID groups. COVID-19 infection was associated with lower relative VO_2max_ (MD: −1.83 mL·kg^−1^·min^−1^, 95%CI: [−3.16, −0.49], *p* = 0.007, I^2^ = 54%). A red circle means high risk of bias, while a green circle means low risk of bias [21,45,46,47,49,50,51,52,53,54,55].

Eleven studies (one study with two different comparisons), including a total of 1583 athletes, reported relative VO_2max_. Having had COVID-19 infection was associated with lower relative VO_2max_ (MD: −1.83 mL·kg^−1^·min^−1^, 95%CI: [−3.16, −0.49], *p* = 0.007, I^2^ = 54%, Figure 2).

The complete subgroup analysis can be found in Supplementary Material S3, Figure S3A–F. In the subgroup of studies including only competitive athletes, there was no association between relative VO_2max_ and COVID-19 infection (MD: −1.32 mL·kg^−1^·min^−1^, 95%CI: [−2.70, 0.05], *p* = 0.06, I^2^ = 50%), while in the subgroups of studies including only recreational athletes (MD: −9.28 mL·kg^−1^·min^−1^, 95%CI: [−14.86, −3.70], *p* = 0.001, I^2^ not applicable—only one study) or mixed athletes (MD: −2.55 mL·kg^−1^·min^−1^, 95%CI: [−5.10, −0.01], *p* = 0.05, I^2^ = 0%) COVID-19 infection was associated with lower relative VO_2max_ (Supplementary Material S3, Figure S3A). The overall effect remained significant in the comparison between athletes who had COVID-19 infections and the same athletes before infection (MD: −2.67 mL·kg^−1^·min^−1^, 95%CI: [−4.46, −0.88], *p* = 0.003, I^2^ = 30%), while it was insignificant in the comparison between athletes who had COVID-19 infections and healthy reference athletes (MD: −1.29 mL·kg^−1^·min^−1^, 95%CI: [−3.20, 0.61], *p* = 0.18, I^2^ = 62%, Supplementary Material S3, Figure S3D). The association with relative VO_2max_ was also significant in the subgroup of studies including only symptomatic athletes during COVID-19 infection (MD: −2.68 mL·kg^−1^·min^−1^, 95%CI: [−5.21, −0.15], *p* = 0.04, I^2^ = 0%) while it was insignificant in the subgroup of studies including both symptomatic and asymptomatic athletes during COVID-19 (MD: −0.81 mL·kg^−1^·min^−1^, 95%CI: [−2.29, 0.66], *p* = 0.28, I^2^ = 35%, Supplementary Material S3, Figure S3E). Finally, the observed association with relative VO_2max_ remained significant in the subgroup of studies in which CPET was performed more than 1 month after COVID-19 infection (MD: −3.24 mL·kg^−1^·min^−1^, 95%CI: [−4.87, −1.61], *p* < 0.0001, I^2^ = 0%), while it was insignificant in the subgroup of studies in which CPET was performed up to 1 month after COVID-19 infection (MD: −0.65 mL·kg^−1^·min^−1^, 95%CI: [−2.75, 1.44], *p* = 0.54, I^2^ = 57%, Supplementary Material S3, Figure S3F). Nevertheless, the significant subgroup interaction was observed only for analysis based on competition level (*p* = 0.02).

The sensitivity analysis revealed no influence of the removal of any single study (Supplementary Material S3, Figure S4A–L); however, the removal of studies with high risk of bias in four or more domains influenced the overall effect, making it insignificant (MD: −0.62 mL·kg^−1^·min^−1^, 95%CI: [−2.11, 0.88], *p* = 0.42, I^2^ = 31%, Supplementary Material S3, Figure S5).

Funnel plot was slightly asymmetric on visual inspection (Supplementary Material S3, Figure S9). The certainty of evidence was assessed as very low (Supplementary Material S3, Table S3).

### 3.4. Absolute VO_2max_

**Figure 3:**
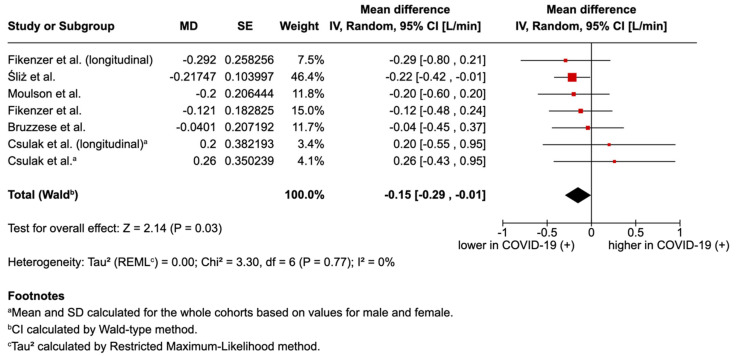
Forest plot comparing absolute VO_2max_ between post-COVID and non-COVID groups. COVID-19 infection was associated with lower absolute VO_2max_ (MD: −0.15 L·min^−1^, 95%CI: [−0.29, −0.01], *p* = 0.03, I^2^ = 0%) [21,46,47,48,53].

Five studies (two studies with two different comparisons), including a total of 180 athletes, reported absolute VO_2max_. Having had COVID-19 infection was associated with lower absolute VO_2max_ (MD: −0.15 L·min^−1^, 95%CI: [−0.29, −0.01], *p* = 0.03, I^2^ = 0%, Figure 3).

The complete subgroup analysis is presented in Supplementary Material S3, Figure S6A–F. In the subgroup of studies including only competitive athletes, there was no association between COVID-19 and absolute VO_2max_ (MD: −0.07 L·min^−1^, 95%CI: [−0.28, 0.15], *p* = 0.55, I^2^ = 0%), while in the subgroup of studies including both recreational and competitive athletes, COVID-19 was associated with lower absolute VO_2max_ (MD: −0.21 L·min^−1^, 95%CI: [−0.40, −0.03], *p* = 0.02, I^2^ = 0%) (Supplementary Material S3, Figure S6A). The association between COVID-19 and absolute VO_2max_ remained significant in the comparison between athletes who had COVID-19 infection and the same athletes before infection (MD: −0.17 L·min^−1^, 95%CI: [−0.34, −0.01], *p* = 0.04, I^2^ = 0%), while it was insignificant in the comparison between athletes who had COVID-19 infection and healthy reference athletes (MD: −0.10 L·min^−1^, 95%CI: [−0.35, 0.15], *p* = 0.43, I^2^ = 0%, Supplementary Material S3, Figure S6D). Last, the observed association between COVID-19 and absolute VO_2max_ remained significant in the subgroup of studies in which CPET was performed more than 1 month after COVID-19 infection (MD: −0.21 L·min^−1^, 95%CI: [−0.40, −0.03], *p* = 0.02, I^2^ = 0%), while it was insignificant in the subgroup of studies in which CPET was performed up to 1 month after COVID-19 infection (MD: −0.07 L·min^−1^, 95%CI: [−0.28, 0.15], *p* = 0.55, I^2^ = 0%, Supplementary Material S3, Figure S6F). However, none of these subgroup interactions reached statistical significance.

The funnel plot was slightly asymmetric on visual inspection (Supplementary Material S3, Figure S10); however, in our assessment, adjusting for this publication bias effect might even increase the magnitude of the overall effect. The certainty of evidence was assessed as very low (Supplementary Material S3, Table S3).

### 3.5. HR_max_

**Figure 4:**
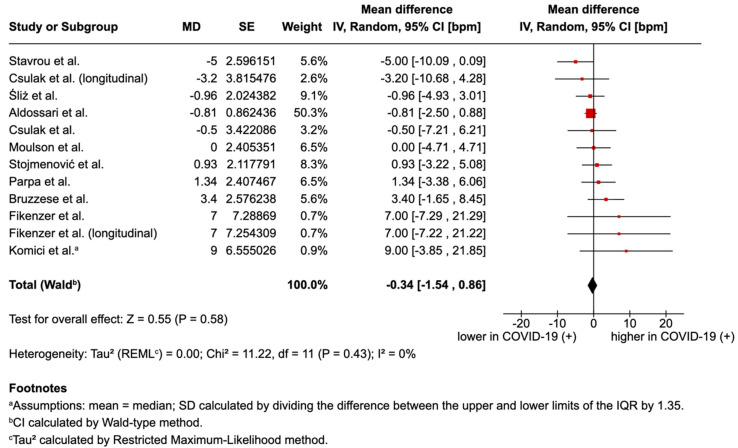
Forest plot comparing HR_max_ between post-COVID and non-COVID groups. There was no association between COVID-19 infection and HR_max_ (MD: −0.34 bpm, 95%CI: [−1.54, 0.86], *p* = 0.58, I^2^ = 0%) [21,45,46,47,48,50,51,53,54,55].

Ten studies (two studies with two different comparisons), including a total of 345 athletes, reported HR_max_. Having had COVID-19 was not associated with a difference in HR_max_ (MD: −0.34 bpm, 95%CI: [−1.54, 0.86], *p* = 0.58, I^2^ = 0%, Figure 4).

The complete subgroup analysis can be found in Supplementary Material S3, Figure S7A–F. It did not reveal any findings regarding the mean difference in HR_max_ within the predefined subgroups.

The funnel plot was slightly asymmetric on visual inspection (Supplementary Material S3, Figure S11). The certainty of evidence was assessed as very low (Supplementary Material S3, Table S3).

### 3.6. VE_max_

**Figure 5:**
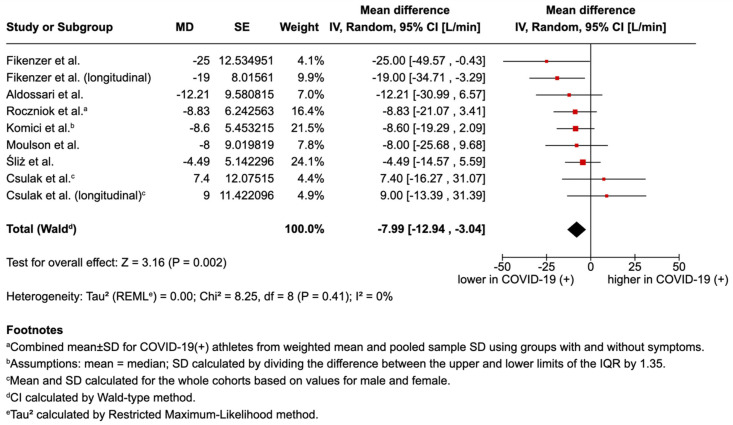
Forest plot comparing VE_max_ between post-COVID and non-COVID groups. COVID-19 infection was associated with lower VE_max_ (MD: −7.99 L·min^−1^, 95%CI: [−12.94, −3.04], *p* = 0.002, I^2^ = 0%) [21,45,47,48,50,52,53].

Seven studies (two studies with two different comparisons), including a total of 297 athletes, reported VE_max_. Having had COVID-19 was associated with lower VE_max_ (MD: −7.99 L·min^−1^, 95%CI: [−12.94, −3.04], *p* = 0.002, I^2^ = 0%, Figure 5).

The complete subgroup analysis is presented in Supplementary Material S3, Figure S8A–F. In the subgroup of studies including only competitive athletes, the association between COVID-19 and VE_max_ remained significant (MD: −8.89 L·min^−1^, 95%CI: [−15.22, −2.55], *p* = 0.006, I^2^ = 0%), while in the subgroups of studies including only recreational athletes (MD: −12.21 L·min^−1^, 95%CI: [−30.99, 6.57], *p* = 0.20, I^2^ not applicable—only one study) or mixed athletes (MD: −5.35 L·min^−1^, 95%CI: [−14.11, 3.40], *p* = 0.23, I^2^ = 0%) there was no association between COVID-19 and VE_max_ (Supplementary Material S3, Figure S8A). The association with VE_max_ was significant in the studies including only male athletes (MD: −20.74 L·min^−1^, 95%CI: [−33.98, −7.51], *p* = 0.002, I^2^ = 0%), while insignificant in studies including participants of both sexes (MD: −3.73 L·min^−1^, 95%CI: [−10.86, 3.41], *p* = 0.31, I^2^ = 0%, Supplementary Material S3, Figure S8C). The association with VE_max_ remained significant in the comparison between athletes who underwent COVID-19 infection and healthy reference athletes (MD: −8.94 L·min^−1^, 95%CI: [−15.28, −2.60], *p* = 0.006, I^2^ = 0%), while it was insignificant in the comparison between athletes who underwent COVID-19 infection and the same athletes before infection (MD: −6.24 L·min^−1^, 95%CI: [−19.59, 7.12], *p* = 0.36, I^2^ = 56%, Supplementary Material S3, Figure S8D). The association with VE_max_ was insignificant in the subgroup of studies including only symptomatic athletes during COVID-19 infection (MD: −8.00 L·min^−1^, 95%CI: [−25.68, 9.68], *p* = 0.38, I^2^ not applicable—only one study), while it was significant in the subgroup of studies including both symptomatic and asymptomatic athletes during COVID-19 (MD: −8.89 L·min^−1^, 95%CI: [−15.22, −2.55], *p* = 0.006, I^2^ = 0%, Supplementary Material S3, Figure S8E). Finally, the overall effect regarding VE_max_ remained significant in the subgroup of studies in which CPET was performed more than 1 month after COVID-19 infection (MD: −7.24 L·min^−1^, 95%CI: [−13.90, −0.59], *p* = 0.03, I^2^ = 0%) while it was insignificant in the subgroup of studies in which CPET was performed up to 1 month after COVID-19 infection (MD: −8.21 L·min^−1^, 95%CI: [−19.28, 2.87], *p* = 0.15, I^2^ = 46%, Supplementary Material S3, Figure S8F). However, none of these subgroup interactions reached statistical significance.

The funnel plot was symmetric on visual inspection (Supplementary Material S3, Figure S12). The certainty of evidence was assessed as very low (Supplementary Material S3, Table S3).

## 4. Discussion

The main findings of our study are: (i) COVID-19 infection in athletes may be associated with reduced VO_2max,_ and the difference may be more pronounced within the same athletes than when compared to healthy reference subjects; (ii) the association between COVID-19 infection and lower VO_2max_ was more pronounced among recreational than competitive athletes; and (iii) COVID-19 infection was not associated with changes in HR_max_, but was associated with lower VE_max_.

No meta-analysis has investigated the decline in VO_2max_, HR_max,_ and VE_max_ due to COVID-19 in athletes, considering within-subject and between-population analyses. To date, meta-analyses have evaluated the impact of COVID-19 on VO_2max_ but have focused on untrained subjects often described as ‘survivors’ [56,57]. Their results should not be extrapolated to the athletic population, as athletes rarely experience severe symptoms and require hospitalization [3]. Some meta-analyses included only data from the very early stages and omitted studies published after 2023 [57,58]. Other researchers have investigated populations of athletes, but they only screened one database, which posed the risk of omission [58]. A recent systematic review summarized the population of athletes, but did not perform a meta-analysis and focused only on football players [59]. Finally, there were studies assessing the impact of lockdown and imposed restrictions, but not direct infection, which might be the strongest harmful factor [60,61]. To the best of our knowledge, this is the first such meta-analysis of changes in CRF in athletes due to infection with COVID-19 including within-athlete and between-athlete comparisons.

### 4.1. Interpretation

It should be underscored that the certainty of evidence was very low, both for primary and secondary outcomes, mainly due to the high risk of bias and observational design of studies. The primary outcome, VO_2max_, was sensitive to study quality, and the association became statistically nonsignificant after excluding low-quality studies. While the exploratory subgroup analyses in Supplementary Material S3 emerged as clinically interesting, the overall interpretation is impacted by the small and low-powered sample sizes. Therefore, they should be treated as exploratory and hypothesis generating.

The included studies rarely controlled for confounders, such as the effects of detraining or limited physical activity during recovery from infection. It is well known that exercise induces cardiac adaptations that are visible not only in echocardiography or ECG, but in CPET too [62]. However, exercise-induced cardiac adaptations could mirror certain cardiovascular diseases (e.g., hypertrophic cardiomyopathy vs. athlete’s heart) in training populations [63]. Periods of detraining are not rare among athletes, who often undergo them during infections, musculoskeletal disorders, or scheduled deloads [64]. During training abstinence, the reverse adaptations occurred [65]. Lack of physical activity leads to reduced dimensions of right and left ventricles, which translates into reduced sports performance, especially in endurance-based disciplines (running, cycling, soccer, etc.). CRF also decreases during detraining in parallel to infection-related aggravation [66]. Therefore, the reduction in CRF could be mediated in part by the infection itself and in part by detraining [64]. Differentiation between detraining changes and infection-related changes is still ambiguous and largely based on consensus statements, with a knowledge gap for further studies [20]. Ultimately, despite statistical significance, the clinical impact of the reduction in VO_2max_ is also low (−1.83 mL·kg^−1^·min^−1^) especially among elite athletes, and day-to-day and diurnal performance could impact CPET results to a similar extent [67].

### 4.2. Impact of COVID-19 Infection on VO_2max_

The associations between COVID-19 and VO_2max_ that we observed are consistent with those in untrained populations in some areas. We noted a significant reduction in VO_2max_ when the infection was symptomatic, similar to Chuatrakoon et al. and Gomes-Neto et al. [56,57]. However, among athletes, the decrease in VO_2max_ was not significant compared to their healthy counterparts, which is in contrast to untrained populations. Although Lopes et al. only included studies from PubMed up to 2023, they also observed a reduction in aerobic fitness [58]. Hasler et al. reported lower VO_2max_ in squad members than in non-squad members. To some extent, this mirrors our results among competitive and recreational athletes [44].

Both relative and absolute VO_2max_ were consistently associated with having had a COVID-19 infection in the athletic population. Studies included in our meta-analysis investigated endurance and team-sport athletes. The observed relationship is understandable, as in those disciplines athletes typically have a healthy weight and normal BMI [68]. The relationship could be different among strength athletes, where an elevated BMI is sometimes desirable and VO_2max_ is lower and its impact on performance is less important [69]. Notably, the association between infection and VO_2max_ was strongly dependent on the quality of the included studies. The sensitivity analysis, which excluded all the high-risk studies, changed the reduction in VO_2max_ to insignificant (*p* = 0.42).

### 4.3. Impact of COVID-19 Infection on HR_max_ and VE_max_

Although cardiac involvement, including myocarditis, may occur during COVID-19 infection, we did not observe a significant association with a change in HR_max_ [70,71]. Hasler et al. underscored that HR_max_ rose during both maximal and submaximal exertion [44]. Therefore, the assessment of the circulatory system in athletes after an infection should not be neglected based only on a possible lack of change in HR_max,_ and this is in accordance with current recommendations [22].

It can be assumed that the imposed restrictions and detraining also affected the CRF of healthy athletes who were unable to train, even if they had not been infected [60,64]. Given that significant reductions occurred for both VO_2max_ and VE_max_, but not HR_max_, it can be hypothesized that COVID-19 affects the respiratory system more than the circulatory system among athletes. However, this should be interpreted with caution, and the low quality of studies reporting HR_max_ must be considered.

### 4.4. Perspectives and Directions for Future Studies

A follow-up of the athletes with the most severe COVID-19 cardiopulmonary complications should assess their current CRF status and whether the past cardiac involvement has healed completely. It would also be interesting to compare the consequences of COVID-19 in athletes with flu, cold, pneumonia, etc., which are much more common in athletes and typically occur in the autumn/winter season [72]. In recent years, stress echocardiography has gained attention in athlete care studies [66]. We focused only on raw CPET as a major diagnostic tool. Therefore, more robust studies pairing CPET and stress echocardiography and other diagnostic methods would be warranted. The included studies recruited athletes of moderate age and omitted pediatric athletes [73,74]. Normative data for CPET varied throughout the lifespan and depend on the age of the participants [75,76]. Ultimately, future studies should check whether COVID-19-related impairment from moderate-age athletes also translates to pediatric and master’s athletes.

### 4.5. Limitations

Our meta-analysis has several limitations. First, Csulak et al. and Fikenzer et al. compared athletes both before and after COVID-19 infection and athletes after COVID-19 infection to a healthy reference group [47,48]. We included both parts of those studies, as cross-sectional (for comparison between post-COVID-19 and healthy athletes) and longitudinal (for comparison before and after COVID-19). Therefore, these studies were listed twice in different categories, which led to a slight cohort overlap and data duplication related to one arm of the comparisons. Nevertheless, given the weights assigned to these studies and the direction of effect, this should not materially affect the overall conclusions. Second, there was high heterogeneity of included studies, which limits the generalizability of conclusions. This was especially visible in terms of the interval between infection and CPET, where high variability from 10 days to 12 months emerged. This posed a risk of ambiguity as to whether the consequences resulted directly from infection or detraining and interruption of physical activity. We did not extend our study with meta-regression analysis to check where the CRF impairment results came from. Two studies did not even report the interval. Third, several of the included studies were of low quality, while six of them poorly defined primary (VO_2max_) and secondary (HR_max_ and VE_max_) outcomes. Fourth, we focused on VO_2max_, HR_max,_ and VE_max,_ and did not sub-analyze lung function or respiratory muscle strength. Fifth, participants achieved their CRF variables via different protocols and measuring methods. Sixth, the study population consisted predominantly of males, and females were underrepresented. This problem has already been noted, and there have been recommendations to include a larger percentage of females in future research [77]. Seventh, the majority of studies included limited samples of up to 100 subjects. Only Keller et al. have more than 1000 participants [49]. Smaller studies tend to report stronger effects, and one large study could dominate with a consolidated outcome. Eighth, the risk of bias was not assessed independently, which may introduce reviewer bias. Ninth, the certainty of evidence was very low and some funnel plots were suggestive of possible publication bias. The listed limitations suggest caution in interpreting the results and the need for further research with more transparent methodology.

## 5. Conclusions

In conclusion, there was a significant association between COVID-19 and reduced VO_2max_ and VE_max_, but not HR_max_. A more pronounced decline in VO_2max_ was observed in recreational athletes than in competitive athletes. Both lower relative and lower absolute VO_2max_ were associated with COVID-19 infection within the same athletes, but not when compared to healthy reference athletes. The impairment in CRF should be considered during clinical decision-making in order to inform adjustments to training schedules and intensity. Clinicians should account for possible CRF impairment due to COVID-19 infection when making shared decisions regarding allowing athletes to return to training or competition.

## Figures and Tables

**Table 1:** Baseline characteristics of included studies.

Ref.	Population	Competition Level	Time from COVID-19 Infection to CPET	CPET Modality	Sex Distribution (Female)	Symptomatic
[45]	42 athletes	Recreational	≥12 months	Treadmill	COVID(+): 4/21 COVID(−): 3/21	n/d
[46]	10 football players	Competitive	10 days	Treadmill	n/d ^1^	5/10
[47]	46 swimmers	Competitive	10–14 days	Treadmill	COVID(+): 7/14 COVID(−): 14/32	12/14
[48]	12 handball players	Competitive	19 ± 7 days	Cycle ergometry	COVID(+): 0/8 COVID(−): 0/4	7/8
[49]	1200 athletes	Competitive	n/d	Treadmill and cycle ergometry	COVID(+): 35/157 COVID(−): 376/1043	141/157
[50]	35 soccer players	Competitive	≤30 days ^2^	Treadmill	n/d ^1^	22/24
[21]	63 athletes	Competitive and recreational	3.0 ± 2.1 months	Treadmill and cycle ergometry	COVID(+): 9/21 COVID(−): 18/42	21/21 ^3^
[51]	21 soccer players	Competitive	60 days	Treadmill	n/d ^1^	21/21
[52]	50 ice hockey players	Competitive	n/d	Cycle ergometry	n/d ^1^	9/37
[53]	49 endurance athletes	Competitive and recreational	155.27 ± 82.52 days	Treadmill and cycle ergometry	6/49	n/d
[54]	40 soccer players	Competitive	30 days ^2^	Treadmill	COVID(+): 0/20 COVID(−): 0/20	n/d
[55]	27 basketball players	Competitive	2 weeks	Treadmill	0/27	n/d

Abbreviations: COVID—coronavirus disease; CPET—cardiopulmonary exercise test; n/d—no data. ^1^ Most likely only male, but not unequivocally stated. ^2^ Not unequivocally stated. ^3^ All symptomatic also during CPET.

## Data Availability

The raw data supporting the conclusions will be made available on reasonable request to the corresponding author.

## Supplementary Materials

The following supporting information can be downloaded at https://www.mdpi.com/article/10.3390/jcm15135133/s1, Supplementary Material S1. Table S1: PRISMA abstract checklist; Supplementary Material S2: Table S2: PRISMA checklist; Supplementary Material S3: Figure S1: Forest plot comparing age between post-COVID athletes and a non-COVID group; Figure S2: Forest plot comparing BMI between post-COVID athletes and a non-COVID group; Figure S3. A: Forest plot presenting the relative VO_2max_ subgroup analysis based on competition level; B: Forest plot presenting the relative VO_2max_ subgroup analysis based on CPET modality; C: Forest plot presenting the relative VO_2max_ subgroup analysis based on sex distribution; D: Forest plot presenting the relative VO_2max_ subgroup analysis based on comparison type; E: Forest plot presenting the relative VO_2max_ subgroup analysis based on the presence of symptoms in athletes during COVID-19 infection; F: Forest plot presenting the relative VO_2max_ subgroup analysis based on time elapsed from COVID-19 infection to CPET; Figure S4. A–L: Forest plots presenting the sensitivity analyses regarding relative VO_2max_ performed by removing every single study consecutively; Figure S5: Forest plot presenting the sensitivity analysis regarding VO_2max_ performed by removing studies with a high risk of bias in 4 or more domains; Figure S6. A: Forest plot presenting the absolute VO_2max_ subgroup analysis based on competition level; B: Forest plot presenting the absolute VO_2max_ subgroup analysis based on CPET modality; C: Forest plot presenting the absolute VO_2max_ subgroup analysis based on sex distribution; D: Forest plot presenting the absolute VO_2max_ subgroup analysis based on comparison type; E: Forest plot presenting the absolute VO_2max_ subgroup analysis based on the presence of symptoms in athletes during COVID-19 infection; F: Forest plot presenting the absolute VO_2max_ subgroup analysis based on time elapsed from COVID-19 infection to CPET; Figure S7. A: Forest plot presenting the HR_max_ subgroup analysis based on competition level; B: Forest plot presenting the HR_max_ subgroup analysis based on CPET modality; C: Forest plot presenting the HR_max_ subgroup analysis based on sex distribution; D: Forest plot presenting the HR_max_ subgroup analysis based on comparison type; E: Forest plot presenting the HR_max_ subgroup analysis based on the presence of symptoms in athletes during COVID-19 infection; F: Forest plot presenting the HR_max_ subgroup analysis based on time elapsed from COVID-19 infection to CPET; Figure S8. A: Forest plot presenting the VE_max_ subgroup analysis based on competition level; B: Forest plot presenting the VE_max_ subgroup analysis based on CPET modality; C: Forest plot presenting the VE_max_ subgroup analysis based on sex distribution; D: Forest plot presenting the VE_max_ subgroup analysis based on comparison type; E: Forest plot presenting the VE_max_ subgroup analysis based on the presence of symptoms in athletes during COVID-19 infection; F: Forest plot presenting the VE_max_ subgroup analysis based on time elapsed from COVID-19 infection to CPET; Figure S9: Funnel plot assessing the publication bias for relative VO_2max_; Figure S10: Funnel plot assessing the publication bias for absolute VO_2max_; Figure S11: Funnel plot assessing the publication bias for HR_max_; Figure S12: Funnel plot assessing the publication bias for VE_max_; Figure S13: Risk of bias assessment of included studies; Table S3: The summary of findings in terms of the certainty of evidence regarding relative VO_2max_, absolute VO_2max_, HR_max_, and VE_max_.
