# Supplementary material for: COACH Study: COVID-19 Influence on Cardiorespiratory Fitness in Athletes—A Systematic Review and Meta-Analysis
Source: J Clin Med. 2026 Jul 1;15(13):5133. doi: 10.3390/jcm15135133 (PMC13362685; doi:10.3390/jcm15135133)
Supplement: Supplementary file 1 [file jcm-15-05133-s001.zip › Supplementary Material S3.pdf]

Supplementary Material S3

Title: COACH Study: COVID-19 Influence on Cardiorespiratory Fitness in Athletes—A Systematic Review and Meta-Analysis

Table of contents

Figure S1: Forest plot comparing age between post-COVID athletes and a non-COVID group. 4

Figure S2: Forest plot comparing BMI between post-COVID athletes and a non-COVID group...... 5

Figure S3A: Forest plot presenting the relative VO_{2max} subgroup analysis based on competition level. 6

Figure S3B: Forest plot presenting the relative VO_{2max} subgroup analysis based on CPET modality. 7

Figure S3C: Forest plot presenting the relative VO_{2max} subgroup analysis based on sex distribution. 8

Figure S3D: Forest plot presenting the relative VO_{2max} subgroup analysis based on comparison type. 9

Figure S3E: Forest plot presenting the relative VO_{2max} subgroup analysis based on the presence of symptoms in athletes during COVID-19 infection. 10

Figure S3F: Forest plot presenting the relative VO_{2max} subgroup analysis based on time elapsed from COVID-19 infection to CPET. 11

Figures S4A to S4L: Forest plots presenting the sensitivity analyses regarding relative VO_{2max} performed by removing every single study consecutively. 12

Figure S4A 12

Figure S4B 13

Figure S4C 14

Figure S4D 15

Figure S4E 16

Figure S4F 17

Figure S4G 18

Figure S4H 19

Figure S4I 20

Figure S4J 21

Figure S4K 22

Figure S4L 23

Figure S5: Forest plot presenting the sensitivity analysis regarding VO_{2max} performed by removing studies with a high risk of bias in 4 or more domains. 24

Figure S6A: Forest plot presenting the absolute VO_{2max} subgroup analysis based on competition level. 25

Figure S6B: Forest plot presenting the absolute VO_{2max} subgroup analysis based on CPET modality. 26

Figure S6C: Forest plot presenting the absolute VO_{2max} subgroup analysis based on sex distribution. 27

Figure S6D: Forest plot presenting the absolute VO_{2max} subgroup analysis based on comparison type. 28

Figure S6E: Forest plot presenting the absolute VO_{2max} subgroup analysis based on the presence of symptoms in athletes during COVID-19 infection. 29

Figure S6F: Forest plot presenting the absolute VO_{2max} subgroup analysis based on time elapsed from COVID-19 infection to CPET. 30

Figure S7A: Forest plot presenting the HR_{max} subgroup analysis based on competition level. 31

Figure S7B: Forest plot presenting the HR_{max} subgroup analysis based on CPET modality. 32

Figure S7C: Forest plot presenting the HR_{max} subgroup analysis based on sex distribution. 33

Figure S7D: Forest plot presenting the HR_{max} subgroup analysis based on comparison type. 34

Figure S7E: Forest plot presenting the HR_{max} subgroup analysis based on the presence of symptoms in athletes during COVID-19 infection. 35

Figure S7F: Forest plot presenting the HR_{max} subgroup analysis based on time elapsed from COVID-19 infection to CPET. 36

Figure S8A: Forest plot presenting the VE_{max} subgroup analysis based on competition level. 37

Figure S8B: Forest plot presenting the VE_{max} subgroup analysis based on CPET modality. 38

Figure S8C: Forest plot presenting the VE_{max} subgroup analysis based on sex distribution. 39

Figure S8D: Forest plot presenting the VE_{max} subgroup analysis based on comparison type. 40

Figure S8E: Forest plot presenting the VE_{max} subgroup analysis based on the presence of symptoms in athletes during COVID-19 infection. 41

Figure S8F: Forest plot presenting the VE_{max} subgroup analysis based on time elapsed from COVID-19 infection to CPET. 42

Figure S9: Funnel plot assessing the publication bias for relative VO_{2max}. 43

Figure S10: Funnel plot assessing the publication bias for absolute VO_{2max}. 44

Figure S11: Funnel plot assessing the publication bias for HR_{max}. 45

Figure S12: Funnel plot assessing the publication bias for VE_{max}. **46**

Figure S13: Risk of bias assessment of included studies. **47**

Table S3: The summary of findings in terms of the certainty of evidence regarding relative VO_{2max}, absolute VO_{2max}, HR_{max}, and VE_{max}. **48**

Supplementary references **49**

Figure S1: Forest plot comparing age between post-COVID athletes and a non-COVID group.

Study or Subgroup	MD	SE	Weight	Mean difference IV, Random, 95% CI [years]
Csulak et al.	-1.2	1.27923	11.6%	-1.20 [-3.71 , 1.31]
Moulson et al.	0	1.033487	16.6%	0.00 [-2.03 , 2.03]
Stavrou et al.	0.1	1.344805	10.6%	0.10 [-2.54 , 2.74]
Stojmenović et al.	0.3	1.52437	8.5%	0.30 [-2.69 , 3.29]
Aldossari et al.	0.81	0.862436	22.0%	0.81 [-0.88 , 2.50]
Keller et al.	1.7	0.679306	30.8%	1.70 [0.37 , 3.03]
**Total (Wald[a])**			**100.0%**	**0.60 [-0.32 , 1.51]**

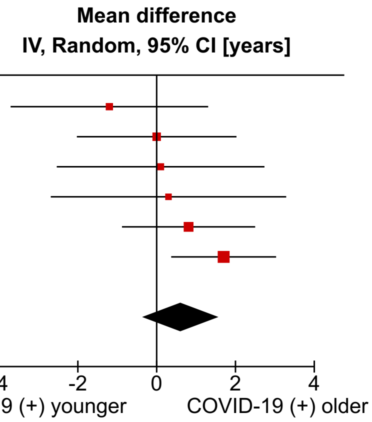

Test for overall effect: Z = 1.28 (P = 0.20)

Heterogeneity: Tau^2 (REML[b]) = 0.25; Chi^2 = 5.12, df = 5 (P = 0.40); I^2 = 19%

Footnotes

[a]CI calculated by Wald-type method.

[b]Tau^2 calculated by Restricted Maximum-Likelihood method.

Figure S2: Forest plot comparing BMI between post-COVID athletes and a non-COVID group.

Study or Subgroup	MD	SE	Weight	Mean difference IV, Random, 95% CI [kg/m2]
Moulson et al.	-0.7	0.75214	13.0%	-0.70 [-2.17 , 0.77]
Stavrou et al.	-0.7	0.496488	18.8%	-0.70 [-1.67 , 0.27]
Śliż et al.	0.01	0.525749	18.0%	0.01 [-1.02 , 1.04]
Komici et al.[a]	0.35	0.780733	12.5%	0.35 [-1.18 , 1.88]
Aldossari et al.	0.53	0.802526	12.1%	0.53 [-1.04 , 2.10]
Keller et al.[a]	1.2	0.236277	25.5%	1.20 [0.74 , 1.66]
**Total (Wald[b])**			**100.0%**	**0.19 [-0.53 , 0.91]**

Mean difference IV, Random, 95% CI [kg/m2]

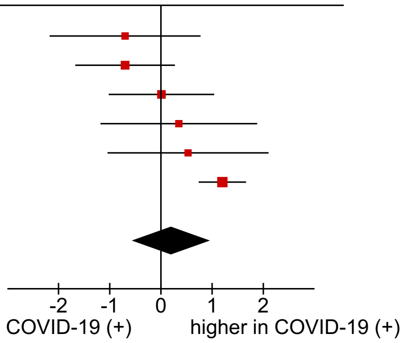

Test for overall effect: Z = 0.53 (P = 0.60)

Heterogeneity: Tau^2 (REML[c]) = 0.47; Chi^2 = 17.65, df = 5 (P = 0.003); I^2 = 64%

Footnotes

[a]Assumptions: mean = median; SD calculated by dividing the difference between the upper and lower limits of the IQR by 1.35.

[b]CI calculated by Wald-type method.

[c]Tau^2 calculated by Restricted Maximum-Likelihood method.

Figure S3A: Forest plot presenting the relative VO_{2max} subgroup analysis based on competition level.

Study or Subgroup	MD	SE	Weight	Mean difference IV, Random, 95% CI [mL/kg*min]	A	B	C	D	E	F	G	H
**3.2.1 Recreational**												
Aldossari et al.	-9.28	2.848575	4.3%	-9.28 [-14.86 , -3.70]	−	+	+	−	+	−	−	+
**Subtotal**			**4.3%**	**-9.28 [-14.86 , -3.70]**								
Test for overall effect: Z = 3.26 (P = 0.001)												
Heterogeneity: Not applicable												
**3.2.2 Competitive**												
Stojmenović et al.	-5.03	1.625557	8.7%	-5.03 [-8.22 , -1.84]	−	−	+	−	−	−	+	+
Parpa et al.	-3.01	1.514377	9.3%	-3.01 [-5.98 , -0.04]	+	−	+	+	−	−	+	+
Bruzzese et al.	-2.7	2.303259	5.8%	-2.70 [-7.21 , 1.81]	−	−	+	−	−	−	+	+
Roczniok et al.[a]	-2.42	1.840775	7.7%	-2.42 [-6.03 , 1.19]	+	−	−	−	−	−	+	+
Keller et al.[b]	-1.9	0.631563	15.1%	-1.90 [-3.14 , -0.66]	+	−	−	−	+	−	−	−
Stavrou et al.	0.3	1.423376	9.9%	0.30 [-2.49 , 3.09]	+	+	−	−	+	−	+	+
Csulak et al. (longitudinal)[c]	0.65	1.772718	8.0%	0.65 [-2.82 , 4.12]	−	+	+	+	+	+	+	+
Komici et al.[b]	1.1	1.965994	7.1%	1.10 [-2.75 , 4.95]	+	+	+	+	+	−	+	+
Csulak et al.[c]	1.3	1.488923	9.5%	1.30 [-1.62 , 4.22]	−	+	+	+	+	+	+	+
**Subtotal (Wald[d])**			**81.1%**	**-1.32 [-2.70 , 0.05]**								
Test for overall effect: Z = 1.88 (P = 0.06)												
Heterogeneity: Tau² (REML[e]) = 2.02; Chi² = 14.99, df = 8 (P = 0.06); I² = 50%												
**3.2.3 Mixed**												
Śliż et al.	-2.84	1.525196	9.3%	-2.84 [-5.83 , 0.15]	+	+	+	+	+	−	+	+
Moulson et al.	-1.8	2.477422	5.3%	-1.80 [-6.66 , 3.06]	+	+	−	+	+	+	+	+
**Subtotal (Wald[d])**			**14.6%**	**-2.55 [-5.10 , -0.01]**								
Test for overall effect: Z = 1.97 (P = 0.05)												
Heterogeneity: Tau² (REML[e]) = 0.00; Chi² = 0.13, df = 1 (P = 0.72); I² = 0%												
**Total (Wald[d])**			**100.0%**	**-1.83 [-3.16 , -0.49]**								
Test for overall effect: Z = 2.68 (P = 0.007)												
Test for subgroup differences: Chi² = 7.63, df = 2 (P = 0.02), I² = 73.8%												
Heterogeneity: Tau² (REML[e]) = 2.67; Chi² = 22.83, df = 11 (P = 0.02); I² = 54%												

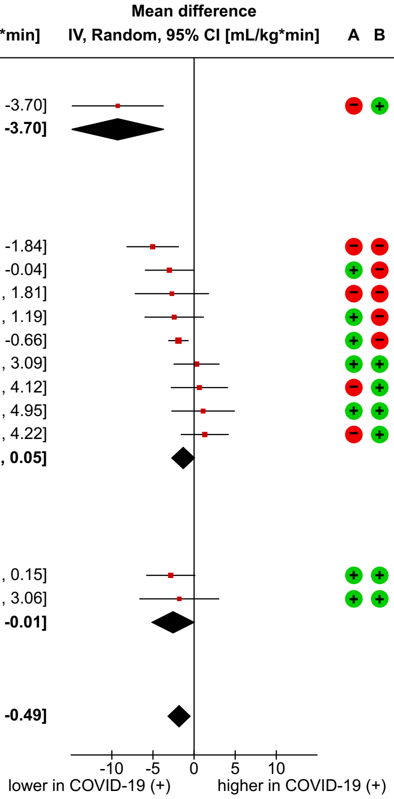

Footnotes
[a]Combined mean±SD for COVID-19(+) athletes from weighted mean and pooled sample SD using groups with and without symptoms.
[b]Assumptions: mean = median; SD calculated by dividing the difference between the upper and lower limits of the IQR by 1.35.
[c]Mean and SD calculated for the whole cohorts based on values for male and female.
[d]CI calculated by Wald-type method.
[e]Tau² calculated by Restricted Maximum-Likelihood method.

Risk of bias legend
(A) Eligibility & Sampling
(B) Population & Setting Reporting
(C) Exposure Ascertainment
(D) Outcome Definition / Diagnostic Criteria
(E) Confounders—Identification
(F) Confounders—Control/Adjustment
(G) Outcome Measurement Validity/Reliability
(H) Statistical Methods Appropriateness

Note: A red circle means high risk of bias, while a green circle means low risk of bias.

Figure S3B: Forest plot presenting the relative VO_{2max} subgroup analysis based on CPET modality.

Study or Subgroup	MD	SE	Weight	Mean difference IV, Random, 95% CI [mL/kg*min]	A	B	C	D	E	F	G	H
**3.3.1 Cycle ergometer**												
Roczniok et al.[a]	-2.42	1.840775	7.7%	-2.42 [-6.03 , 1.19]	+	−	−	−	−	−	+	+
**Subtotal**			**7.7%**	**-2.42 [-6.03 , 1.19]**								
Test for overall effect: Z = 1.31 (P = 0.19)												
Heterogeneity: Not applicable												
**3.3.2 Mixed**												
Śliż et al.	-2.84	1.525196	9.3%	-2.84 [-5.83 , 0.15]	+	+	+	+	+	−	+	+
Keller et al.[b]	-1.9	0.631563	15.1%	-1.90 [-3.14 , -0.66]	+	−	−	−	+	−	−	−
Moulson et al.	-1.8	2.477422	5.3%	-1.80 [-6.66 , 3.06]	+	+	−	+	+	+	+	+
**Subtotal (Wald[c])**			**29.7%**	**-2.03 [-3.14 , -0.91]**								
Test for overall effect: Z = 3.57 (P = 0.0004)												
Heterogeneity: Tau² (REML[d]) = 0.00; Chi² = 0.33, df = 2 (P = 0.85); I² = 0%												
**3.3.3 Treadmill**												
Aldossari et al.	-9.28	2.848575	4.3%	-9.28 [-14.86 , -3.70]	−	+	+	−	+	−	−	+
Stojmenović et al.	-5.03	1.625557	8.7%	-5.03 [-8.22 , -1.84]	−	−	+	−	−	−	+	+
Parpa et al.	-3.01	1.514377	9.3%	-3.01 [-5.98 , -0.04]	+	−	+	+	−	−	+	+
Bruzzese et al.	-2.7	2.303259	5.8%	-2.70 [-7.21 , 1.81]	−	−	+	−	−	−	+	+
Stavrou et al.	0.3	1.423376	9.9%	0.30 [-2.49 , 3.09]	+	+	−	−	+	−	+	+
Csulak et al. (longitudinal)[e]	0.65	1.772718	8.0%	0.65 [-2.82 , 4.12]	−	+	+	+	+	+	+	+
Komici et al.[b]	1.1	1.965994	7.1%	1.10 [-2.75 , 4.95]	+	+	+	+	+	−	+	+
Csulak et al.[e]	1.3	1.488923	9.5%	1.30 [-1.62 , 4.22]	−	+	+	+	+	+	+	+
**Subtotal (Wald[c])**			**62.7%**	**-1.74 [-3.99 , 0.50]**								
Test for overall effect: Z = 1.52 (P = 0.13)												
Heterogeneity: Tau² (REML[d]) = 7.07; Chi² = 21.76, df = 7 (P = 0.003); I² = 70%												
**Total (Wald[c])**			**100.0%**	**-1.83 [-3.16 , -0.49]**								

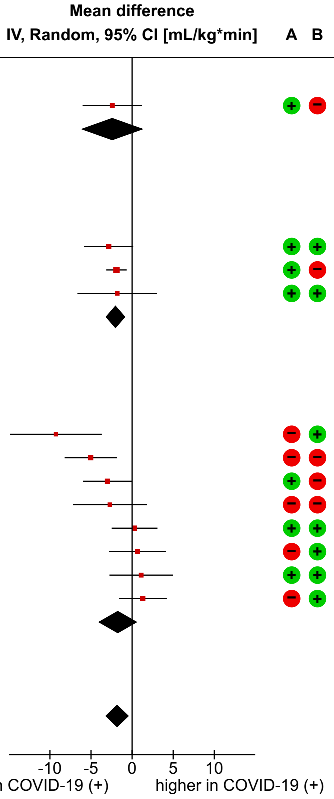

Test for overall effect: Z = 2.68 (P = 0.007)
Test for subgroup differences: Chi² = 0.10, df = 2 (P = 0.95), I² = 0%
Heterogeneity: Tau² (REML[d]) = 2.67; Chi² = 22.83, df = 11 (P = 0.02); I² = 54%

Footnotes

[a]Combined mean±SD for COVID-19(+) athletes from weighted mean and pooled sample SD using groups with and without symptoms.
[b]Assumptions: mean = median; SD calculated by dividing the difference between the upper and lower limits of the IQR by 1.35.
[c]CI calculated by Wald-type method.
[d]Tau² calculated by Restricted Maximum-Likelihood method.
[e]Mean and SD calculated for the whole cohorts based on values for male and female.

Risk of bias legend

(A) Eligibility & Sampling
(B) Population & Setting Reporting
(C) Exposure Ascertainment
(D) Outcome Definition / Diagnostic Criteria
(E) Confounders—Identification
(F) Confounders—Control/Adjustment
(G) Outcome Measurement Validity/Reliability
(H) Statistical Methods Appropriateness

Note: A red circle means high risk of bias, while a green circle means low risk of bias.

Figure S3C: Forest plot presenting the relative VO_{2max} subgroup analysis based on sex distribution.

Study or Subgroup	MD	SE	Weight	Mean difference IV, Random, 95% CI [mL/kg*min]	A	B	C	D	E	F	G	H
**3.4.1 Mixed**												
Aldossari et al.	-9.28	2.848575	4.3%	-9.28 [-14.86 , -3.70]	−	+	+	−	+	−	−	+
Śliż et al.	-2.84	1.525196	9.3%	-2.84 [-5.83 , 0.15]	+	+	+	+	+	−	+	+
Bruzzese et al.	-2.7	2.303259	5.8%	-2.70 [-7.21 , 1.81]	−	−	+	−	−	−	+	+
Keller et al.[a]	-1.9	0.631563	15.1%	-1.90 [-3.14 , -0.66]	+	−	−	−	+	−	−	−
Moulson et al.	-1.8	2.477422	5.3%	-1.80 [-6.66 , 3.06]	+	+	+	+	+	+	+	+
Csulak et al. (longitudinal)[b]	0.65	1.772718	8.0%	0.65 [-2.82 , 4.12]	−	+	+	+	+	+	+	+
Csulak et al.[b]	1.3	1.488923	9.5%	1.30 [-1.62 , 4.22]	−	+	+	+	+	+	+	+
**Subtotal (Wald[c])**			**57.3%**	**-1.84 [-3.76 , 0.08]**								
Test for overall effect: Z = 1.88 (P = 0.06)												
Heterogeneity: Tau² (REML[d]) = 3.55; Chi² = 13.75, df = 6 (P = 0.03); I² = 60%												
**3.4.2 Not reported**												
Parpa et al.	-3.01	1.514377	9.3%	-3.01 [-5.98 , -0.04]	+	−	+	+	−	−	+	+
Roczniok et al.[e]	-2.42	1.840775	7.7%	-2.42 [-6.03 , 1.19]	+	−	−	−	−	−	+	+
Komici et al.[a]	1.1	1.965994	7.1%	1.10 [-2.75 , 4.95]	+	+	+	+	+	−	+	+
**Subtotal (Wald[c])**			**24.1%**	**-1.66 [-4.04 , 0.71]**								
Test for overall effect: Z = 1.37 (P = 0.17)												
Heterogeneity: Tau² (REML[d]) = 1.32; Chi² = 2.93, df = 2 (P = 0.23); I² = 30%												
**3.4.3 Only male**												
Stojmenović et al.	-5.03	1.625557	8.7%	-5.03 [-8.22 , -1.84]	−	−	+	−	−	−	+	+
Stavrou et al.	0.3	1.423376	9.9%	0.30 [-2.49 , 3.09]	+	+	−	−	+	−	+	+
**Subtotal (Wald[c])**			**18.6%**	**-2.31 [-7.53 , 2.91]**								
Test for overall effect: Z = 0.87 (P = 0.39)												
Heterogeneity: Tau² (REML[d]) = 11.87; Chi² = 6.09, df = 1 (P = 0.01); I² = 84%												
**Total (Wald[c])**			**100.0%**	**-1.83 [-3.16 , -0.49]**								

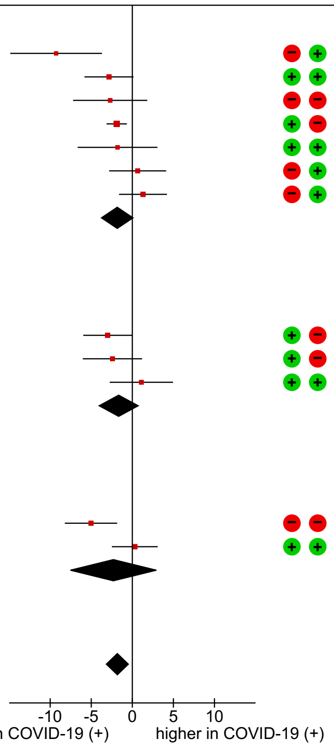

Test for overall effect: Z = 2.68 (P = 0.007)
Test for subgroup differences: Chi² = 0.05, df = 2 (P = 0.98), I² = 0%
Heterogeneity: Tau² (REML[d]) = 2.67; Chi² = 22.83, df = 11 (P = 0.02); I² = 54%

Footnotes
[a]Assumptions: mean = median; SD calculated by dividing the difference between the upper and lower limits of the IQR by 1.35.
[b]Mean and SD calculated for the whole cohorts based on values for male and female.
[c]CI calculated by Wald-type method.
[d]Tau² calculated by Restricted Maximum-Likelihood method.
[e]Combined mean±SD for COVID-19(+) athletes from weighted mean and pooled sample SD using groups with and without symptoms.

Risk of bias legend
(A) Eligibility & Sampling
(B) Population & Setting Reporting
(C) Exposure Ascertainment
(D) Outcome Definition / Diagnostic Criteria
(E) Confounders—Identification
(F) Confounders—Control/Adjustment
(G) Outcome Measurement Validity/Reliability
(H) Statistical Methods Appropriateness

Note: A red circle means high risk of bias, while a green circle means low risk of bias.

Figure S3D: Forest plot presenting the relative VO_{2max} subgroup analysis based on comparison type.

Study or Subgroup	MD	SE	Weight	Mean difference IV, Random, 95% CI [mL/kg*min]	A	B	C	D	E	F	G	H
**3.5.1 Post-COVID athletes vs. healthy reference**												
Aldossari et al.	-9.28	2.848575	4.3%	-9.28 [-14.86 , -3.70]	−	+	+	−	+	−	−	+
Roczniok et al.[a]	-2.42	1.840775	7.7%	-2.42 [-6.03 , 1.19]	+	−	−	−	−	−	+	+
Keller et al.[b]	-1.9	0.631563	15.1%	-1.90 [-3.14 , -0.66]	+	−	−	−	+	−	−	−
Moulson et al.	-1.8	2.477422	5.3%	-1.80 [-6.66 , 3.06]	+	+	−	+	+	+	+	+
Stavrou et al.	0.3	1.423376	9.9%	0.30 [-2.49 , 3.09]	+	+	−	−	+	−	+	+
Komici et al.[b]	1.1	1.965994	7.1%	1.10 [-2.75 , 4.95]	+	+	+	+	+	−	+	+
Csulak et al.[c]	1.3	1.488923	9.5%	1.30 [-1.62 , 4.22]	−	+	+	+	+	+	+	+
**Subtotal (Wald[d])**			**58.8%**	**-1.29 [-3.20 , 0.61]**								
Test for overall effect: Z = 1.33 (P = 0.18)												
Heterogeneity: Tau^2 (REML[e]) = 3.63; Chi^2 = 14.94, df = 6 (P = 0.02); I^2 = 62%												
**3.5.2 Post-COVID athletes vs. the same athletes before infection**												
Stojmenović et al.	-5.03	1.625557	8.7%	-5.03 [-8.22 , -1.84]	−	−	+	−	−	−	+	+
Parpa et al.	-3.01	1.514377	9.3%	-3.01 [-5.98 , -0.04]	+	−	+	+	−	−	+	+
Śliż et al.	-2.84	1.525196	9.3%	-2.84 [-5.83 , 0.15]	+	+	+	+	+	−	+	+
Bruzzese et al.	-2.7	2.303259	5.8%	-2.70 [-7.21 , 1.81]	−	−	+	−	−	−	+	+
Csulak et al. (longitudinal)[c]	0.65	1.772718	8.0%	0.65 [-2.82 , 4.12]	−	+	+	+	+	+	+	+
**Subtotal (Wald[d])**			**41.2%**	**-2.67 [-4.46 , -0.88]**								
Test for overall effect: Z = 2.92 (P = 0.003)												
Heterogeneity: Tau^2 (REML[e]) = 1.25; Chi^2 = 5.68, df = 4 (P = 0.22); I^2 = 30%												
**Total (Wald[d])**			**100.0%**	**-1.83 [-3.16 , -0.49]**								
Test for overall effect: Z = 2.68 (P = 0.007)												
Test for subgroup differences: Chi^2 = 1.07, df = 1 (P = 0.30), I^2 = 6.2%												
Heterogeneity: Tau^2 (REML[e]) = 2.67; Chi^2 = 22.83, df = 11 (P = 0.02); I^2 = 54%												

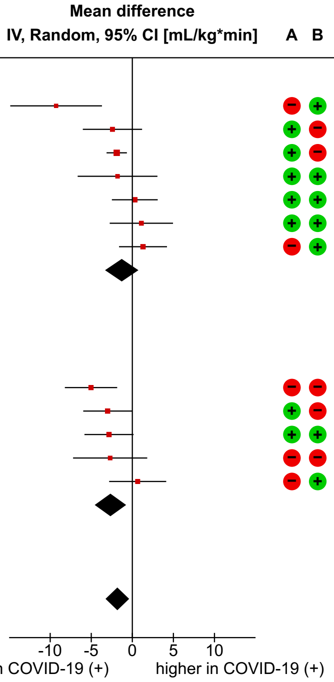

Footnotes

[a]Combined mean±SD for COVID-19(+) athletes from weighted mean and pooled sample SD using groups with and without symptoms.
[b]Assumptions: mean = median; SD calculated by dividing the difference between the upper and lower limits of the IQR by 1.35.
[c]Mean and SD calculated for the whole cohorts based on values for male and female.
[d]CI calculated by Wald-type method.
[e]Tau^2 calculated by Restricted Maximum-Likelihood method.

Risk of bias legend

(A) Eligibility & Sampling
(B) Population & Setting Reporting
(C) Exposure Ascertainment
(D) Outcome Definition / Diagnostic Criteria
(E) Confounders—Identification
(F) Confounders—Control/Adjustment
(G) Outcome Measurement Validity/Reliability
(H) Statistical Methods Appropriateness

Note: A red circle means high risk of bias, while a green circle means low risk of bias.

Figure S3E: Forest plot presenting the relative VO_{2max} subgroup analysis based on the presence of symptoms in athletes during COVID-19 infection.

Study or Subgroup	MD	SE	Weight	Mean difference IV, Random, 95% CI [mL/kg*min]	A	B	C	D	E	F	G	H
**3.6.1 Mixed**												
Bruzzese et al.	-2.7	2.303259	5.8%	-2.70 [-7.21 , 1.81]	−	−	+	−	−	−	+	+
Roczniok et al.[a]	-2.42	1.840775	7.7%	-2.42 [-6.03 , 1.19]	+	−	−	−	−	−	+	+
Keller et al.[b]	-1.9	0.631563	15.1%	-1.90 [-3.14 , -0.66]	+	−	−	−	+	−	−	−
Csulak et al. (longitudinal)[c]	0.65	1.772718	8.0%	0.65 [-2.82 , 4.12]	−	+	+	+	+	+	+	+
Komici et al.[b]	1.1	1.965994	7.1%	1.10 [-2.75 , 4.95]	+	+	+	+	+	−	+	+
Csulak et al.[c]	1.3	1.488923	9.5%	1.30 [-1.62 , 4.22]	−	+	+	+	+	+	+	+
**Subtotal (Wald[d])**			**53.2%**	**-0.81 [-2.29 , 0.66]**								
Test for overall effect: Z = 1.08 (P = 0.28)												
Heterogeneity: Tau² (REML[e]) = 1.14; Chi² = 7.37, df = 5 (P = 0.19); I² = 35%												
**3.6.2 Only symptomatic**												
Parpa et al.	-3.01	1.514377	9.3%	-3.01 [-5.98 , -0.04]	+	−	+	+	−	−	+	+
Moulson et al.	-1.8	2.477422	5.3%	-1.80 [-6.66 , 3.06]	+	+	−	+	+	+	+	+
**Subtotal (Wald[d])**			**14.6%**	**-2.68 [-5.21 , -0.15]**								
Test for overall effect: Z = 2.07 (P = 0.04)												
Heterogeneity: Tau² (REML[e]) = 0.00; Chi² = 0.17, df = 1 (P = 0.68); I² = 0%												
**3.6.3 Not reported**												
Aldossari et al.	-9.28	2.848575	4.3%	-9.28 [-14.86 , -3.70]	−	+	+	−	+	−	−	+
Stojmenović et al.	-5.03	1.625557	8.7%	-5.03 [-8.22 , -1.84]	−	−	+	−	−	−	+	+
Śliż et al.	-2.84	1.525196	9.3%	-2.84 [-5.83 , 0.15]	+	+	+	+	+	−	+	+
Stavrou et al.	0.3	1.423376	9.9%	0.30 [-2.49 , 3.09]	+	+	−	−	+	−	+	+
**Subtotal (Wald[d])**			**32.2%**	**-3.74 [-7.32 , -0.15]**								
Test for overall effect: Z = 2.04 (P = 0.04)												
Heterogeneity: Tau² (REML[e]) = 9.99; Chi² = 11.79, df = 3 (P = 0.008); I² = 77%												
**Total (Wald[d])**			**100.0%**	**-1.83 [-3.16 , -0.49]**								

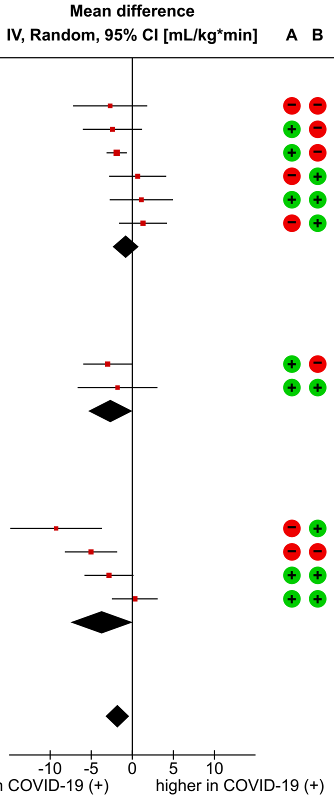

Test for overall effect: Z = 2.68 (P = 0.007)
Test for subgroup differences: Chi² = 3.15, df = 2 (P = 0.21), I² = 36.4%
Heterogeneity: Tau² (REML[e]) = 2.67; Chi² = 22.83, df = 11 (P = 0.02); I² = 54%

Footnotes
[a]Combined mean±SD for COVID-19(+) athletes from weighted mean and pooled sample SD using groups with and without symptoms.
[b]Assumptions: mean = median; SD calculated by dividing the difference between the upper and lower limits of the IQR by 1.35.
[c]Mean and SD calculated for the whole cohorts based on values for male and female.
[d]CI calculated by Wald-type method.
[e]Tau² calculated by Restricted Maximum-Likelihood method.

Risk of bias legend
(A) Eligibility & Sampling
(B) Population & Setting Reporting
(C) Exposure Ascertainment
(D) Outcome Definition / Diagnostic Criteria
(E) Confounders—Identification
(F) Confounders—Control/Adjustment
(G) Outcome Measurement Validity/Reliability
(H) Statistical Methods Appropriateness

Note: A red circle means high risk of bias, while a green circle means low risk of bias.

Figure S3F: Forest plot presenting the relative VO_{2max} subgroup analysis based on time elapsed from COVID-19 infection to CPET.

Study or Subgroup	MD	SE	Weight	Mean difference IV, Random, 95% CI [mL/kg*min]
**3.7.1 Not reported**				
Keller et al.[a]	-1.9	0.631563	15.1%	-1.90 [-3.14 , -0.66]
**Subtotal**			**15.1%**	**-1.90 [-3.14 , -0.66]**
Test for overall effect: Z = 3.01 (P = 0.003)				
Heterogeneity: Not applicable				
**3.7.2 Up to 1 month after infection**				
Stojmenović et al.	-5.03	1.625557	8.7%	-5.03 [-8.22 , -1.84]
Bruzzese et al.	-2.7	2.303259	5.8%	-2.70 [-7.21 , 1.81]
Stavrou et al.	0.3	1.423376	9.9%	0.30 [-2.49 , 3.09]
Csulak et al. (longitudinal)[b]	0.65	1.772718	8.0%	0.65 [-2.82 , 4.12]
Komici et al.[a]	1.1	1.965994	7.1%	1.10 [-2.75 , 4.95]
Csulak et al.[b]	1.3	1.488923	9.5%	1.30 [-1.62 , 4.22]
**Subtotal (Wald[c])**			**49.0%**	**-0.65 [-2.75 , 1.44]**
Test for overall effect: Z = 0.61 (P = 0.54)				
Heterogeneity: Tau² (REML[d]) = 3.83; Chi² = 11.53, df = 5 (P = 0.04); I² = 57%				
**3.7.3 More than 1 month after infection**				
Aldossari et al.	-9.28	2.848575	4.3%	-9.28 [-14.86 , -3.70]
Parpa et al.	-3.01	1.514377	9.3%	-3.01 [-5.98 , -0.04]
Śliż et al.	-2.84	1.525196	9.3%	-2.84 [-5.83 , 0.15]
Roczniok et al.[e]	-2.42	1.840775	7.7%	-2.42 [-6.03 , 1.19]
Moulson et al.	-1.8	2.477422	5.3%	-1.80 [-6.66 , 3.06]
**Subtotal (Wald[c])**			**35.9%**	**-3.24 [-4.87 , -1.61]**
Test for overall effect: Z = 3.89 (P < 0.0001)				
Heterogeneity: Tau² (REML[d]) = 0.00; Chi² = 5.12, df = 4 (P = 0.27); I² = 0%				
**Total (Wald[c])**			**100.0%**	**-1.83 [-3.16 , -0.49]**

Mean difference IV, Random, 95% CI [mL/kg*min] — Risk of Bias A B C D E F G H

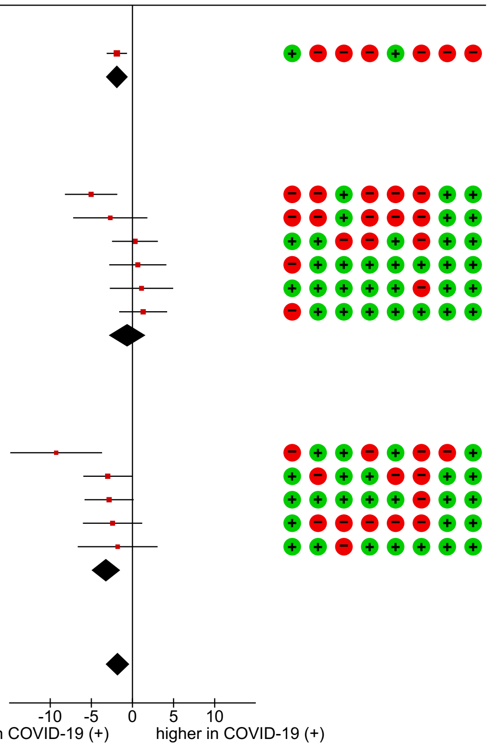

Test for overall effect: Z = 2.68 (P = 0.007)
Test for subgroup differences: Chi² = 3.80, df = 2 (P = 0.15), I² = 47.3%
Heterogeneity: Tau² (REML[d]) = 2.67; Chi² = 22.83, df = 11 (P = 0.02); I² = 54%

Footnotes
[a]Assumptions: mean = median; SD calculated by dividing the difference between the upper and lower limits of the IQR by 1.35.
[b]Mean and SD calculated for the whole cohorts based on values for male and female.
[c]CI calculated by Wald-type method.
[d]Tau² calculated by Restricted Maximum-Likelihood method.
[e]Combined mean±SD for COVID-19(+) athletes from weighted mean and pooled sample SD using groups with and without symptoms.

Risk of bias legend
(A) Eligibility & Sampling
(B) Population & Setting Reporting
(C) Exposure Ascertainment
(D) Outcome Definition / Diagnostic Criteria
(E) Confounders—Identification
(F) Confounders—Control/Adjustment
(G) Outcome Measurement Validity/Reliability
(H) Statistical Methods Appropriateness

Note: A red circle means high risk of bias, while a green circle means low risk of bias.

Figures S4A to S4L: Forest plots presenting the sensitivity analyses regarding relative VO_{2max} performed by removing every single study consecutively.

Figure S4A

Study or Subgroup	MD	SE	Weight	Mean difference IV, Random, 95% CI [mL/kg*min]	A	B	C	D	E	F	G	H
✗ Aldossari et al.	-9.28	2.848575	0.0%	-9.28 [-14.86 , -3.70]	−	+	+	−	+	−	−	+
✓ Stojmenović et al.	-5.03	1.625557	8.8%	-5.03 [-8.22 , -1.84]	−	−	+	−	−	−	+	+
✓ Parpa et al.	-3.01	1.514377	9.6%	-3.01 [-5.98 , -0.04]	+	−	+	+	−	−	+	+
✓ Śliż et al.	-2.84	1.525196	9.6%	-2.84 [-5.83 , 0.15]	+	+	+	+	+	−	+	+
✓ Bruzzese et al.	-2.7	2.303259	5.3%	-2.70 [-7.21 , 1.81]	−	−	+	−	−	−	+	+
✓ Roczniok et al.[a]	-2.42	1.840775	7.4%	-2.42 [-6.03 , 1.19]	+	−	−	−	−	−	+	+
✓ Keller et al.[b]	-1.9	0.631563	19.7%	-1.90 [-3.14 , -0.66]	+	−	−	−	+	−	−	−
✓ Moulson et al.	-1.8	2.477422	4.7%	-1.80 [-6.66 , 3.06]	+	+	−	+	+	+	+	+
✓ Stavrou et al.	0.3	1.423376	10.4%	0.30 [-2.49 , 3.09]	+	+	−	−	+	−	+	+
✓ Csulak et al. (longitudinal)[c]	0.65	1.772718	7.8%	0.65 [-2.82 , 4.12]	−	+	+	+	+	+	+	+
✓ Komici et al.[b]	1.1	1.965994	6.8%	1.10 [-2.75 , 4.95]	+	+	+	+	+	−	+	+
✓ Csulak et al.[c]	1.3	1.488923	9.8%	1.30 [-1.62 , 4.22]	−	+	+	+	+	+	+	+
**Total (Wald[d])**			**100.0%**	**-1.50 [-2.67 , -0.33]**								

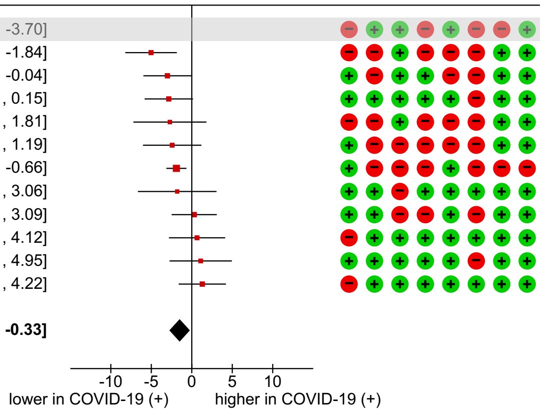

Test for overall effect: Z = 2.51 (P = 0.01)

Heterogeneity: Tau^2 (REML[e]) = 1.42; Chi^2 = 15.71, df = 10 (P = 0.11); I^2 = 40%

Footnotes

[a]Combined mean±SD for COVID-19(+) athletes from weighted mean and pooled sample SD using groups with and without symptoms.
[b]Assumptions: mean = median; SD calculated by dividing the difference between the upper and lower limits of the IQR by 1.35.
[c]Mean and SD calculated for the whole cohorts based on values for male and female.
[d]CI calculated by Wald-type method.
[e]Tau^2 calculated by Restricted Maximum-Likelihood method.

Risk of bias legend

(A) Eligibility & Sampling
(B) Population & Setting Reporting
(C) Exposure Ascertainment
(D) Outcome Definition / Diagnostic Criteria
(E) Confounders—Identification
(F) Confounders—Control/Adjustment
(G) Outcome Measurement Validity/Reliability
(H) Statistical Methods Appropriateness

Note: A red circle means high risk of bias, while a green circle means low risk of bias.

Figure S4B

Study or Subgroup	MD	SE	Weight	Mean difference IV, Random, 95% CI [mL/kg*min]	Mean difference IV, Random, 95% CI [mL/kg*min]	Risk of Bias A	B	C	D	E	F	G	H
✓ Aldossari et al.	-9.28	2.848575	4.0%	-9.28 [-14.86 , -3.70]		−	+	+	−	+	−	−	+
✕ Stojmenović et al.	-5.03	1.625557	0.0%	-5.03 [-8.22 , -1.84]		−	−	+	−	−	−	+	+
✓ Parpa et al.	-3.01	1.514377	10.2%	-3.01 [-5.98 , -0.04]		+	−	+	+	−	−	+	+
✓ Śliż et al.	-2.84	1.525196	10.1%	-2.84 [-5.83 , 0.15]		+	+	+	+	+	−	+	+
✓ Bruzzese et al.	-2.7	2.303259	5.7%	-2.70 [-7.21 , 1.81]		−	−	+	−	−	−	+	+
✓ Roczniok et al.[a]	-2.42	1.840775	7.9%	-2.42 [-6.03 , 1.19]		+	−	−	−	−	−	+	+
✓ Keller et al.[b]	-1.9	0.631563	20.1%	-1.90 [-3.14 , -0.66]		+	−	−	−	+	−	−	−
✓ Moulson et al.	-1.8	2.477422	5.1%	-1.80 [-6.66 , 3.06]		+	+	−	+	+	+	+	+
✓ Stavrou et al.	0.3	1.423376	10.9%	0.30 [-2.49 , 3.09]		+	+	−	−	+	−	+	+
✓ Csulak et al. (longitudinal)[c]	0.65	1.772718	8.3%	0.65 [-2.82 , 4.12]		−	+	+	+	+	+	+	+
✓ Komici et al.[b]	1.1	1.965994	7.2%	1.10 [-2.75 , 4.95]		+	+	+	+	+	−	+	+
✓ Csulak et al.[c]	1.3	1.488923	10.4%	1.30 [-1.62 , 4.22]		−	+	+	+	+	+	+	+
**Total (Wald[d])**			**100.0%**	**-1.49 [-2.71 , -0.26]**									

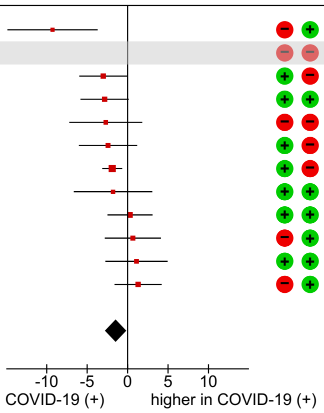

Test for overall effect: Z = 2.38 (P = 0.02)

Heterogeneity: Tau² (REML[e]) = 1.55; Chi² = 18.51, df = 10 (P = 0.05); I² = 40%

Footnotes

[a]Combined mean±SD for COVID-19(+) athletes from weighted mean and pooled sample SD using groups with and without symptoms.

[b]Assumptions: mean = median; SD calculated by dividing the difference between the upper and lower limits of the IQR by 1.35.

[c]Mean and SD calculated for the whole cohorts based on values for male and female.

[d]CI calculated by Wald-type method.

[e]Tau² calculated by Restricted Maximum-Likelihood method.

Risk of bias legend

(A) Eligibility & Sampling
(B) Population & Setting Reporting
(C) Exposure Ascertainment
(D) Outcome Definition / Diagnostic Criteria
(E) Confounders—Identification
(F) Confounders—Control/Adjustment
(G) Outcome Measurement Validity/Reliability
(H) Statistical Methods Appropriateness

Note: A red circle means high risk of bias, while a green circle means low risk of bias.

Figure S4C

Study or Subgroup	MD	SE	Weight	Mean difference IV, Random, 95% CI [mL/kg*min]	A	B	C	D	E	F	G	H
✓ Aldossari et al.	-9.28	2.848575	5.0%	-9.28 [-14.86 , -3.70]	−	+	+	−	+	−	−	+
✓ Stojmenović et al.	-5.03	1.625557	9.7%	-5.03 [-8.22 , -1.84]	−	−	+	−	−	−	+	+
✗ Parpa et al.	-3.01	1.514377	0.0%	-3.01 [-5.98 , -0.04]	+	−	+	+	−	−	+	+
✓ Śliż et al.	-2.84	1.525196	10.2%	-2.84 [-5.83 , 0.15]	+	+	+	+	+	−	+	+
✓ Bruzzese et al.	-2.7	2.303259	6.6%	-2.70 [-7.21 , 1.81]	−	−	+	−	−	−	+	+
✓ Roczniok et al.[a]	-2.42	1.840775	8.6%	-2.42 [-6.03 , 1.19]	+	−	−	−	−	−	+	+
✓ Keller et al.[b]	-1.9	0.631563	15.7%	-1.90 [-3.14 , -0.66]	+	−	−	−	+	−		−
✓ Moulson et al.	-1.8	2.477422	6.1%	-1.80 [-6.66 , 3.06]	+	+	−	+	+	+	+	+
✓ Stavrou et al.	0.3	1.423376	10.8%	0.30 [-2.49 , 3.09]	+	+	−	−	+	−	+	+
✓ Csulak et al. (longitudinal)[c]	0.65	1.772718	8.9%	0.65 [-2.82 , 4.12]	−	+	+	+	+	+	+	+
✓ Komici et al.[b]	1.1	1.965994	8.0%	1.10 [-2.75 , 4.95]	+	+	+	+	+	−	+	+
✓ Csulak et al.[c]	1.3	1.488923	10.4%	1.30 [-1.62 , 4.22]	−	+	+	+	+	+	+	+
**Total (Wald[d])**			**100.0%**	**-1.72 [-3.19 , -0.25]**								

Mean difference IV, Random, 95% CI [mL/kg*min]

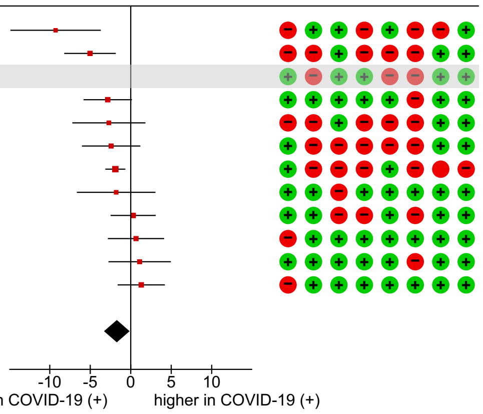

Test for overall effect: Z = 2.29 (P = 0.02)

Heterogeneity: Tau² (REML[e]) = 3.20; Chi² = 22.09, df = 10 (P = 0.01); I² = 58%

Footnotes

[a]Combined mean±SD for COVID-19(+) athletes from weighted mean and pooled sample SD using groups with and without symptoms.
[b]Assumptions: mean = median; SD calculated by dividing the difference between the upper and lower limits of the IQR by 1.35.
[c]Mean and SD calculated for the whole cohorts based on values for male and female.
[d]CI calculated by Wald-type method.
[e]Tau² calculated by Restricted Maximum-Likelihood method.

Risk of bias legend

(A) Eligibility & Sampling
(B) Population & Setting Reporting
(C) Exposure Ascertainment
(D) Outcome Definition / Diagnostic Criteria
(E) Confounders—Identification
(F) Confounders—Control/Adjustment
(G) Outcome Measurement Validity/Reliability
(H) Statistical Methods Appropriateness

Note: A red circle means high risk of bias, while a green circle means low risk of bias.

Figure S4D

Study or Subgroup	MD	SE	Weight	Mean difference IV, Random, 95% CI [mL/kg*min]	A	B	C	D	E	F	G	H
✓ Aldossari et al.	-9.28	2.848575	5.0%	-9.28 [-14.86 , -3.70]	−	+	+	−	+	−	−	+
✓ Stojmenović et al.	-5.03	1.625557	9.7%	-5.03 [-8.22 , -1.84]	−	−	+	−	−	−	+	+
✓ Parpa et al.	-3.01	1.514377	10.3%	-3.01 [-5.98 , -0.04]	+	−	+	+	−	−	+	+
✗ Śliż et al.	-2.84	1.525196	0.0%	-2.84 [-5.83 , 0.15]	+	+	+	+	+	−	+	+
✓ Bruzzese et al.	-2.7	2.303259	6.7%	-2.70 [-7.21 , 1.81]	−	−	+	−	−	−	+	+
✓ Roczniok et al.[a]	-2.42	1.840775	8.6%	-2.42 [-6.03 , 1.19]	+	−	−	−	−	−	+	+
✓ Keller et al.[b]	-1.9	0.631563	15.6%	-1.90 [-3.14 , -0.66]	+	−	−	−	+	−	−	−
✓ Moulson et al.	-1.8	2.477422	6.1%	-1.80 [-6.66 , 3.06]	+	+	−	+	+	+	+	+
✓ Stavrou et al.	0.3	1.423376	10.8%	0.30 [-2.49 , 3.09]	+	+	−	−	+	−	+	+
✓ Csulak et al. (longitudinal)[c]	0.65	1.772718	8.9%	0.65 [-2.82 , 4.12]	−	+	+	+	+	+	+	+
✓ Komici et al.[b]	1.1	1.965994	8.0%	1.10 [-2.75 , 4.95]	+	+	+	+	+	−	+	+
✓ Csulak et al.[c]	1.3	1.488923	10.4%	1.30 [-1.62 , 4.22]	−	+	+	+	+	+	+	+
**Total (Wald[d])**			**100.0%**	**-1.74 [-3.22 , -0.26]**								

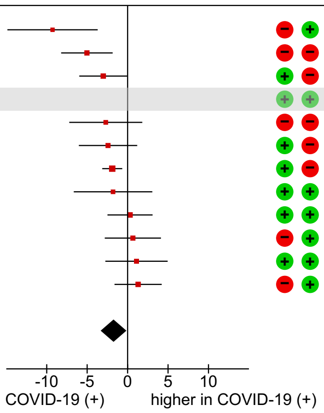

Test for overall effect: Z = 2.30 (P = 0.02)

Heterogeneity: Tau² (REML[e]) = 3.28; Chi² = 22.29, df = 10 (P = 0.01); I² = 58%

Footnotes

[a]Combined mean±SD for COVID-19(+) athletes from weighted mean and pooled sample SD using groups with and without symptoms.

[b]Assumptions: mean = median; SD calculated by dividing the difference between the upper and lower limits of the IQR by 1.35.

[c]Mean and SD calculated for the whole cohorts based on values for male and female.

[d]CI calculated by Wald-type method.

[e]Tau² calculated by Restricted Maximum-Likelihood method.

Risk of bias legend

(A) Eligibility & Sampling
(B) Population & Setting Reporting
(C) Exposure Ascertainment
(D) Outcome Definition / Diagnostic Criteria
(E) Confounders—Identification
(F) Confounders—Control/Adjustment
(G) Outcome Measurement Validity/Reliability
(H) Statistical Methods Appropriateness

Note: A red circle means high risk of bias, while a green circle means low risk of bias.

Figure S4E

Study or Subgroup	MD	SE	Weight	Mean difference IV, Random, 95% CI [mL/kg*min]	A	B	C	D	E	F	G	H
✓ Aldossari et al.	-9.28	2.848575	4.8%	-9.28 [-14.86 , -3.70]	−	+	+	−	+	−	−	+
✓ Stojmenović et al.	-5.03	1.625557	9.3%	-5.03 [-8.22 , -1.84]	−	−	+	−	−	−	+	+
✓ Parpa et al.	-3.01	1.514377	9.9%	-3.01 [-5.98 , -0.04]	+	−	+	+	−	−	+	+
✓ Śliż et al.	-2.84	1.525196	9.9%	-2.84 [-5.83 , 0.15]	+	+	+	+	+	−	+	+
✕ Bruzzese et al.	-2.7	2.303259	0.0%	-2.70 [-7.21 , 1.81]	−	−	+	−	−	−	+	+
✓ Roczniok et al.[a]	-2.42	1.840775	8.3%	-2.42 [-6.03 , 1.19]	+	−	−	−	−	−	+	+
✓ Keller et al.[b]	-1.9	0.631563	15.2%	-1.90 [-3.14 , -0.66]	+	−	−	−	+	−	−	−
✓ Moulson et al.	-1.8	2.477422	5.8%	-1.80 [-6.66 , 3.06]	+	+	−	+	+	+	+	+
✓ Stavrou et al.	0.3	1.423376	10.4%	0.30 [-2.49 , 3.09]	+	+	−	−	+	−	+	+
✓ Csulak et al. (longitudinal)[c]	0.65	1.772718	8.6%	0.65 [-2.82 , 4.12]	−	+	+	+	+	+	+	+
✓ Komici et al.[b]	1.1	1.965994	7.7%	1.10 [-2.75 , 4.95]	+	+	+	+	+	−	+	+
✓ Csulak et al.[c]	1.3	1.488923	10.1%	1.30 [-1.62 , 4.22]	−	+	+	+	+	+	+	+
**Total (Wald[d])**			**100.0%**	**-1.78 [-3.22 , -0.35]**								

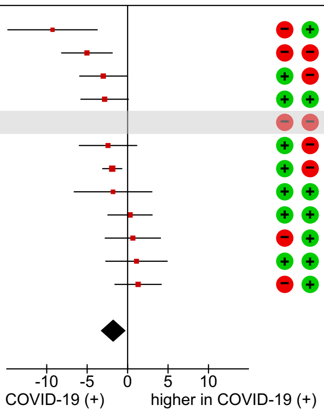

Test for overall effect: Z = 2.43 (P = 0.02)

Heterogeneity: Tau² (REML[e]) = 3.14; Chi² = 22.65, df = 10 (P = 0.01); I² = 59%

Footnotes

[a]Combined mean±SD for COVID-19(+) athletes from weighted mean and pooled sample SD using groups with and without symptoms.
[b]Assumptions: mean = median; SD calculated by dividing the difference between the upper and lower limits of the IQR by 1.35.
[c]Mean and SD calculated for the whole cohorts based on values for male and female.
[d]CI calculated by Wald-type method.
[e]Tau² calculated by Restricted Maximum-Likelihood method.

Risk of bias legend
(A) Eligibility & Sampling
(B) Population & Setting Reporting
(C) Exposure Ascertainment
(D) Outcome Definition / Diagnostic Criteria
(E) Confounders—Identification
(F) Confounders—Control/Adjustment
(G) Outcome Measurement Validity/Reliability
(H) Statistical Methods Appropriateness

Note: A red circle means high risk of bias, while a green circle means low risk of bias.

Figure S4F

Study or Subgroup	MD	SE	Weight	Mean difference IV, Random, 95% CI [mL/kg*min]	Risk of Bias A	B	C	D	E	F	G	H
✓ Aldossari et al.	-9.28	2.848575	5.0%	-9.28 [-14.86 , -3.70]	−	+	+	−	+	−	−	+
✓ Stojmenović et al.	-5.03	1.625557	9.5%	-5.03 [-8.22 , -1.84]	−	−	+	−	−	−	+	+
✓ Parpa et al.	-3.01	1.514377	10.1%	-3.01 [-5.98 , -0.04]	+	−	+	+	−	−	+	+
✓ Śliż et al.	-2.84	1.525196	10.0%	-2.84 [-5.83 , 0.15]	+	+	+	+	+	−	+	+
✓ Bruzzese et al.	-2.7	2.303259	6.6%	-2.70 [-7.21 , 1.81]	−	−	+	−	−	−	+	+
× Roczniok et al.[a]	-2.42	1.840775	0.0%	-2.42 [-6.03 , 1.19]	+	−	−	−	−	−	+	+
✓ Keller et al.[b]	-1.9	0.631563	15.3%	-1.90 [-3.14 , -0.66]	+	−	−	−	+	−	−	−
✓ Moulson et al.	-1.8	2.477422	6.0%	-1.80 [-6.66 , 3.06]	+	+	−	+	+	+	+	+
✓ Stavrou et al.	0.3	1.423376	10.6%	0.30 [-2.49 , 3.09]	+	+	−	−	+	−	+	+
✓ Csulak et al. (longitudinal)[c]	0.65	1.772718	8.8%	0.65 [-2.82 , 4.12]	−	+	+	+	+	+	+	+
✓ Komici et al.[b]	1.1	1.965994	7.9%	1.10 [-2.75 , 4.95]	+	+	+	+	+	−	+	+
✓ Csulak et al.[c]	1.3	1.488923	10.2%	1.30 [-1.62 , 4.22]	−	+	+	+	+	+	+	+
**Total (Wald[d])**			**100.0%**	**-1.79 [-3.27 , -0.32]**								

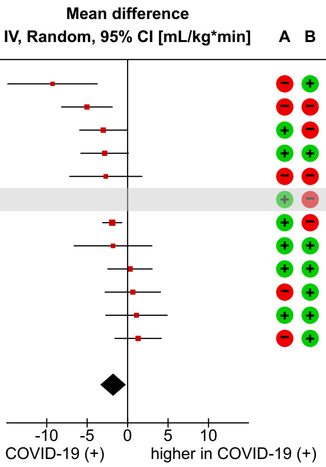

Test for overall effect: Z = 2.38 (P = 0.02)

Heterogeneity: Tau^2 (REML[e]) = 3.31; Chi^2 = 22.69, df = 10 (P = 0.01); I^2 = 60%

Footnotes

[a]Combined mean±SD for COVID-19(+) athletes from weighted mean and pooled sample SD using groups with and without symptoms.
[b]Assumptions: mean = median; SD calculated by dividing the difference between the upper and lower limits of the IQR by 1.35.
[c]Mean and SD calculated for the whole cohorts based on values for male and female.
[d]CI calculated by Wald-type method.
[e]Tau^2 calculated by Restricted Maximum-Likelihood method.

Risk of bias legend

(A) Eligibility & Sampling
(B) Population & Setting Reporting
(C) Exposure Ascertainment
(D) Outcome Definition / Diagnostic Criteria
(E) Confounders—Identification
(F) Confounders—Control/Adjustment
(G) Outcome Measurement Validity/Reliability
(H) Statistical Methods Appropriateness

Note: A red circle means high risk of bias, while a green circle means low risk of bias.

Figure S4G

Study or Subgroup	MD	SE	Weight	Mean difference IV, Random, 95% CI [mL/kg*min]	A	B	C	D	E	F	G	H
✓ Aldossari et al.	-9.28	2.848575	5.5%	-9.28 [-14.86 , -3.70]	−	+	+	−	+	−	−	+
✓ Stojmenović et al.	-5.03	1.625557	10.1%	-5.03 [-8.22 , -1.84]	−	−	+	−	−	−	+	+
✓ Parpa et al.	-3.01	1.514377	10.7%	-3.01 [-5.98 , -0.04]	+	−	+	+	−	−	+	+
✓ Śliż et al.	-2.84	1.525196	10.7%	-2.84 [-5.83 , 0.15]	+	+	+	+	+	−	+	+
✓ Bruzzese et al.	-2.7	2.303259	7.2%	-2.70 [-7.21 , 1.81]	−	−	+	−	−	−	+	+
✓ Roczniok et al.[a]	-2.42	1.840775	9.1%	-2.42 [-6.03 , 1.19]	+	−	−	−	−	−	+	+
× Keller et al.[b]	-1.9	0.631563	0.0%	-1.90 [-3.14 , -0.66]	+	−	−	−	+	−	●	−
✓ Moulson et al.	-1.8	2.477422	6.6%	-1.80 [-6.66 , 3.06]	+	+	−	+	+	+	+	+
✓ Stavrou et al.	0.3	1.423376	11.2%	0.30 [-2.49 , 3.09]	+	+	−	−	+	−	+	+
✓ Csulak et al. (longitudinal)[c]	0.65	1.772718	9.4%	0.65 [-2.82 , 4.12]	−	+	+	+	+	+	+	+
✓ Komici et al.[b]	1.1	1.965994	8.5%	1.10 [-2.75 , 4.95]	+	+	+	+	+	−	+	+
✓ Csulak et al.[c]	1.3	1.488923	10.9%	1.30 [-1.62 , 4.22]	−	+	+	+	+	+	+	+
**Total (Wald[d])**			**100.0%**	**-1.85 [-3.45 , -0.26]**								

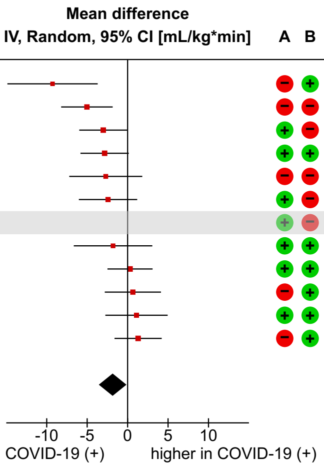

Test for overall effect: Z = 2.28 (P = 0.02)

Heterogeneity: Tau^2 (REML[e]) = 3.88; Chi^2 = 22.74, df = 10 (P = 0.01); I^2 = 55%

Footnotes

[a]Combined mean±SD for COVID-19(+) athletes from weighted mean and pooled sample SD using groups with and without symptoms.
[b]Assumptions: mean = median; SD calculated by dividing the difference between the upper and lower limits of the IQR by 1.35.
[c]Mean and SD calculated for the whole cohorts based on values for male and female.
[d]CI calculated by Wald-type method.
[e]Tau^2 calculated by Restricted Maximum-Likelihood method.

Risk of bias legend

(A) Eligibility & Sampling
(B) Population & Setting Reporting
(C) Exposure Ascertainment
(D) Outcome Definition / Diagnostic Criteria
(E) Confounders—Identification
(F) Confounders—Control/Adjustment
(G) Outcome Measurement Validity/Reliability
(H) Statistical Methods Appropriateness

Note: A red circle means high risk of bias, while a green circle means low risk of bias.

Figure S4H

Study or Subgroup	MD	SE	Weight	Mean difference IV, Random, 95% CI [mL/kg*min]	A	B	C	D	E	F	G	H
✓ Aldossari et al.	-9.28	2.848575	4.8%	-9.28 [-14.86 , -3.70]	−	+	+	−	+	−	−	+
✓ Stojmenović et al.	-5.03	1.625557	9.3%	-5.03 [-8.22 , -1.84]	−	−	+	−	−	−	+	+
✓ Parpa et al.	-3.01	1.514377	9.9%	-3.01 [-5.98 , -0.04]	+	−	+	+	−	−	+	+
✓ Śliż et al.	-2.84	1.525196	9.8%	-2.84 [-5.83 , 0.15]	+	+	+	+	+	−	+	+
✓ Bruzzese et al.	-2.7	2.303259	6.4%	-2.70 [-7.21 , 1.81]	−	−	+	−	−	−	+	+
✓ Roczniok et al.[a]	-2.42	1.840775	8.2%	-2.42 [-6.03 , 1.19]	+	−	−	−	−	−	+	+
✓ Keller et al.[b]	-1.9	0.631563	15.1%	-1.90 [-3.14 , -0.66]	+	−	−	−	+	−	−	−
✗ Moulson et al.	-1.8	2.477422	0.0%	-1.80 [-6.66 , 3.06]	+	+	−	+	+	+	+	+
✓ Stavrou et al.	0.3	1.423376	10.4%	0.30 [-2.49 , 3.09]	+	+	−	−	+	−	+	+
✓ Csulak et al. (longitudinal)[c]	0.65	1.772718	8.5%	0.65 [-2.82 , 4.12]	−	+	+	+	+	+	+	+
✓ Komici et al.[b]	1.1	1.965994	7.7%	1.10 [-2.75 , 4.95]	+	+	+	+	+	−	+	+
✓ Csulak et al.[c]	1.3	1.488923	10.0%	1.30 [-1.62 , 4.22]	−	+	+	+	+	+	+	+
**Total (Wald[d])**			**100.0%**	**-1.84 [-3.28 , -0.40]**								

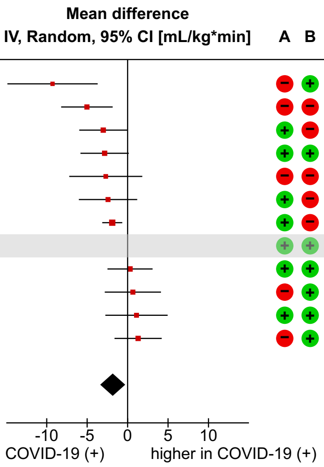

Test for overall effect: Z = 2.51 (P = 0.01)

Heterogeneity: Tau^2 (REML[e]) = 3.17; Chi^2 = 22.83, df = 10 (P = 0.01); I^2 = 59%

Footnotes

[a]Combined mean±SD for COVID-19(+) athletes from weighted mean and pooled sample SD using groups with and without symptoms.
[b]Assumptions: mean = median; SD calculated by dividing the difference between the upper and lower limits of the IQR by 1.35.
[c]Mean and SD calculated for the whole cohorts based on values for male and female.
[d]CI calculated by Wald-type method.
[e]Tau^2 calculated by Restricted Maximum-Likelihood method.

Risk of bias legend

(A) Eligibility & Sampling
(B) Population & Setting Reporting
(C) Exposure Ascertainment
(D) Outcome Definition / Diagnostic Criteria
(E) Confounders—Identification
(F) Confounders—Control/Adjustment
(G) Outcome Measurement Validity/Reliability
(H) Statistical Methods Appropriateness

Note: A red circle means high risk of bias, while a green circle means low risk of bias.

Figure S4I

Study or Subgroup	MD	SE	Weight	Mean difference IV, Random, 95% CI [mL/kg*min]
✓ Aldossari et al.	-9.28	2.848575	4.8%	-9.28 [-14.86 , -3.70]
✓ Stojmenović et al.	-5.03	1.625557	9.7%	-5.03 [-8.22 , -1.84]
✓ Parpa et al.	-3.01	1.514377	10.4%	-3.01 [-5.98 , -0.04]
✓ Śliż et al.	-2.84	1.525196	10.3%	-2.84 [-5.83 , 0.15]
✓ Bruzzese et al.	-2.7	2.303259	6.5%	-2.70 [-7.21 , 1.81]
✓ Roczniok et al.[a]	-2.42	1.840775	8.5%	-2.42 [-6.03 , 1.19]
✓ Keller et al.[b]	-1.9	0.631563	16.7%	-1.90 [-3.14 , -0.66]
✓ Moulson et al.	-1.8	2.477422	5.9%	-1.80 [-6.66 , 3.06]
✕ Stavrou et al.	0.3	1.423376	0.0%	0.30 [-2.49 , 3.09]
✓ Csulak et al. (longitudinal)[c]	0.65	1.772718	8.9%	0.65 [-2.82 , 4.12]
✓ Komici et al.[b]	1.1	1.965994	7.9%	1.10 [-2.75 , 4.95]
✓ Csulak et al.[c]	1.3	1.488923	10.5%	1.30 [-1.62 , 4.22]
**Total (Wald[d])**			**100.0%**	**-2.06 [-3.47 , -0.65]**

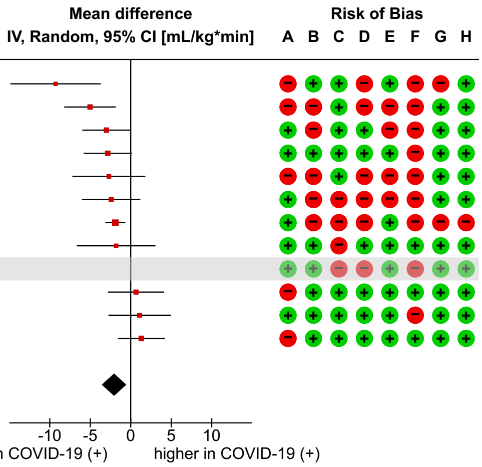

Test for overall effect: Z = 2.85 (P = 0.004)

Heterogeneity: Tau² (REML[e]) = 2.73; Chi² = 20.55, df = 10 (P = 0.02); I² = 54%

Footnotes

[a]Combined mean±SD for COVID-19(+) athletes from weighted mean and pooled sample SD using groups with and without symptoms.
[b]Assumptions: mean = median; SD calculated by dividing the difference between the upper and lower limits of the IQR by 1.35.
[c]Mean and SD calculated for the whole cohorts based on values for male and female.
[d]CI calculated by Wald-type method.
[e]Tau² calculated by Restricted Maximum-Likelihood method.

Risk of bias legend

(A) Eligibility & Sampling
(B) Population & Setting Reporting
(C) Exposure Ascertainment
(D) Outcome Definition / Diagnostic Criteria
(E) Confounders—Identification
(F) Confounders—Control/Adjustment
(G) Outcome Measurement Validity/Reliability
(H) Statistical Methods Appropriateness

Note: A red circle means high risk of bias, while a green circle means low risk of bias.

Figure S4J

Study or Subgroup	MD	SE	Weight	Mean difference IV, Random, 95% CI [mL/kg*min]	A	B	C	D	E	F	G	H
✓ Aldossari et al.	-9.28	2.848575	4.7%	-9.28 [-14.86 , -3.70]	−	+	+	−	+	−	−	+
✓ Stojmenović et al.	-5.03	1.625557	9.5%	-5.03 [-8.22 , -1.84]	−	−	+	−	−	−	+	+
✓ Parpa et al.	-3.01	1.514377	10.2%	-3.01 [-5.98 , -0.04]	+	−	+	+	−	−	+	+
✓ Śliż et al.	-2.84	1.525196	10.1%	-2.84 [-5.83 , 0.15]	+	+	+	+	+	−	+	+
✓ Bruzzese et al.	-2.7	2.303259	6.3%	-2.70 [-7.21 , 1.81]	−	−	+	−	−	−	+	+
✓ Roczniok et al.[a]	-2.42	1.840775	8.3%	-2.42 [-6.03 , 1.19]	+	−	−	−	−	−	+	+
✓ Keller et al.[b]	-1.9	0.631563	16.4%	-1.90 [-3.14 , -0.66]	+	−	−	−	+	−	−	−
✓ Moulson et al.	-1.8	2.477422	5.7%	-1.80 [-6.66 , 3.06]	+	+	−	+	+	+	+	+
✓ Stavrou et al.	0.3	1.423376	10.7%	0.30 [-2.49 , 3.09]	+	+	−	−	+	−	+	+
✕ Csulak et al. (longitudinal)[c]	0.65	1.772718	0.0%	0.65 [-2.82 , 4.12]	−	+	+	+	+	+	+	+
✓ Komici et al.[b]	1.1	1.965994	7.7%	1.10 [-2.75 , 4.95]	+	+	+	+	+	−	+	+
✓ Csulak et al.[c]	1.3	1.488923	10.3%	1.30 [-1.62 , 4.22]	−	+	+	+	+	+	+	+
**Total (Wald[d])**			**100.0%**	**-2.04 [-3.44 , -0.64]**								

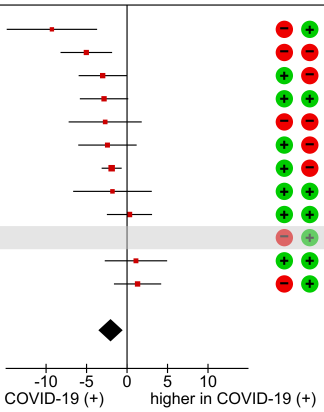

Test for overall effect: Z = 2.86 (P = 0.004)

Heterogeneity: Tau² (REML[e]) = 2.71; Chi² = 20.88, df = 10 (P = 0.02); I² = 54%

Footnotes

[a]Combined mean±SD for COVID-19(+) athletes from weighted mean and pooled sample SD using groups with and without symptoms.
[b]Assumptions: mean = median; SD calculated by dividing the difference between the upper and lower limits of the IQR by 1.35.
[c]Mean and SD calculated for the whole cohorts based on values for male and female.
[d]CI calculated by Wald-type method.
[e]Tau² calculated by Restricted Maximum-Likelihood method.

Risk of bias legend

(A) Eligibility & Sampling
(B) Population & Setting Reporting
(C) Exposure Ascertainment
(D) Outcome Definition / Diagnostic Criteria
(E) Confounders—Identification
(F) Confounders—Control/Adjustment
(G) Outcome Measurement Validity/Reliability
(H) Statistical Methods Appropriateness

Note: A red circle means high risk of bias, while a green circle means low risk of bias.

Figure S4K

Study or Subgroup	MD	SE	Weight	Mean difference IV, Random, 95% CI [mL/kg*min]	A	B	C	D	E	F	G	H
✓ Aldossari et al.	-9.28	2.848575	4.6%	-9.28 [-14.86 , -3.70]	−	+	+	−	+	−	−	+
✓ Stojmenović et al.	-5.03	1.625557	9.4%	-5.03 [-8.22 , -1.84]	−	−	+	−	−	−	+	+
✓ Parpa et al.	-3.01	1.514377	10.1%	-3.01 [-5.98 , -0.04]	+	−	+	+	−	−	+	+
✓ Śliż et al.	-2.84	1.525196	10.0%	-2.84 [-5.83 , 0.15]	+	+	+	+	+	−	+	+
✓ Bruzzese et al.	-2.7	2.303259	6.2%	-2.70 [-7.21 , 1.81]	−	−	+	−	−	−	+	+
✓ Roczniok et al.[a]	-2.42	1.840775	8.2%	-2.42 [-6.03 , 1.19]	+	−	−	−	−	−	+	+
✓ Keller et al.[b]	-1.9	0.631563	16.5%	-1.90 [-3.14 , -0.66]	+	−	−	−	+	−	−	−
✓ Moulson et al.	-1.8	2.477422	5.6%	-1.80 [-6.66 , 3.06]	+	+	−	+	+	+	+	+
✓ Stavrou et al.	0.3	1.423376	10.6%	0.30 [-2.49 , 3.09]	+	+	−	−	+	−	+	+
✓ Csulak et al. (longitudinal)[c]	0.65	1.772718	8.6%	0.65 [-2.82 , 4.12]	−	+	+	+	+	+	+	+
✕ Komici et al.[b]	1.1	1.965994	0.0%	1.10 [-2.75 , 4.95]	+	+	+	+	+	−	+	+
✓ Csulak et al.[c]	1.3	1.488923	10.2%	1.30 [-1.62 , 4.22]	−	+	+	+	+	+	+	+
**Total (Wald[d])**			**100.0%**	**-2.04 [-3.42 , -0.67]**								

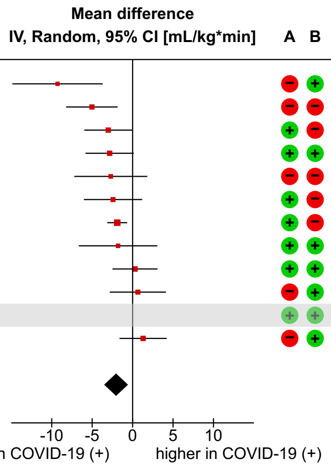

Test for overall effect: Z = 2.92 (P = 0.003)

Heterogeneity: Tau^2 (REML[e]) = 2.57; Chi^2 = 20.62, df = 10 (P = 0.02); I^2 = 54%

Footnotes

[a]Combined mean±SD for COVID-19(+) athletes from weighted mean and pooled sample SD using groups with and without symptoms.
[b]Assumptions: mean = median; SD calculated by dividing the difference between the upper and lower limits of the IQR by 1.35.
[c]Mean and SD calculated for the whole cohorts based on values for male and female.
[d]CI calculated by Wald-type method.
[e]Tau^2 calculated by Restricted Maximum-Likelihood method.

Risk of bias legend

(A) Eligibility & Sampling
(B) Population & Setting Reporting
(C) Exposure Ascertainment
(D) Outcome Definition / Diagnostic Criteria
(E) Confounders—Identification
(F) Confounders—Control/Adjustment
(G) Outcome Measurement Validity/Reliability
(H) Statistical Methods Appropriateness

Note: A red circle means high risk of bias, while a green circle means low risk of bias.

Figure S4L

Study or Subgroup	MD	SE	Weight	Mean difference IV, Random, 95% CI [mL/kg*min]	A	B	C	D	E	F	G	H
✓ Aldossari et al.	-9.28	2.848575	4.2%	-9.28 [-14.86 , -3.70]	−	+	+	−	+	−	−	+
✓ Stojmenović et al.	-5.03	1.625557	9.5%	-5.03 [-8.22 , -1.84]	−	−	+	−	−	−	+	+
✓ Parpa et al.	-3.01	1.514377	10.3%	-3.01 [-5.98 , -0.04]	+	−	+	+	−	−	+	+
✓ Śliż et al.	-2.84	1.525196	10.2%	-2.84 [-5.83 , 0.15]	+	+	+	+	+	−	+	+
✓ Bruzzese et al.	-2.7	2.303259	5.9%	-2.70 [-7.21 , 1.81]	−	−	+	−	−	−	+	+
✓ Roczniok et al.[a]	-2.42	1.840775	8.1%	-2.42 [-6.03 , 1.19]	+	−	−	−	−	−	+	+
✓ Keller et al.[b]	-1.9	0.631563	19.6%	-1.90 [-3.14 , -0.66]	+	−	−	−	+	−	−	−
✓ Moulson et al.	-1.8	2.477422	5.3%	-1.80 [-6.66 , 3.06]	+	+	−	+	+	+	+	+
✓ Stavrou et al.	0.3	1.423376	11.0%	0.30 [-2.49 , 3.09]	+	+	−	−	+	−	+	+
✓ Csulak et al. (longitudinal)[c]	0.65	1.772718	8.5%	0.65 [-2.82 , 4.12]	−	+	+	+	+	+	+	+
✓ Komici et al.[b]	1.1	1.965994	7.4%	1.10 [-2.75 , 4.95]	+	+	+	+	+	−	+	+
✕ Csulak et al.[c]	1.3	1.488923	0.0%	1.30 [-1.62 , 4.22]	−	+	+	+	+	+	+	+
**Total (Wald[d])**			**100.0%**	**-2.12 [-3.38 , -0.86]**								

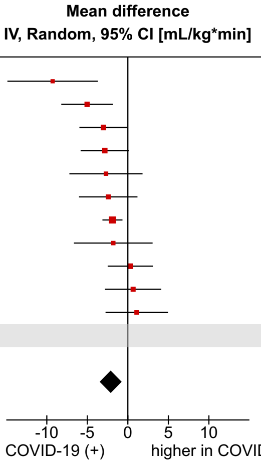

Test for overall effect: Z = 3.30 (P = 0.0010)

Heterogeneity: Tau² (REML[e]) = 1.71; Chi² = 18.26, df = 10 (P = 0.05); I² = 42%

Footnotes

[a]Combined mean±SD for COVID-19(+) athletes from weighted mean and pooled sample SD using groups with and without symptoms.
[b]Assumptions: mean = median; SD calculated by dividing the difference between the upper and lower limits of the IQR by 1.35.
[c]Mean and SD calculated for the whole cohorts based on values for male and female.
[d]CI calculated by Wald-type method.
[e]Tau² calculated by Restricted Maximum-Likelihood method.

Risk of bias legend
(A) Eligibility & Sampling
(B) Population & Setting Reporting
(C) Exposure Ascertainment
(D) Outcome Definition / Diagnostic Criteria
(E) Confounders—Identification
(F) Confounders—Control/Adjustment
(G) Outcome Measurement Validity/Reliability
(H) Statistical Methods Appropriateness

Note: A red circle means high risk of bias, while a green circle means low risk of bias.

Figure S5: Forest plot presenting the sensitivity analysis regarding VO_{2max} performed by removing studies with a high risk of bias in 4 or more domains.

Study or Subgroup	MD	SE	Weight	Mean difference IV, Random, 95% CI [mL/kg*min]
✕ Aldossari et al.	-9.28	2.848575	0.0%	-9.28 [-14.86 , -3.70]
✕ Stojmenović et al.	-5.03	1.625557	0.0%	-5.03 [-8.22 , -1.84]
✓ Parpa et al.	-3.01	1.514377	16.5%	-3.01 [-5.98 , -0.04]
✓ Šliž et al.	-2.84	1.525196	16.3%	-2.84 [-5.83 , 0.15]
✕ Bruzzese et al.	-2.7	2.303259	0.0%	-2.70 [-7.21 , 1.81]
✕ Roczniok et al.[a]	-2.42	1.840775	0.0%	-2.42 [-6.03 , 1.19]
✕ Keller et al.[b]	-1.9	0.631563	0.0%	-1.90 [-3.14 , -0.66]
✓ Moulson et al.	-1.8	2.477422	7.9%	-1.80 [-6.66 , 3.06]
✓ Stavrou et al.	0.3	1.423376	17.8%	0.30 [-2.49 , 3.09]
✓ Csulak et al. (longitudinal)[c]	0.65	1.772718	13.3%	0.65 [-2.82 , 4.12]
✓ Komici et al.[b]	1.1	1.965994	11.4%	1.10 [-2.75 , 4.95]
✓ Csulak et al.[c]	1.3	1.488923	16.8%	1.30 [-1.62 , 4.22]
**Total (Wald[d])**			**100.0%**	**-0.62 [-2.11 , 0.88]**

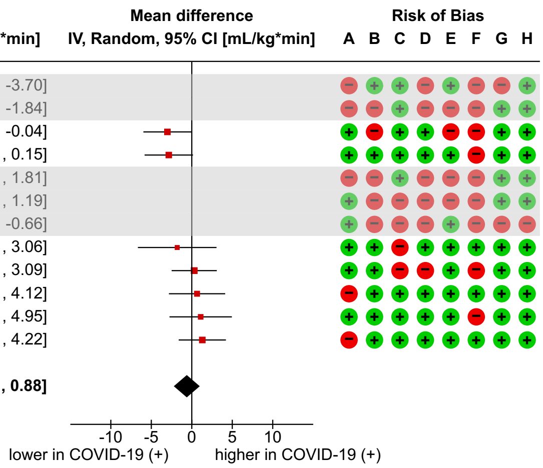

Test for overall effect: Z = 0.81 (P = 0.42)

Heterogeneity: Tau² (REML[e]) = 1.23; Chi² = 8.20, df = 6 (P = 0.22); I² = 31%

Footnotes

[a]Combined mean±SD for COVID-19(+) athletes from weighted mean and pooled sample SD using groups with and without symptoms.
[b]Assumptions: mean = median; SD calculated by dividing the difference between the upper and lower limits of the IQR by 1.35.
[c]Mean and SD calculated for the whole cohorts based on values for male and female.
[d]CI calculated by Wald-type method.
[e]Tau² calculated by Restricted Maximum-Likelihood method.

Risk of bias legend

(A) Eligibility & Sampling
(B) Population & Setting Reporting
(C) Exposure Ascertainment
(D) Outcome Definition / Diagnostic Criteria
(E) Confounders—Identification
(F) Confounders—Control/Adjustment
(G) Outcome Measurement Validity/Reliability
(H) Statistical Methods Appropriateness

Note: A red circle means high risk of bias, while a green circle means low risk of bias.

Figure S6A: Forest plot presenting the absolute VO_{2max} subgroup analysis based on competition level.

Study or Subgroup	MD	SE	Weight	Mean difference IV, Random, 95% CI [L/min]
**4.2.1 Recreational**				
**Subtotal**				**Not estimable**
Test for overall effect: Not applicable				
Heterogeneity: Not applicable				
**4.2.2 Competitive**				
Fikenzer et al. (longitudinal)	-0.292	0.258256	7.5%	-0.29 [-0.80 , 0.21]
Fikenzer et al.	-0.121	0.182825	15.0%	-0.12 [-0.48 , 0.24]
Bruzzese et al.	-0.0401	0.207192	11.7%	-0.04 [-0.45 , 0.37]
Csulak et al. (longitudinal)[a]	0.2	0.382193	3.4%	0.20 [-0.55 , 0.95]
Csulak et al.[a]	0.26	0.350239	4.1%	0.26 [-0.43 , 0.95]
**Subtotal (Wald[b])**			**41.8%**	**-0.07 [-0.28 , 0.15]**
Test for overall effect: Z = 0.60 (P = 0.55)				
Heterogeneity: Tau^2 (REML[c]) = 0.00; Chi^2 = 2.22, df = 4 (P = 0.69); I^2 = 0%				
**4.2.3 Mixed**				
Śliż et al.	-0.21747	0.103997	46.4%	-0.22 [-0.42 , -0.01]
Moulson et al.	-0.2	0.206444	11.8%	-0.20 [-0.60 , 0.20]
**Subtotal (Wald[b])**			**58.2%**	**-0.21 [-0.40 , -0.03]**
Test for overall effect: Z = 2.30 (P = 0.02)				
Heterogeneity: Tau^2 (REML[c]) = 0.00; Chi^2 = 0.01, df = 1 (P = 0.94); I^2 = 0%				
**Total (Wald[b])**			**100.0%**	**-0.15 [-0.29 , -0.01]**

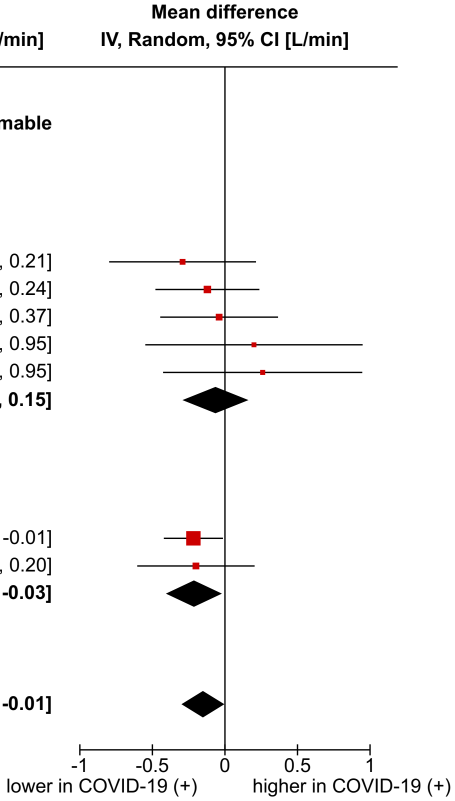

Test for overall effect: Z = 2.14 (P = 0.03)
Test for subgroup differences: Chi^2 = 1.07, df = 1 (P = 0.30), I^2 = 6.4%
Heterogeneity: Tau^2 (REML[c]) = 0.00; Chi^2 = 3.30, df = 6 (P = 0.77); I^2 = 0%

Footnotes

[a]Mean and SD calculated for the whole cohorts based on values for male and female.
[b]CI calculated by Wald-type method.
[c]Tau^2 calculated by Restricted Maximum-Likelihood method.

Figure S6B: Forest plot presenting the absolute VO_{2max} subgroup analysis based on CPET modality.

Study or Subgroup	MD	SE	Weight	Mean difference IV, Random, 95% CI [L/min]
**4.3.1 Cycle ergometer**				
Fikenzer et al. (longitudinal)	-0.292	0.258256	7.5%	-0.29 [-0.80 , 0.21]
Fikenzer et al.	-0.121	0.182825	15.0%	-0.12 [-0.48 , 0.24]
**Subtotal (Wald[a])**			**22.6%**	**-0.18 [-0.47 , 0.11]**
Test for overall effect: Z = 1.19 (P = 0.23)				
Heterogeneity: Tau² (REML[b]) = 0.00; Chi² = 0.29, df = 1 (P = 0.59); I² = 0%				
**4.3.2 Mixed**				
Śliż et al.	-0.21747	0.103997	46.4%	-0.22 [-0.42 , -0.01]
Moulson et al.	-0.2	0.206444	11.8%	-0.20 [-0.60 , 0.20]
**Subtotal (Wald[a])**			**58.2%**	**-0.21 [-0.40 , -0.03]**
Test for overall effect: Z = 2.30 (P = 0.02)				
Heterogeneity: Tau² (REML[b]) = 0.00; Chi² = 0.01, df = 1 (P = 0.94); I² = 0%				
**4.3.3 Treadmill**				
Bruzzese et al.	-0.0401	0.207192	11.7%	-0.04 [-0.45 , 0.37]
Csulak et al. (longitudinal)[c]	0.2	0.382193	3.4%	0.20 [-0.55 , 0.95]
Csulak et al.[c]	0.26	0.350239	4.1%	0.26 [-0.43 , 0.95]
**Subtotal (Wald[a])**			**19.2%**	**0.07 [-0.25 , 0.38]**
Test for overall effect: Z = 0.41 (P = 0.68)				
Heterogeneity: Tau² (REML[b]) = 0.00; Chi² = 0.69, df = 2 (P = 0.71); I² = 0%				
**Total (Wald[a])**			**100.0%**	**-0.15 [-0.29 , -0.01]**

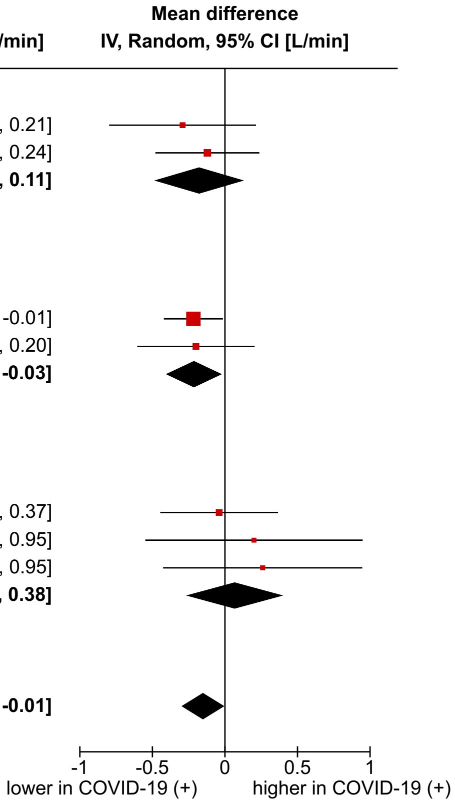

Test for overall effect: Z = 2.14 (P = 0.03)
Test for subgroup differences: Chi² = 2.31, df = 2 (P = 0.32), I² = 13.3%
Heterogeneity: Tau² (REML[b]) = 0.00; Chi² = 3.30, df = 6 (P = 0.77); I² = 0%

Footnotes
[a]CI calculated by Wald-type method.
[b]Tau² calculated by Restricted Maximum-Likelihood method.
[c]Mean and SD calculated for the whole cohorts based on values for male and female.

Figure S6C: Forest plot presenting the absolute VO_{2max} subgroup analysis based on sex distribution.

Study or Subgroup	MD	SE	Weight	Mean difference IV, Random, 95% CI [L/min]
**4.4.1 Mixed**				
Śliż et al.	-0.21747	0.103997	46.4%	-0.22 [-0.42 , -0.01]
Moulson et al.	-0.2	0.206444	11.8%	-0.20 [-0.60 , 0.20]
Bruzzese et al.	-0.0401	0.207192	11.7%	-0.04 [-0.45 , 0.37]
Csulak et al. (longitudinal)[a]	0.2	0.382193	3.4%	0.20 [-0.55 , 0.95]
Csulak et al.[a]	0.26	0.350239	4.1%	0.26 [-0.43 , 0.95]
**Subtotal (Wald[b])**			**77.4%**	**-0.14 [-0.30 , 0.01]**
Test for overall effect: Z = 1.79 (P = 0.07)				
Heterogeneity: Tau² (REML[c]) = 0.00; Chi² = 2.96, df = 4 (P = 0.56); I² = 0%				
**4.4.2 Not reported**				
**Subtotal**				**Not estimable**
Test for overall effect: Not applicable				
Heterogeneity: Not applicable				
**4.4.3 Only male**				
Fikenzer et al. (longitudinal)	-0.292	0.258256	7.5%	-0.29 [-0.80 , 0.21]
Fikenzer et al.	-0.121	0.182825	15.0%	-0.12 [-0.48 , 0.24]
**Subtotal (Wald[b])**			**22.6%**	**-0.18 [-0.47 , 0.11]**
Test for overall effect: Z = 1.19 (P = 0.23)				
Heterogeneity: Tau² (REML[c]) = 0.00; Chi² = 0.29, df = 1 (P = 0.59); I² = 0%				
**Total (Wald[b])**			**100.0%**	**-0.15 [-0.29 , -0.01]**

Mean difference IV, Random, 95% CI [L/min]

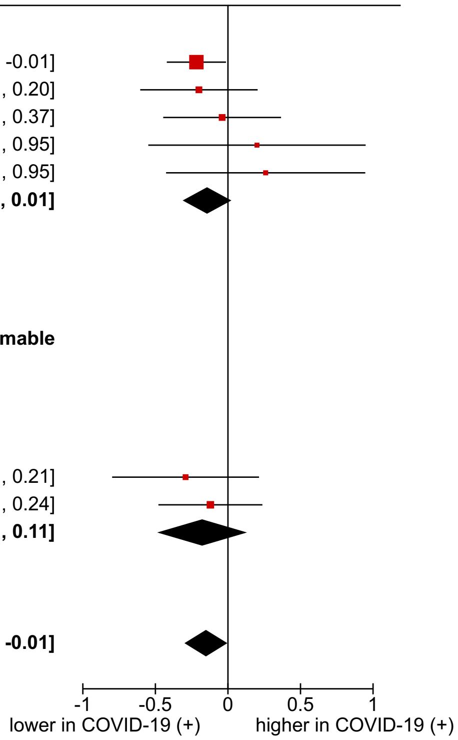

Test for overall effect: Z = 2.14 (P = 0.03)
Test for subgroup differences: Chi² = 0.04, df = 1 (P = 0.84), I² = 0%
Heterogeneity: Tau² (REML[c]) = 0.00; Chi² = 3.30, df = 6 (P = 0.77); I² = 0%

Footnotes

[a]Mean and SD calculated for the whole cohorts based on values for male and female.
[b]CI calculated by Wald-type method.
[c]Tau² calculated by Restricted Maximum-Likelihood method.

Figure S6D: Forest plot presenting the absolute VO_{2max} subgroup analysis based on comparison type.

Study or Subgroup	MD	SE	Weight	Mean difference IV, Random, 95% CI [L/min]
**4.5.1 Post-COVID athletes vs. healthy reference**				
Moulson et al.	-0.2	0.206444	11.8%	-0.20 [-0.60 , 0.20]
Fikenzer et al.	-0.121	0.182825	15.0%	-0.12 [-0.48 , 0.24]
Csulak et al.[a]	0.26	0.350239	4.1%	0.26 [-0.43 , 0.95]
**Subtotal (Wald[b])**			**30.9%**	**-0.10 [-0.35 , 0.15]**
Test for overall effect: Z = 0.79 (P = 0.43)				
Heterogeneity: Tau^2 (REML[c]) = 0.00; Chi^2 = 1.30, df = 2 (P = 0.52); I^2 = 0%				
**4.5.2 Post-COVID athletes vs. the same athletes before infection**				
Fikenzer et al. (longitudinal)	-0.292	0.258256	7.5%	-0.29 [-0.80 , 0.21]
Śliż et al.	-0.21747	0.103997	46.4%	-0.22 [-0.42 , -0.01]
Bruzzese et al.	-0.0401	0.207192	11.7%	-0.04 [-0.45 , 0.37]
Csulak et al. (longitudinal)[a]	0.2	0.382193	3.4%	0.20 [-0.55 , 0.95]
**Subtotal (Wald[b])**			**69.1%**	**-0.17 [-0.34 , -0.01]**
Test for overall effect: Z = 2.05 (P = 0.04)				
Heterogeneity: Tau^2 (REML[c]) = 0.00; Chi^2 = 1.76, df = 3 (P = 0.62); I^2 = 0%				
**Total (Wald[b])**			**100.0%**	**-0.15 [-0.29 , -0.01]**

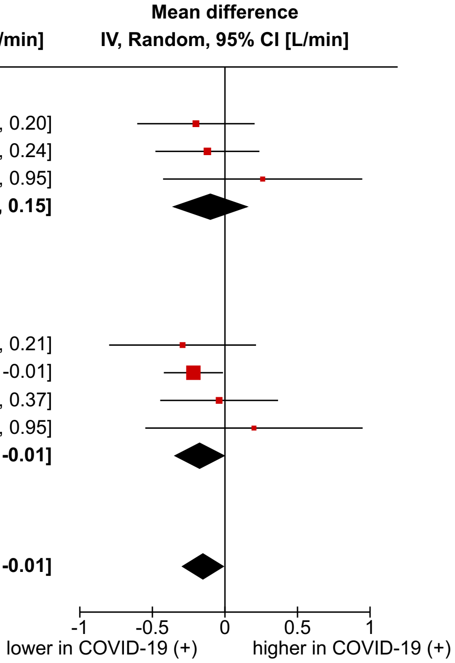

Test for overall effect: Z = 2.14 (P = 0.03)
Test for subgroup differences: Chi^2 = 0.23, df = 1 (P = 0.63), I^2 = 0%
Heterogeneity: Tau^2 (REML[c]) = 0.00; Chi^2 = 3.30, df = 6 (P = 0.77); I^2 = 0%

Footnotes

[a]Mean and SD calculated for the whole cohorts based on values for male and female.
[b]CI calculated by Wald-type method.
[c]Tau^2 calculated by Restricted Maximum-Likelihood method.

Figure S6E: Forest plot presenting the absolute VO_{2max} subgroup analysis based on the presence of symptoms in athletes during COVID-19 infection.

Study or Subgroup	MD	SE	Weight	Mean difference IV, Random, 95% CI [L/min]
**4.6.1 Mixed**				
Fikenzer et al. (longitudinal)	-0.292	0.258256	7.5%	-0.29 [-0.80 , 0.21]
Fikenzer et al.	-0.121	0.182825	15.0%	-0.12 [-0.48 , 0.24]
Bruzzese et al.	-0.0401	0.207192	11.7%	-0.04 [-0.45 , 0.37]
Csulak et al. (longitudinal)[a]	0.2	0.382193	3.4%	0.20 [-0.55 , 0.95]
Csulak et al.[a]	0.26	0.350239	4.1%	0.26 [-0.43 , 0.95]
**Subtotal (Wald[b])**			**41.8%**	**-0.07 [-0.28 , 0.15]**
Test for overall effect: Z = 0.60 (P = 0.55)				
Heterogeneity: Tau^2 (REML[c]) = 0.00; Chi^2 = 2.22, df = 4 (P = 0.69); I^2 = 0%				
**4.6.2 Only symptomatic**				
Moulson et al.	-0.2	0.206444	11.8%	-0.20 [-0.60 , 0.20]
**Subtotal**			**11.8%**	**-0.20 [-0.60 , 0.20]**
Test for overall effect: Z = 0.97 (P = 0.33)				
Heterogeneity: Not applicable				
**4.6.3 Not reported**				
Śliż et al.	-0.21747	0.103997	46.4%	-0.22 [-0.42 , -0.01]
**Subtotal**			**46.4%**	**-0.22 [-0.42 , -0.01]**
Test for overall effect: Z = 2.09 (P = 0.04)				
Heterogeneity: Not applicable				
**Total (Wald[b])**			**100.0%**	**-0.15 [-0.29 , -0.01]**

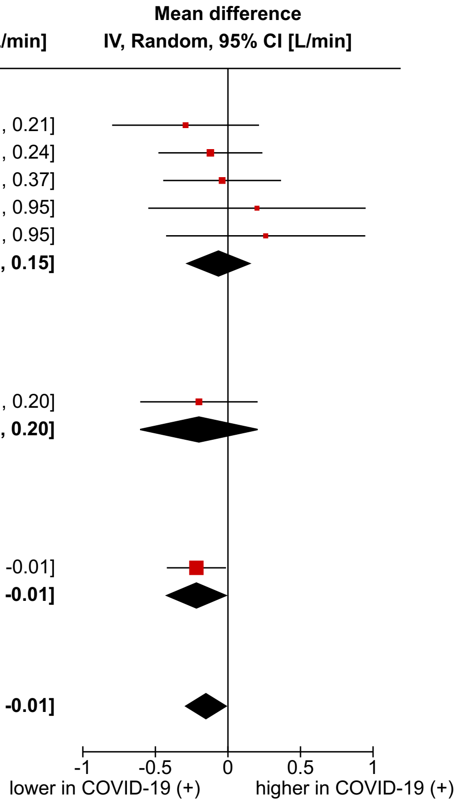

Test for overall effect: Z = 2.14 (P = 0.03)

Test for subgroup differences: Chi^2 = 1.07, df = 2 (P = 0.58), I^2 = 0%

Heterogeneity: Tau^2 (REML[c]) = 0.00; Chi^2 = 3.30, df = 6 (P = 0.77); I^2 = 0%

Footnotes

[a]Mean and SD calculated for the whole cohorts based on values for male and female.

[b]CI calculated by Wald-type method.

[c]Tau^2 calculated by Restricted Maximum-Likelihood method.

Figure S6F: Forest plot presenting the absolute VO_{2max} subgroup analysis based on time elapsed from COVID-19 infection to CPET.

Study or Subgroup	MD	SE	Weight	Mean difference IV, Random, 95% CI [L/min]
**4.7.1 Not reported**				
**Subtotal**				**Not estimable**
Test for overall effect: Not applicable				
Heterogeneity: Not applicable				
**4.7.2 Up to 1 month after infection**				
Fikenzer et al. (longitudinal)	-0.292	0.258256	7.5%	-0.29 [-0.80 , 0.21]
Fikenzer et al.	-0.121	0.182825	15.0%	-0.12 [-0.48 , 0.24]
Bruzzese et al.	-0.0401	0.207192	11.7%	-0.04 [-0.45 , 0.37]
Csulak et al. (longitudinal)[a]	0.2	0.382193	3.4%	0.20 [-0.55 , 0.95]
Csulak et al.[a]	0.26	0.350239	4.1%	0.26 [-0.43 , 0.95]
**Subtotal (Wald[b])**			**41.8%**	**-0.07 [-0.28 , 0.15]**
Test for overall effect: Z = 0.60 (P = 0.55)				
Heterogeneity: Tau^2 (REML[c]) = 0.00; Chi^2 = 2.22, df = 4 (P = 0.69); I^2 = 0%				
**4.7.3 More than 1 month after infection**				
Śliż et al.	-0.21747	0.103997	46.4%	-0.22 [-0.42 , -0.01]
Moulson et al.	-0.2	0.206444	11.8%	-0.20 [-0.60 , 0.20]
**Subtotal (Wald[b])**			**58.2%**	**-0.21 [-0.40 , -0.03]**
Test for overall effect: Z = 2.30 (P = 0.02)				
Heterogeneity: Tau^2 (REML[c]) = 0.00; Chi^2 = 0.01, df = 1 (P = 0.94); I^2 = 0%				
**Total (Wald[b])**			**100.0%**	**-0.15 [-0.29 , -0.01]**

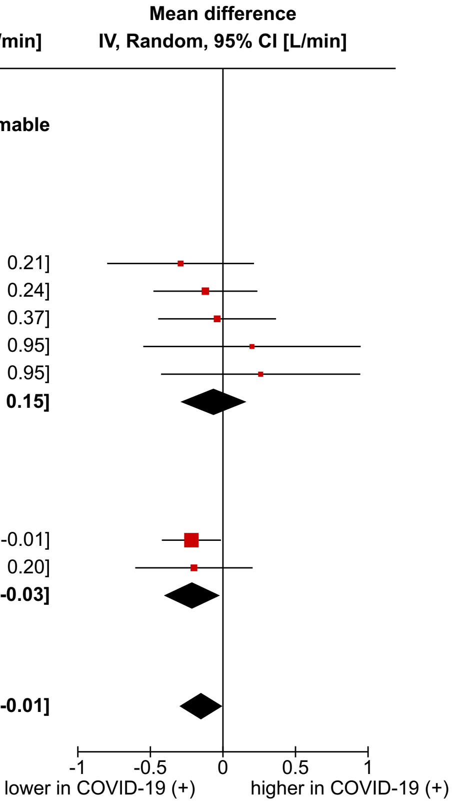

Test for overall effect: Z = 2.14 (P = 0.03)
Test for subgroup differences: Chi^2 = 1.07, df = 1 (P = 0.30), I^2 = 6.4%
Heterogeneity: Tau^2 (REML[c]) = 0.00; Chi^2 = 3.30, df = 6 (P = 0.77); I^2 = 0%

Footnotes
[a]Mean and SD calculated for the whole cohorts based on values for male and female.
[b]CI calculated by Wald-type method.
[c]Tau^2 calculated by Restricted Maximum-Likelihood method.

Figure S7A: Forest plot presenting the HR_{max} subgroup analysis based on competition level.

Study or Subgroup	MD	SE	Weight	Mean difference IV, Random, 95% CI [bpm]
**5.2.1 Recreational**				
Aldossari et al.	-0.81	0.862436	50.3%	-0.81 [-2.50 , 0.88]
**Subtotal**			**50.3%**	**-0.81 [-2.50 , 0.88]**
Test for overall effect: Z = 0.94 (P = 0.35)				
Heterogeneity: Not applicable				
**5.2.2 Competitive**				
Stavrou et al.	-5	2.596151	5.6%	-5.00 [-10.09 , 0.09]
Csulak et al. (longitudinal)	-3.2	3.815476	2.6%	-3.20 [-10.68 , 4.28]
Csulak et al.	-0.5	3.422086	3.2%	-0.50 [-7.21 , 6.21]
Stojmenović et al.	0.93	2.117791	8.3%	0.93 [-3.22 , 5.08]
Parpa et al.	1.34	2.407467	6.5%	1.34 [-3.38 , 6.06]
Bruzzese et al.	3.4	2.576238	5.6%	3.40 [-1.65 , 8.45]
Fikenzer et al.	7	7.28869	0.7%	7.00 [-7.29 , 21.29]
Fikenzer et al. (longitudinal)	7	7.254309	0.7%	7.00 [-7.22 , 21.22]
Komici et al.[a]	9	6.555026	0.9%	9.00 [-3.85 , 21.85]
**Subtotal (Wald[b])**			**34.1%**	**0.54 [-1.90 , 2.98]**
Test for overall effect: Z = 0.43 (P = 0.66)				
Heterogeneity: Tau² (REML[c]) = 2.86; Chi² = 10.22, df = 8 (P = 0.25); I² = 21%				
**5.2.3 Mixed**				
Śliż et al.	-0.96	2.024382	9.1%	-0.96 [-4.93 , 3.01]
Moulson et al.	0	2.405351	6.5%	0.00 [-4.71 , 4.71]
**Subtotal (Wald[b])**			**15.6%**	**-0.56 [-3.60 , 2.47]**
Test for overall effect: Z = 0.36 (P = 0.72)				
Heterogeneity: Tau² (REML[c]) = 0.00; Chi² = 0.09, df = 1 (P = 0.76); I² = 0%				
**Total (Wald[b])**			**100.0%**	**-0.34 [-1.54 , 0.86]**

Test for overall effect: Z = 0.55 (P = 0.58)
Test for subgroup differences: Chi² = 0.81, df = 2 (P = 0.67), I² = 0%
Heterogeneity: Tau² (REML[c]) = 0.00; Chi² = 11.22, df = 11 (P = 0.43); I² = 0%

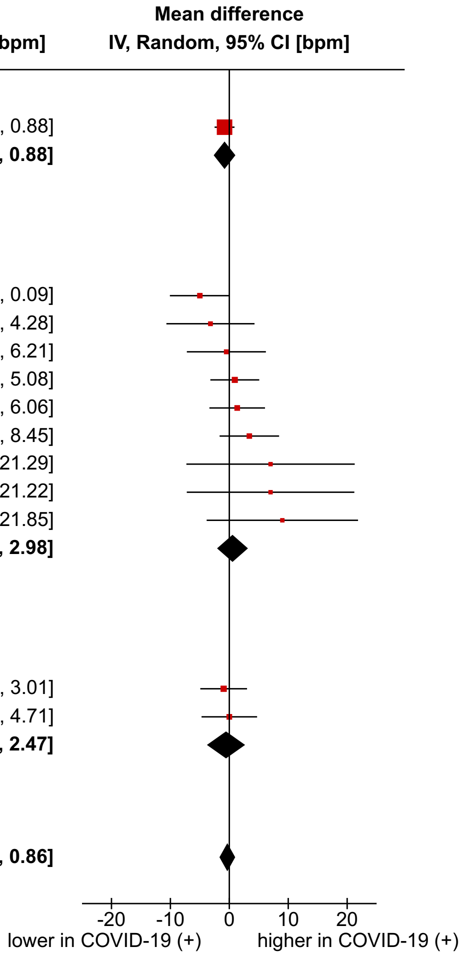

Footnotes
[a]Assumptions: mean = median; SD calculated by dividing the difference between the upper and lower limits of the IQR by 1.35.
[b]CI calculated by Wald-type method.
[c]Tau² calculated by Restricted Maximum-Likelihood method.

Figure S7B: Forest plot presenting the HR_{max} subgroup analysis based on CPET modality.

Study or Subgroup	MD	SE	Weight	Mean difference IV, Random, 95% CI [bpm]
**5.3.1 Cycle ergometer**				
Fikenzer et al.	7	7.28869	0.7%	7.00 [-7.29 , 21.29]
Fikenzer et al. (longitudinal)	7	7.254309	0.7%	7.00 [-7.22 , 21.22]
**Subtotal (Wald[a])**			**1.4%**	**7.00 [-3.08 , 17.08]**
Test for overall effect: Z = 1.36 (P = 0.17)				
Heterogeneity: Tau^2 (REML[b]) = 0.00; Chi^2 = 0.00, df = 1 (P = 1.00); I^2 = 0%				
**5.3.2 Mixed**				
Śliż et al.	-0.96	2.024382	9.1%	-0.96 [-4.93 , 3.01]
Moulson et al.	0	2.405351	6.5%	0.00 [-4.71 , 4.71]
**Subtotal (Wald[a])**			**15.6%**	**-0.56 [-3.60 , 2.47]**
Test for overall effect: Z = 0.36 (P = 0.72)				
Heterogeneity: Tau^2 (REML[b]) = 0.00; Chi^2 = 0.09, df = 1 (P = 0.76); I^2 = 0%				
**5.3.3 Treadmill**				
Stavrou et al.	-5	2.596151	5.6%	-5.00 [-10.09 , 0.09]
Csulak et al. (longitudinal)	-3.2	3.815476	2.6%	-3.20 [-10.68 , 4.28]
Aldossari et al.	-0.81	0.862436	50.3%	-0.81 [-2.50 , 0.88]
Csulak et al.	-0.5	3.422086	3.2%	-0.50 [-7.21 , 6.21]
Stojmenović et al.	0.93	2.117791	8.3%	0.93 [-3.22 , 5.08]
Parpa et al.	1.34	2.407467	6.5%	1.34 [-3.38 , 6.06]
Bruzzese et al.	3.4	2.576238	5.6%	3.40 [-1.65 , 8.45]
Komici et al.[c]	9	6.555026	0.9%	9.00 [-3.85 , 21.85]
**Subtotal (Wald[a])**			**83.0%**	**-0.42 [-1.74 , 0.90]**
Test for overall effect: Z = 0.63 (P = 0.53)				
Heterogeneity: Tau^2 (REML[b]) = 0.00; Chi^2 = 9.05, df = 7 (P = 0.25); I^2 = 0%				
**Total (Wald[a])**			**100.0%**	**-0.34 [-1.54 , 0.86]**

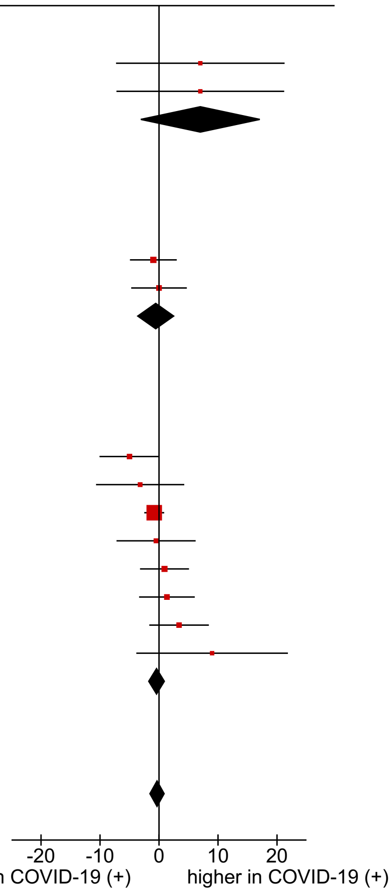

Test for overall effect: Z = 0.55 (P = 0.58)
Test for subgroup differences: Chi^2 = 2.07, df = 2 (P = 0.35), I^2 = 3.5%
Heterogeneity: Tau^2 (REML[b]) = 0.00; Chi^2 = 11.22, df = 11 (P = 0.43); I^2 = 0%

Footnotes
[a]CI calculated by Wald-type method.
[b]Tau^2 calculated by Restricted Maximum-Likelihood method.
[c]Assumptions: mean = median; SD calculated by dividing the difference between the upper and lower limits of the IQR by 1.35.

Figure S7C: Forest plot presenting the HR_{max} subgroup analysis based on sex distribution.

Study or Subgroup	MD	SE	Weight	Mean difference IV, Random, 95% CI [bpm]
**5.4.1 Mixed**				
Csulak et al. (longitudinal)	-3.2	3.815476	2.6%	-3.20 [-10.68 , 4.28]
Śliż et al.	-0.96	2.024382	9.1%	-0.96 [-4.93 , 3.01]
Aldossari et al.	-0.81	0.862436	50.3%	-0.81 [-2.50 , 0.88]
Csulak et al.	-0.5	3.422086	3.2%	-0.50 [-7.21 , 6.21]
Moulson et al.	0	2.405351	6.5%	0.00 [-4.71 , 4.71]
Bruzzese et al.	3.4	2.576238	5.6%	3.40 [-1.65 , 8.45]
**Subtotal (Wald[a])**			**77.4%**	**-0.52 [-1.88 , 0.84]**
Test for overall effect: Z = 0.75 (P = 0.46)				
Heterogeneity: Tau² (REML[b]) = 0.00; Chi² = 3.02, df = 5 (P = 0.70); I² = 0%				
**5.4.2 Not reported**				
Parpa et al.	1.34	2.407467	6.5%	1.34 [-3.38 , 6.06]
Komici et al.[c]	9	6.555026	0.9%	9.00 [-3.85 , 21.85]
**Subtotal (Wald[a])**			**7.3%**	**2.74 [-3.06 , 8.55]**
Test for overall effect: Z = 0.93 (P = 0.35)				
Heterogeneity: Tau² (REML[b]) = 4.96; Chi² = 1.20, df = 1 (P = 0.27); I² = 17%				
**5.4.3 Only male**				
Stavrou et al.	-5	2.596151	5.6%	-5.00 [-10.09 , 0.09]
Stojmenović et al.	0.93	2.117791	8.3%	0.93 [-3.22 , 5.08]
Fikenzer et al.	7	7.28869	0.7%	7.00 [-7.29 , 21.29]
Fikenzer et al. (longitudinal)	7	7.254309	0.7%	7.00 [-7.22 , 21.22]
**Subtotal (Wald[a])**			**15.3%**	**0.06 [-5.14 , 5.27]**
Test for overall effect: Z = 0.02 (P = 0.98)				
Heterogeneity: Tau² (REML[b]) = 12.46; Chi² = 5.58, df = 3 (P = 0.13); I² = 49%				
**Total (Wald[a])**			**100.0%**	**-0.34 [-1.54 , 0.86]**

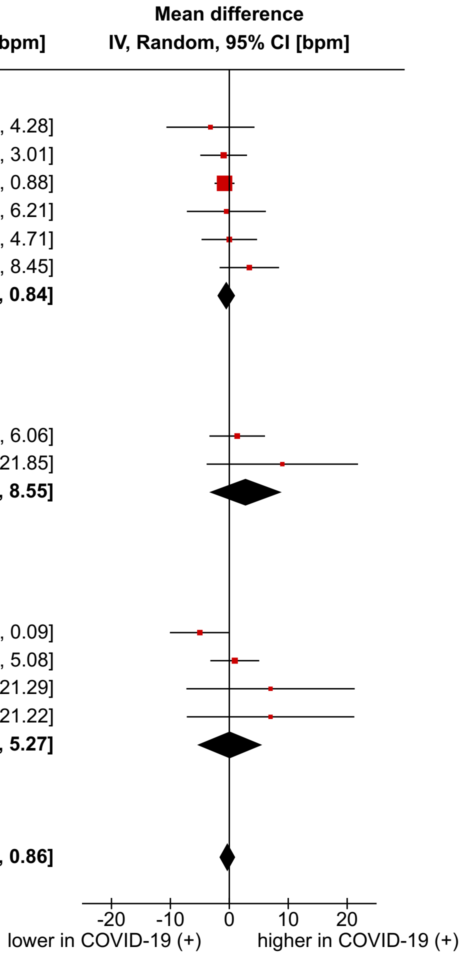

Test for overall effect: Z = 0.55 (P = 0.58)
Test for subgroup differences: Chi² = 1.17, df = 2 (P = 0.56), I² = 0%
Heterogeneity: Tau² (REML[b]) = 0.00; Chi² = 11.22, df = 11 (P = 0.43); I² = 0%

Footnotes

[a]CI calculated by Wald-type method.
[b]Tau² calculated by Restricted Maximum-Likelihood method.
[c]Assumptions: mean = median; SD calculated by dividing the difference between the upper and lower limits of the IQR by 1.35.

Figure S7D: Forest plot presenting the HR_{max} subgroup analysis based on comparison type.

Study or Subgroup	MD	SE	Weight	Mean difference IV, Random, 95% CI [bpm]
**5.5.1 Post-COVID athletes vs. healthy reference**				
Stavrou et al.	-5	2.596151	5.6%	-5.00 [-10.09 , 0.09]
Aldossari et al.	-0.81	0.862436	50.3%	-0.81 [-2.50 , 0.88]
Csulak et al.	-0.5	3.422086	3.2%	-0.50 [-7.21 , 6.21]
Moulson et al.	0	2.405351	6.5%	0.00 [-4.71 , 4.71]
Fikenzer et al.	7	7.28869	0.7%	7.00 [-7.29 , 21.29]
Komici et al.[a]	9	6.555026	0.9%	9.00 [-3.85 , 21.85]
**Subtotal (Wald[b])**			**67.1%**	**-0.85 [-2.32 , 0.61]**
Test for overall effect: Z = 1.14 (P = 0.25)				
Heterogeneity: Tau^2 (REML[c]) = 0.00; Chi^2 = 6.11, df = 5 (P = 0.30); I^2 = 0%				
**5.5.2 Post-COVID athletes vs. the same athletes before infection**				
Csulak et al. (longitudinal)	-3.2	3.815476	2.6%	-3.20 [-10.68 , 4.28]
Śliż et al.	-0.96	2.024382	9.1%	-0.96 [-4.93 , 3.01]
Stojmenović et al.	0.93	2.117791	8.3%	0.93 [-3.22 , 5.08]
Parpa et al.	1.34	2.407467	6.5%	1.34 [-3.38 , 6.06]
Bruzzese et al.	3.4	2.576238	5.6%	3.40 [-1.65 , 8.45]
Fikenzer et al. (longitudinal)	7	7.254309	0.7%	7.00 [-7.22 , 21.22]
**Subtotal (Wald[b])**			**32.9%**	**0.72 [-1.37 , 2.81]**
Test for overall effect: Z = 0.67 (P = 0.50)				
Heterogeneity: Tau^2 (REML[c]) = 0.00; Chi^2 = 3.65, df = 5 (P = 0.60); I^2 = 0%				
**Total (Wald[b])**			**100.0%**	**-0.34 [-1.54 , 0.86]**

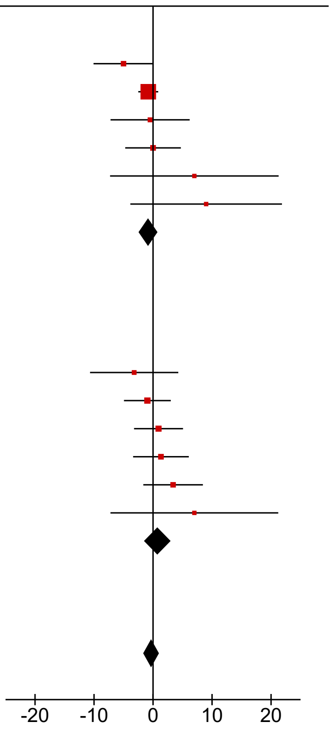

Test for overall effect: Z = 0.55 (P = 0.58)
Test for subgroup differences: Chi^2 = 1.46, df = 1 (P = 0.23), I^2 = 31.3%
Heterogeneity: Tau^2 (REML[c]) = 0.00; Chi^2 = 11.22, df = 11 (P = 0.43); I^2 = 0%

Footnotes

[a]Assumptions: mean = median; SD calculated by dividing the difference between the upper and lower limits of the IQR by 1.35.

[b]CI calculated by Wald-type method.

[c]Tau^2 calculated by Restricted Maximum-Likelihood method.

Figure S7E: Forest plot presenting the HR_{max} subgroup analysis based on the presence of symptoms in athletes during COVID-19 infection.

Study or Subgroup	MD	SE	Weight	Mean difference IV, Random, 95% CI [bpm]
**5.6.1 Mixed**				
Csulak et al. (longitudinal)	-3.2	3.815476	2.6%	-3.20 [-10.68 , 4.28]
Csulak et al.	-0.5	3.422086	3.2%	-0.50 [-7.21 , 6.21]
Bruzzese et al.	3.4	2.576238	5.6%	3.40 [-1.65 , 8.45]
Fikenzer et al.	7	7.28869	0.7%	7.00 [-7.29 , 21.29]
Fikenzer et al. (longitudinal)	7	7.254309	0.7%	7.00 [-7.22 , 21.22]
Komici et al.[a]	9	6.555026	0.9%	9.00 [-3.85 , 21.85]
**Subtotal (Wald[b])**			**13.7%**	**1.98 [-1.26 , 5.22]**
Test for overall effect: Z = 1.20 (P = 0.23)				
Heterogeneity: Tau² (REML[c]) = 0.00; Chi² = 4.77, df = 5 (P = 0.44); I² = 0%				
**5.6.2 Only symptomatic**				
Moulson et al.	0	2.405351	6.5%	0.00 [-4.71 , 4.71]
Parpa et al.	1.34	2.407467	6.5%	1.34 [-3.38 , 6.06]
**Subtotal (Wald[b])**			**12.9%**	**0.67 [-2.67 , 4.00]**
Test for overall effect: Z = 0.39 (P = 0.69)				
Heterogeneity: Tau² (REML[c]) = 0.00; Chi² = 0.16, df = 1 (P = 0.69); I² = 0%				
**5.6.3 Not reported**				
Stavrou et al.	-5	2.596151	5.6%	-5.00 [-10.09 , 0.09]
Śliż et al.	-0.96	2.024382	9.1%	-0.96 [-4.93 , 3.01]
Aldossari et al.	-0.81	0.862436	50.3%	-0.81 [-2.50 , 0.88]
Stojmenović et al.	0.93	2.117791	8.3%	0.93 [-3.22 , 5.08]
**Subtotal (Wald[b])**			**73.4%**	**-0.95 [-2.35 , 0.45]**
Test for overall effect: Z = 1.33 (P = 0.18)				
Heterogeneity: Tau² (REML[c]) = 0.00; Chi² = 3.25, df = 3 (P = 0.35); I² = 0%				
**Total (Wald[b])**			**100.0%**	**-0.34 [-1.54 , 0.86]**

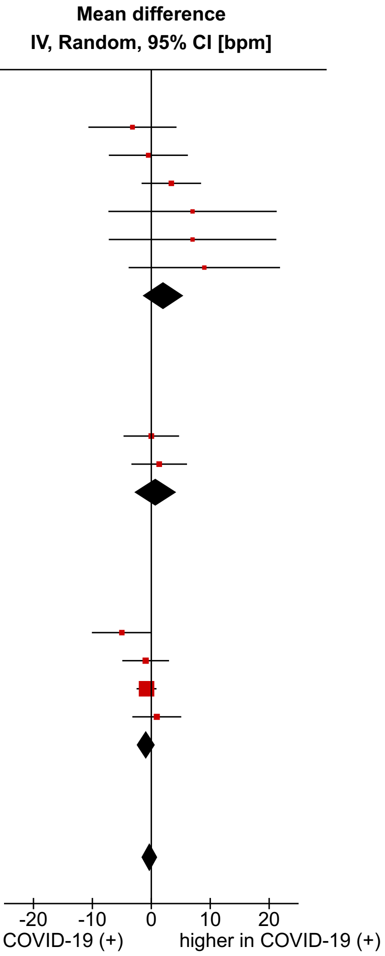

Test for overall effect: Z = 0.55 (P = 0.58)
Test for subgroup differences: Chi² = 3.04, df = 2 (P = 0.22), I² = 34.3%
Heterogeneity: Tau² (REML[c]) = 0.00; Chi² = 11.22, df = 11 (P = 0.43); I² = 0%

Footnotes

[a]Assumptions: mean = median; SD calculated by dividing the difference between the upper and lower limits of the IQR by 1.35.
[b]CI calculated by Wald-type method.
[c]Tau² calculated by Restricted Maximum-Likelihood method.

Figure S7F: Forest plot presenting the HR_{max} subgroup analysis based on time elapsed from COVID-19 infection to CPET.

Study or Subgroup	MD	SE	Weight	Mean difference IV, Random, 95% CI [bpm]
**5.7.1 Not reported**				
**Subtotal**				**Not estimable**
Test for overall effect: Not applicable				
Heterogeneity: Not applicable				
**5.7.2 Up to 1 month after infection**				
Stavrou et al.	-5	2.596151	5.6%	-5.00 [-10.09 , 0.09]
Csulak et al. (longitudinal)	-3.2	3.815476	2.6%	-3.20 [-10.68 , 4.28]
Csulak et al.	-0.5	3.422086	3.2%	-0.50 [-7.21 , 6.21]
Stojmenović et al.	0.93	2.117791	8.3%	0.93 [-3.22 , 5.08]
Bruzzese et al.	3.4	2.576238	5.6%	3.40 [-1.65 , 8.45]
Fikenzer et al.	7	7.28869	0.7%	7.00 [-7.29 , 21.29]
Fikenzer et al. (longitudinal)	7	7.254309	0.7%	7.00 [-7.22 , 21.22]
Komici et al.[a]	9	6.555026	0.9%	9.00 [-3.85 , 21.85]
**Subtotal (Wald[b])**			**27.6%**	**0.47 [-2.52 , 3.45]**
Test for overall effect: Z = 0.31 (P = 0.76)				
Heterogeneity: Tau² (REML[c]) = 5.37; Chi² = 10.06, df = 7 (P = 0.19); I² = 31%				
**5.7.3 More than 1 month after infection**				
Śliż et al.	-0.96	2.024382	9.1%	-0.96 [-4.93 , 3.01]
Aldossari et al.	-0.81	0.862436	50.3%	-0.81 [-2.50 , 0.88]
Moulson et al.	0	2.405351	6.5%	0.00 [-4.71 , 4.71]
Parpa et al.	1.34	2.407467	6.5%	1.34 [-3.38 , 6.06]
**Subtotal (Wald[b])**			**72.4%**	**-0.56 [-1.97 , 0.84]**
Test for overall effect: Z = 0.79 (P = 0.43)				
Heterogeneity: Tau² (REML[c]) = 0.00; Chi² = 0.80, df = 3 (P = 0.85); I² = 0%				
**Total (Wald[b])**			**100.0%**	**-0.34 [-1.54 , 0.86]**

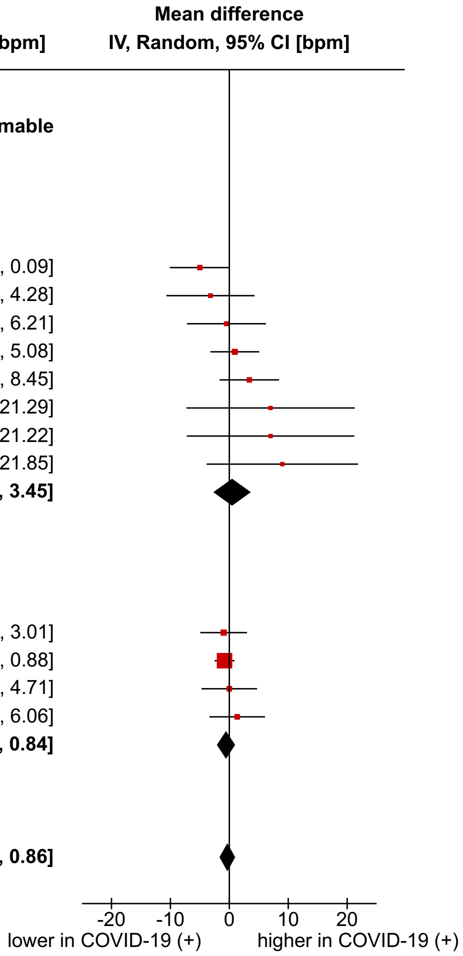

Test for overall effect: Z = 0.55 (P = 0.58)
Test for subgroup differences: Chi² = 0.38, df = 1 (P = 0.54), I² = 0%
Heterogeneity: Tau² (REML[c]) = 0.00; Chi² = 11.22, df = 11 (P = 0.43); I² = 0%

Footnotes

[a]Assumptions: mean = median; SD calculated by dividing the difference between the upper and lower limits of the IQR by 1.35.
[b]CI calculated by Wald-type method.
[c]Tau² calculated by Restricted Maximum-Likelihood method.

Figure S8A: Forest plot presenting the VE_{max} subgroup analysis based on competition level.

Study or Subgroup	MD	SE	Weight	Mean difference IV, Random, 95% CI [L/min]
**6.2.1 Recreational**				
Aldossari et al.	-12.21	9.580815	7.0%	-12.21 [-30.99 , 6.57]
**Subtotal**			**7.0%**	**-12.21 [-30.99 , 6.57]**
Test for overall effect: Z = 1.27 (P = 0.20)				
Heterogeneity: Not applicable				
**6.2.2 Competitive**				
Fikenzer et al.	-25	12.534951	4.1%	-25.00 [-49.57 , -0.43]
Fikenzer et al. (longitudinal)	-19	8.01561	9.9%	-19.00 [-34.71 , -3.29]
Roczniok et al.[a]	-8.83	6.242563	16.4%	-8.83 [-21.07 , 3.41]
Komici et al.[b]	-8.6	5.453215	21.5%	-8.60 [-19.29 , 2.09]
Csulak et al.[c]	7.4	12.07515	4.4%	7.40 [-16.27 , 31.07]
Csulak et al. (longitudinal)[c]	9	11.422096	4.9%	9.00 [-13.39 , 31.39]
**Subtotal (Wald[d])**			**61.1%**	**-8.89 [-15.22 , -2.55]**
Test for overall effect: Z = 2.75 (P = 0.006)				
Heterogeneity: Tau^2 (REML[e]) = 0.00; Chi^2 = 7.52, df = 5 (P = 0.18); I^2 = 0%				
**6.2.3 Mixed**				
Moulson et al.	-8	9.019819	7.8%	-8.00 [-25.68 , 9.68]
Śliż et al.	-4.49	5.142296	24.1%	-4.49 [-14.57 , 5.59]
**Subtotal (Wald[d])**			**32.0%**	**-5.35 [-14.11 , 3.40]**
Test for overall effect: Z = 1.20 (P = 0.23)				
Heterogeneity: Tau^2 (REML[e]) = 0.00; Chi^2 = 0.11, df = 1 (P = 0.74); I^2 = 0%				
**Total (Wald[d])**			**100.0%**	**-7.99 [-12.94 , -3.04]**

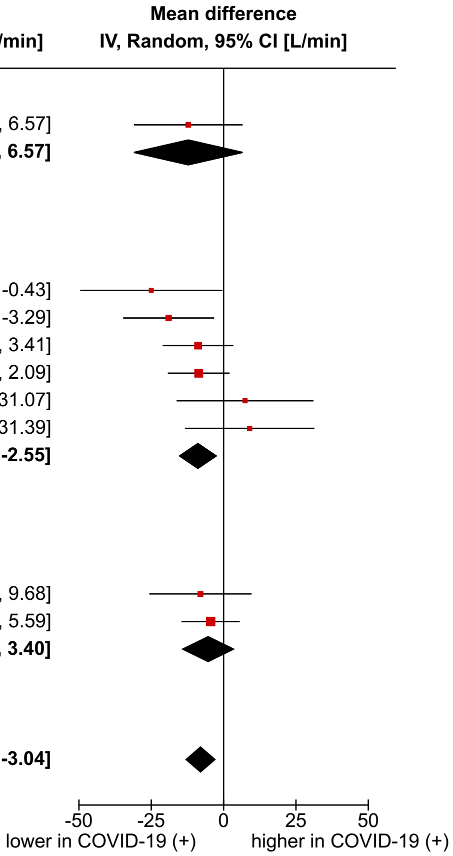

Test for overall effect: Z = 3.16 (P = 0.002)
Test for subgroup differences: Chi^2 = 0.62, df = 2 (P = 0.73), I^2 = 0%
Heterogeneity: Tau^2 (REML[e]) = 0.00; Chi^2 = 8.25, df = 8 (P = 0.41); I^2 = 0%

Footnotes

[a]Combined mean±SD for COVID-19(+) athletes from weighted mean and pooled sample SD using groups with and without symptoms.
[b]Assumptions: mean = median; SD calculated by dividing the difference between the upper and lower limits of the IQR by 1.35.
[c]Mean and SD calculated for the whole cohorts based on values for male and female.
[d]CI calculated by Wald-type method.
[e]Tau^2 calculated by Restricted Maximum-Likelihood method.

Figure S8B: Forest plot presenting the VE_{max} subgroup analysis based on CPET modality.

Study or Subgroup	MD	SE	Weight	Mean difference IV, Random, 95% CI [L/min]
**6.3.1 Cycle ergometer**				
Fikenzer et al.	-25	12.534951	4.1%	-25.00 [-49.57 , -0.43]
Fikenzer et al. (longitudinal)	-19	8.01561	9.9%	-19.00 [-34.71 , -3.29]
Roczniok et al.[a]	-8.83	6.242563	16.4%	-8.83 [-21.07 , 3.41]
**Subtotal (Wald[b])**			**30.4%**	**-14.47 [-23.76 , -5.17]**
Test for overall effect: Z = 3.05 (P = 0.002)				
Heterogeneity: Tau^2 (REML[c]) = 3.62; Chi^2 = 1.84, df = 2 (P = 0.40); I^2 = 5%				
**6.3.2 Mixed**				
Moulson et al.	-8	9.019819	7.8%	-8.00 [-25.68 , 9.68]
Śliż et al.	-4.49	5.142296	24.1%	-4.49 [-14.57 , 5.59]
**Subtotal (Wald[b])**			**32.0%**	**-5.35 [-14.11 , 3.40]**
Test for overall effect: Z = 1.20 (P = 0.23)				
Heterogeneity: Tau^2 (REML[c]) = 0.00; Chi^2 = 0.11, df = 1 (P = 0.74); I^2 = 0%				
**6.3.3 Treadmill**				
Aldossari et al.	-12.21	9.580815	7.0%	-12.21 [-30.99 , 6.57]
Komici et al.[d]	-8.6	5.453215	21.5%	-8.60 [-19.29 , 2.09]
Csulak et al.[e]	7.4	12.07515	4.4%	7.40 [-16.27 , 31.07]
Csulak et al. (longitudinal)[e]	9	11.422096	4.9%	9.00 [-13.39 , 31.39]
**Subtotal (Wald[b])**			**37.7%**	**-4.74 [-13.45 , 3.96]**
Test for overall effect: Z = 1.07 (P = 0.29)				
Heterogeneity: Tau^2 (REML[c]) = 7.46; Chi^2 = 3.56, df = 3 (P = 0.31); I^2 = 8%				
**Total (Wald[b])**			**100.0%**	**-7.99 [-12.94 , -3.04]**
Test for overall effect: Z = 3.16 (P = 0.002)				
Test for subgroup differences: Chi^2 = 2.75, df = 2 (P = 0.25), I^2 = 27.2%				
Heterogeneity: Tau^2 (REML[c]) = 0.00; Chi^2 = 8.25, df = 8 (P = 0.41); I^2 = 0%				

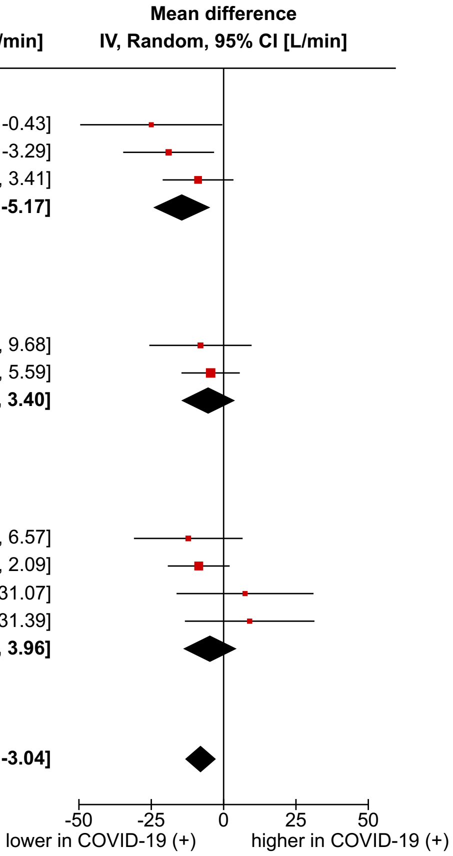

Footnotes

[a]Combined mean±SD for COVID-19(+) athletes from weighted mean and pooled sample SD using groups with and without symptoms.
[b]CI calculated by Wald-type method.
[c]Tau^2 calculated by Restricted Maximum-Likelihood method.
[d]Assumptions: mean = median; SD calculated by dividing the difference between the upper and lower limits of the IQR by 1.35.
[e]Mean and SD calculated for the whole cohorts based on values for male and female.

Figure S8C: Forest plot presenting the VE_{max} subgroup analysis based on sex distribution.

Study or Subgroup	MD	SE	Weight	Mean difference IV, Random, 95% CI [L/min]
**6.4.1 Mixed**				
Aldossari et al.	-12.21	9.580815	7.0%	-12.21 [-30.99 , 6.57]
Moulson et al.	-8	9.019819	7.8%	-8.00 [-25.68 , 9.68]
Śliż et al.	-4.49	5.142296	24.1%	-4.49 [-14.57 , 5.59]
Csulak et al.[a]	7.4	12.07515	4.4%	7.40 [-16.27 , 31.07]
Csulak et al. (longitudinal)[a]	9	11.422096	4.9%	9.00 [-13.39 , 31.39]
**Subtotal (Wald[b])**			**48.2%**	**-3.73 [-10.86 , 3.41]**
Test for overall effect: Z = 1.02 (P = 0.31)				
Heterogeneity: Tau^2 (REML[c]) = 0.00; Chi^2 = 3.12, df = 4 (P = 0.54); I^2 = 0%				
**6.4.2 Not reported**				
Roczniok et al.[d]	-8.83	6.242563	16.4%	-8.83 [-21.07 , 3.41]
Komici et al.[e]	-8.6	5.453215	21.5%	-8.60 [-19.29 , 2.09]
**Subtotal (Wald[b])**			**37.8%**	**-8.70 [-16.75 , -0.65]**
Test for overall effect: Z = 2.12 (P = 0.03)				
Heterogeneity: Tau^2 (REML[c]) = 0.00; Chi^2 = 0.00, df = 1 (P = 0.98); I^2 = 0%				
**6.4.3 Only male**				
Fikenzer et al.	-25	12.534951	4.1%	-25.00 [-49.57 , -0.43]
Fikenzer et al. (longitudinal)	-19	8.01561	9.9%	-19.00 [-34.71 , -3.29]
**Subtotal (Wald[b])**			**14.0%**	**-20.74 [-33.98 , -7.51]**
Test for overall effect: Z = 3.07 (P = 0.002)				
Heterogeneity: Tau^2 (REML[c]) = 0.00; Chi^2 = 0.16, df = 1 (P = 0.69); I^2 = 0%				
**Total (Wald[b])**			**100.0%**	**-7.99 [-12.94 , -3.04]**
Test for overall effect: Z = 3.16 (P = 0.002)				
Test for subgroup differences: Chi^2 = 4.97, df = 2 (P = 0.08), I^2 = 59.7%				
Heterogeneity: Tau^2 (REML[c]) = 0.00; Chi^2 = 8.25, df = 8 (P = 0.41); I^2 = 0%				

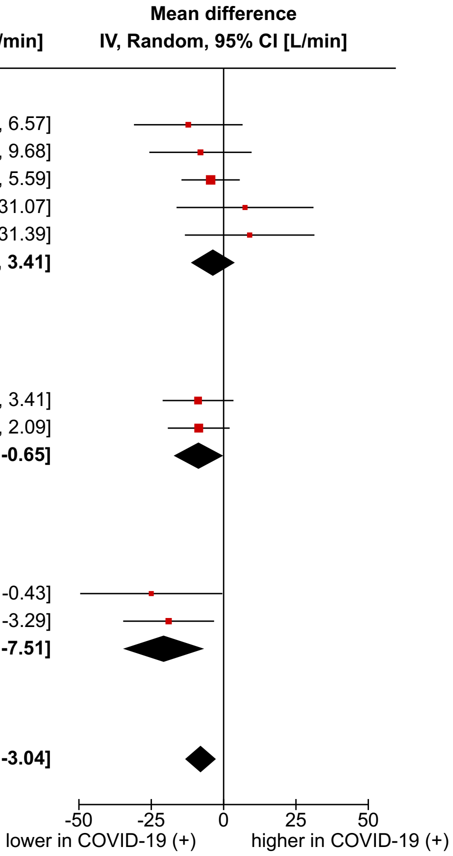

Footnotes

[a]Mean and SD calculated for the whole cohorts based on values for male and female.

[b]CI calculated by Wald-type method.

[c]Tau^2 calculated by Restricted Maximum-Likelihood method.

[d]Combined mean±SD for COVID-19(+) athletes from weighted mean and pooled sample SD using groups with and without symptoms.

[e]Assumptions: mean = median; SD calculated by dividing the difference between the upper and lower limits of the IQR by 1.35.

Figure S8D: Forest plot presenting the VE_{max} subgroup analysis based on comparison type.

Study or Subgroup	MD	SE	Weight	Mean difference IV, Random, 95% CI [L/min]
**6.5.1 Post-COVID athletes vs. healthy reference**				
Fikenzer et al.	-25	12.534951	4.1%	-25.00 [-49.57 , -0.43]
Aldossari et al.	-12.21	9.580815	7.0%	-12.21 [-30.99 , 6.57]
Roczniok et al.[a]	-8.83	6.242563	16.4%	-8.83 [-21.07 , 3.41]
Komici et al.[b]	-8.6	5.453215	21.5%	-8.60 [-19.29 , 2.09]
Moulson et al.	-8	9.019819	7.8%	-8.00 [-25.68 , 9.68]
Csulak et al.[c]	7.4	12.07515	4.4%	7.40 [-16.27 , 31.07]
**Subtotal (Wald[d])**			**61.1%**	**-8.94 [-15.28 , -2.60]**
Test for overall effect: Z = 2.77 (P = 0.006)				
Heterogeneity: Tau² (REML[e]) = 0.00; Chi² = 3.60, df = 5 (P = 0.61); I² = 0%				
**6.5.2 Post-COVID athletes vs. the same athletes before infection**				
Fikenzer et al. (longitudinal)	-19	8.01561	9.9%	-19.00 [-34.71 , -3.29]
Śliż et al.	-4.49	5.142296	24.1%	-4.49 [-14.57 , 5.59]
Csulak et al. (longitudinal)[c]	9	11.422096	4.9%	9.00 [-13.39 , 31.39]
**Subtotal (Wald[d])**			**38.9%**	**-6.24 [-19.59 , 7.12]**
Test for overall effect: Z = 0.92 (P = 0.36)				
Heterogeneity: Tau² (REML[e]) = 77.15; Chi² = 4.43, df = 2 (P = 0.11); I² = 56%				
**Total (Wald[d])**			**100.0%**	**-7.99 [-12.94 , -3.04]**

Mean difference IV, Random, 95% CI [L/min]

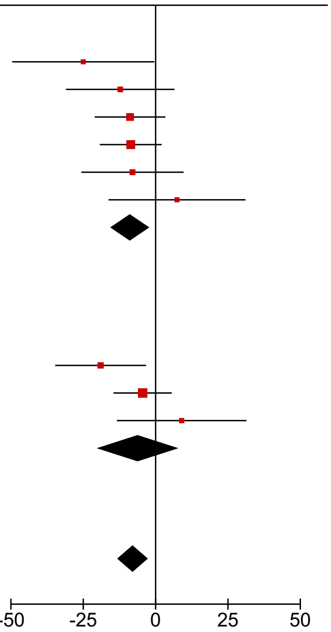

Test for overall effect: Z = 3.16 (P = 0.002)
Test for subgroup differences: Chi² = 0.13, df = 1 (P = 0.72), I² = 0%
Heterogeneity: Tau² (REML[e]) = 0.00; Chi² = 8.25, df = 8 (P = 0.41); I² = 0%

Footnotes

[a]Combined mean±SD for COVID-19(+) athletes from weighted mean and pooled sample SD using groups with and without symptoms.
[b]Assumptions: mean = median; SD calculated by dividing the difference between the upper and lower limits of the IQR by 1.35.
[c]Mean and SD calculated for the whole cohorts based on values for male and female.
[d]CI calculated by Wald-type method.
[e]Tau² calculated by Restricted Maximum-Likelihood method.

Figure S8E: Forest plot presenting the VE_{max} subgroup analysis based on the presence of symptoms in athletes during COVID-19 infection.

Study or Subgroup	MD	SE	Weight	Mean difference IV, Random, 95% CI [L/min]
**6.6.1 Mixed**				
Fikenzer et al.	-25	12.534951	4.1%	-25.00 [-49.57 , -0.43]
Fikenzer et al. (longitudinal)	-19	8.01561	9.9%	-19.00 [-34.71 , -3.29]
Roczniok et al.[a]	-8.83	6.242563	16.4%	-8.83 [-21.07 , 3.41]
Komici et al.[b]	-8.6	5.453215	21.5%	-8.60 [-19.29 , 2.09]
Csulak et al.[c]	7.4	12.07515	4.4%	7.40 [-16.27 , 31.07]
Csulak et al. (longitudinal)[c]	9	11.422096	4.9%	9.00 [-13.39 , 31.39]
**Subtotal (Wald[d])**			**61.1%**	**-8.89 [-15.22 , -2.55]**
Test for overall effect: Z = 2.75 (P = 0.006)				
Heterogeneity: Tau^2 (REML[e]) = 0.00; Chi^2 = 7.52, df = 5 (P = 0.18); I^2 = 0%				
**6.6.2 Only symptomatic**				
Moulson et al.	-8	9.019819	7.8%	-8.00 [-25.68 , 9.68]
**Subtotal**			**7.8%**	**-8.00 [-25.68 , 9.68]**
Test for overall effect: Z = 0.89 (P = 0.38)				
Heterogeneity: Not applicable				
**6.6.3 Not reported**				
Aldossari et al.	-12.21	9.580815	7.0%	-12.21 [-30.99 , 6.57]
Śliż et al.	-4.49	5.142296	24.1%	-4.49 [-14.57 , 5.59]
**Subtotal (Wald[d])**			**31.1%**	**-6.22 [-15.10 , 2.66]**
Test for overall effect: Z = 1.37 (P = 0.17)				
Heterogeneity: Tau^2 (REML[e]) = 0.00; Chi^2 = 0.50, df = 1 (P = 0.48); I^2 = 0%				
**Total (Wald[d])**			**100.0%**	**-7.99 [-12.94 , -3.04]**

Test for overall effect: Z = 3.16 (P = 0.002)
Test for subgroup differences: Chi^2 = 0.23, df = 2 (P = 0.89), I^2 = 0%
Heterogeneity: Tau^2 (REML[e]) = 0.00; Chi^2 = 8.25, df = 8 (P = 0.41); I^2 = 0%

Mean difference IV, Random, 95% CI [L/min]

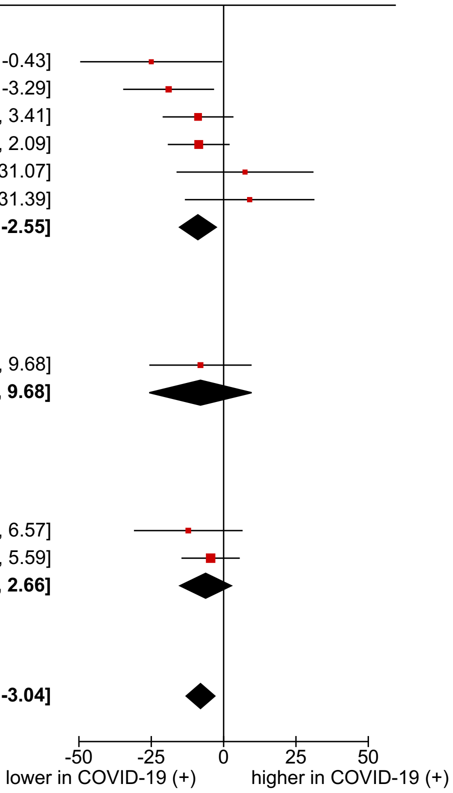

Footnotes

[a]Combined mean±SD for COVID-19(+) athletes from weighted mean and pooled sample SD using groups with and without symptoms.
[b]Assumptions: mean = median; SD calculated by dividing the difference between the upper and lower limits of the IQR by 1.35.
[c]Mean and SD calculated for the whole cohorts based on values for male and female.
[d]CI calculated by Wald-type method.
[e]Tau^2 calculated by Restricted Maximum-Likelihood method.

Figure S8F: Forest plot presenting the VE_{max} subgroup analysis based on time elapsed from COVID-19 infection to CPET.

Study or Subgroup	MD	SE	Weight	Mean difference IV, Random, 95% CI [L/min]
**6.7.1 Not reported**				
**Subtotal**				**Not estimable**
Test for overall effect: Not applicable				
Heterogeneity: Not applicable				
**6.7.2 Up to 1 month after infection**				
Fikenzer et al.	-25	12.534951	4.1%	-25.00 [-49.57 , -0.43]
Fikenzer et al. (longitudinal)	-19	8.01561	9.9%	-19.00 [-34.71 , -3.29]
Komici et al.[a]	-8.6	5.453215	21.5%	-8.60 [-19.29 , 2.09]
Csulak et al.[b]	7.4	12.07515	4.4%	7.40 [-16.27 , 31.07]
Csulak et al. (longitudinal)[b]	9	11.422096	4.9%	9.00 [-13.39 , 31.39]
**Subtotal (Wald[c])**			**44.7%**	**-8.21 [-19.28 , 2.87]**
Test for overall effect: Z = 1.45 (P = 0.15)				
Heterogeneity: Tau^2 (REML[d]) = 70.80; Chi^2 = 7.52, df = 4 (P = 0.11); I^2 = 46%				
**6.7.3 More than 1 month after infection**				
Aldossari et al.	-12.21	9.580815	7.0%	-12.21 [-30.99 , 6.57]
Roczniok et al.[e]	-8.83	6.242563	16.4%	-8.83 [-21.07 , 3.41]
Moulson et al.	-8	9.019819	7.8%	-8.00 [-25.68 , 9.68]
Śliż et al.	-4.49	5.142296	24.1%	-4.49 [-14.57 , 5.59]
**Subtotal (Wald[c])**			**55.3%**	**-7.24 [-13.90 , -0.59]**
Test for overall effect: Z = 2.13 (P = 0.03)				
Heterogeneity: Tau^2 (REML[d]) = 0.00; Chi^2 = 0.63, df = 3 (P = 0.89); I^2 = 0%				
**Total (Wald[c])**			**100.0%**	**-7.99 [-12.94 , -3.04]**

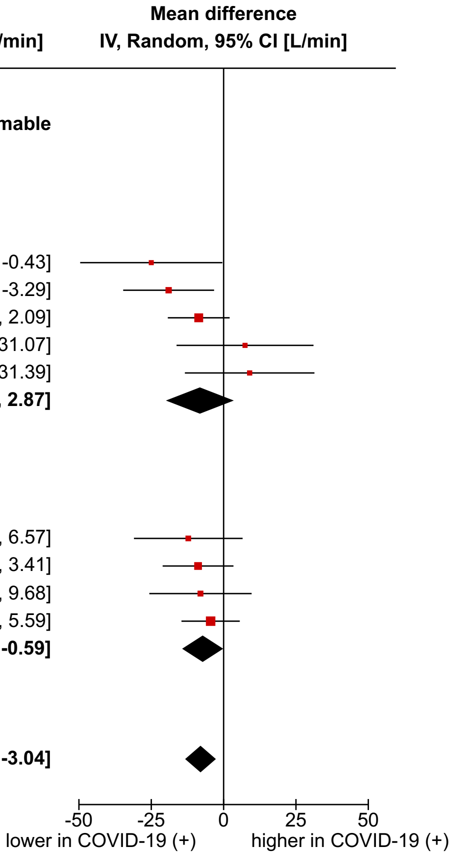

Test for overall effect: Z = 3.16 (P = 0.002)
Test for subgroup differences: Chi^2 = 0.02, df = 1 (P = 0.88), I^2 = 0%
Heterogeneity: Tau^2 (REML[d]) = 0.00; Chi^2 = 8.25, df = 8 (P = 0.41); I^2 = 0%

Footnotes

[a]Assumptions: mean = median; SD calculated by dividing the difference between the upper and lower limits of the IQR by 1.35.
[b]Mean and SD calculated for the whole cohorts based on values for male and female.
[c]CI calculated by Wald-type method.
[d]Tau^2 calculated by Restricted Maximum-Likelihood method.
[e]Combined mean±SD for COVID-19(+) athletes from weighted mean and pooled sample SD using groups with and without symptoms.

Figure S9: Funnel plot assessing the publication bias for relative VO_{2max}.

The dotted line represents the effect estimate.

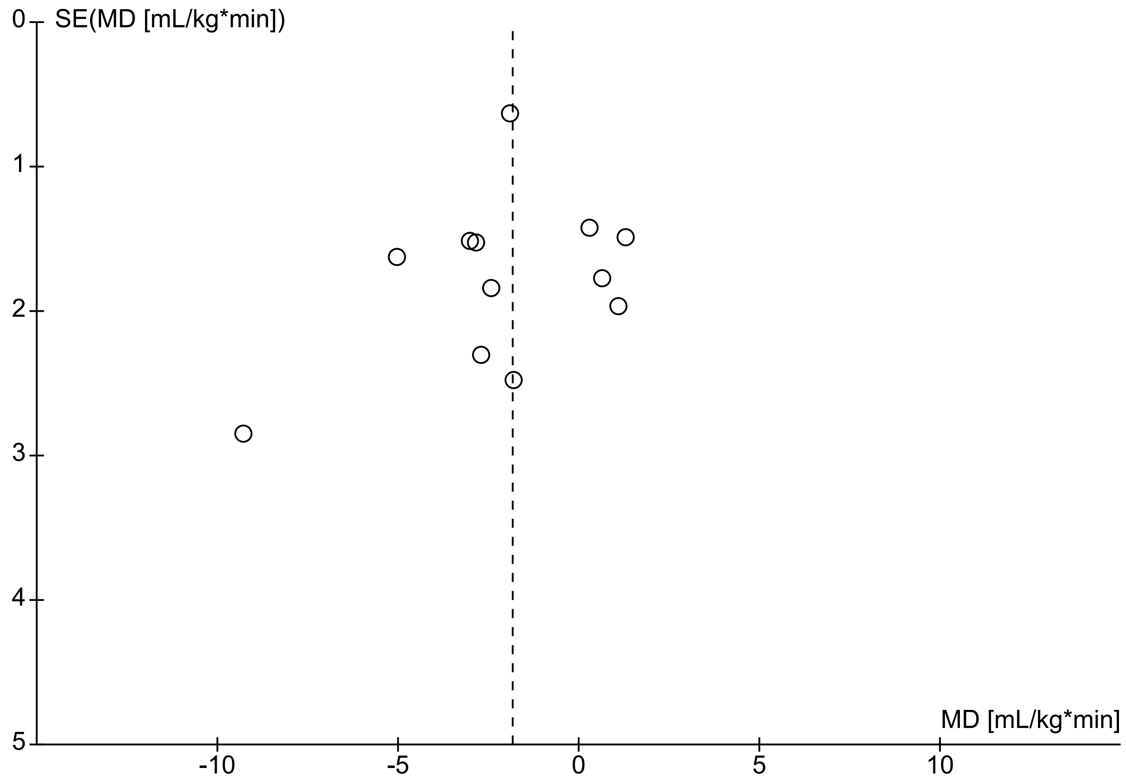

Figure S10: Funnel plot assessing the publication bias for absolute VO_{2max}.

The dotted line represents the effect estimate.

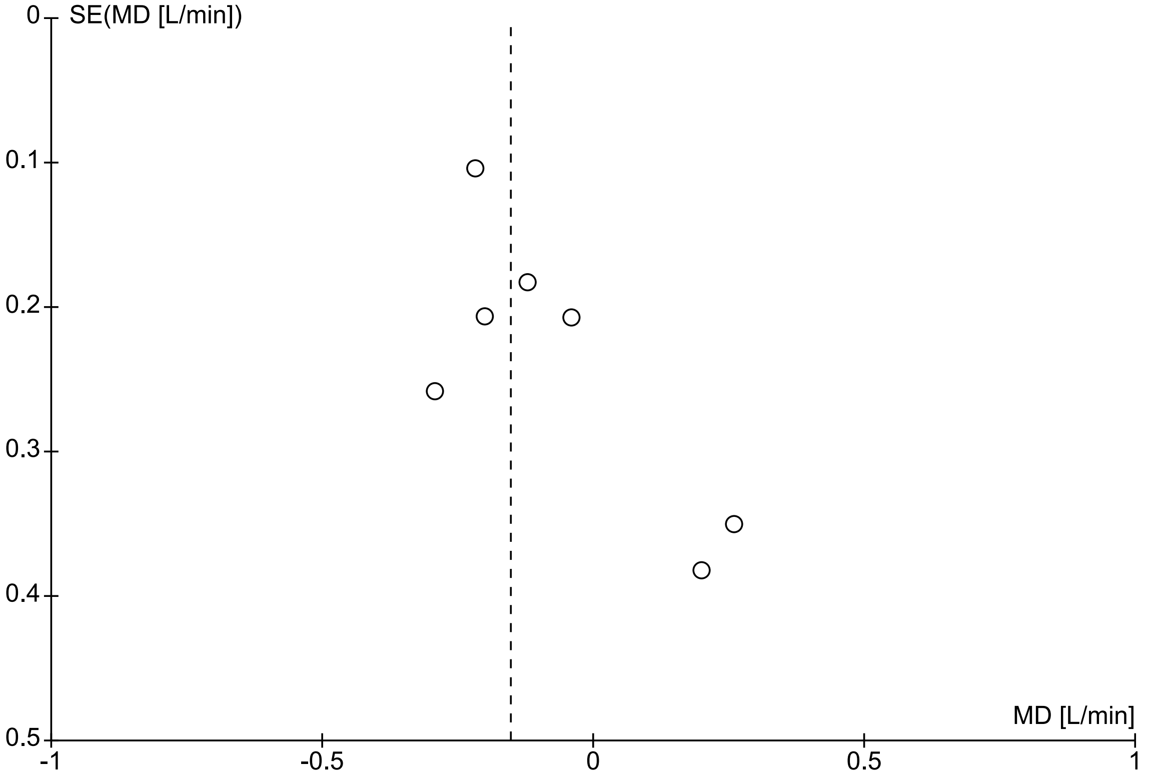

Figure S11: Funnel plot assessing the publication bias for HR_{max}.

The dotted line represents the effect estimate.

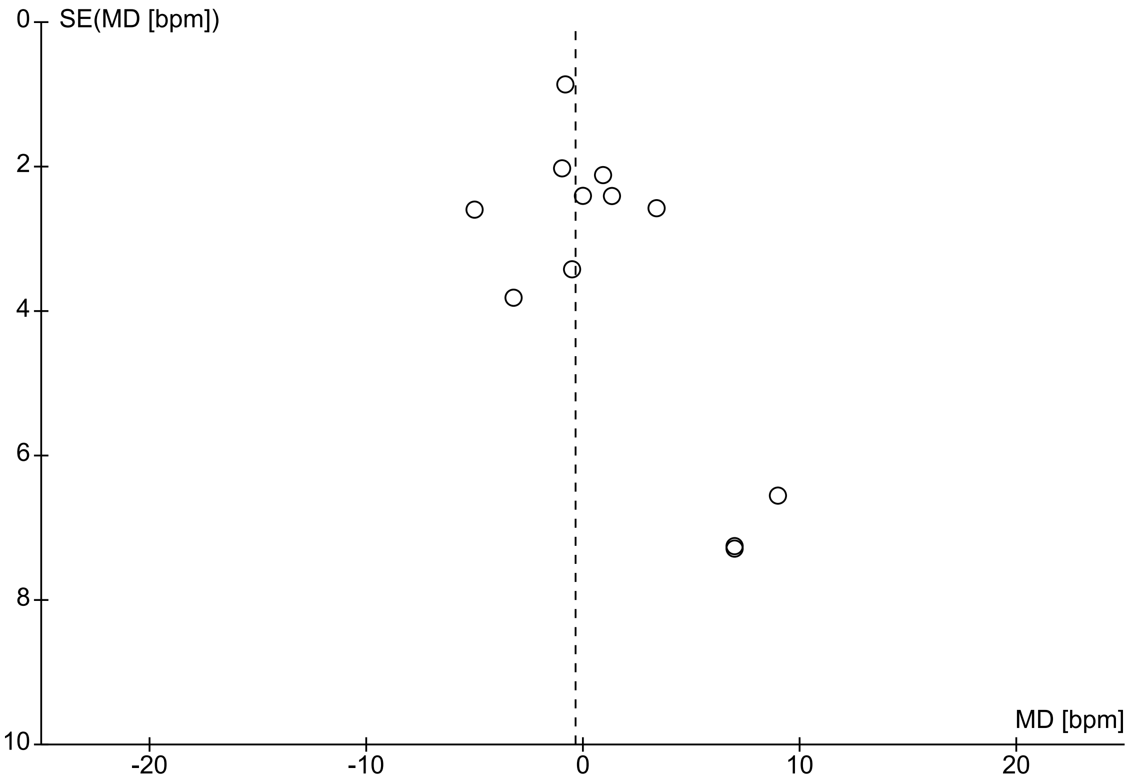

Figure S12: Funnel plot assessing the publication bias for VE_{max}.

The dotted line represents the effect estimate.

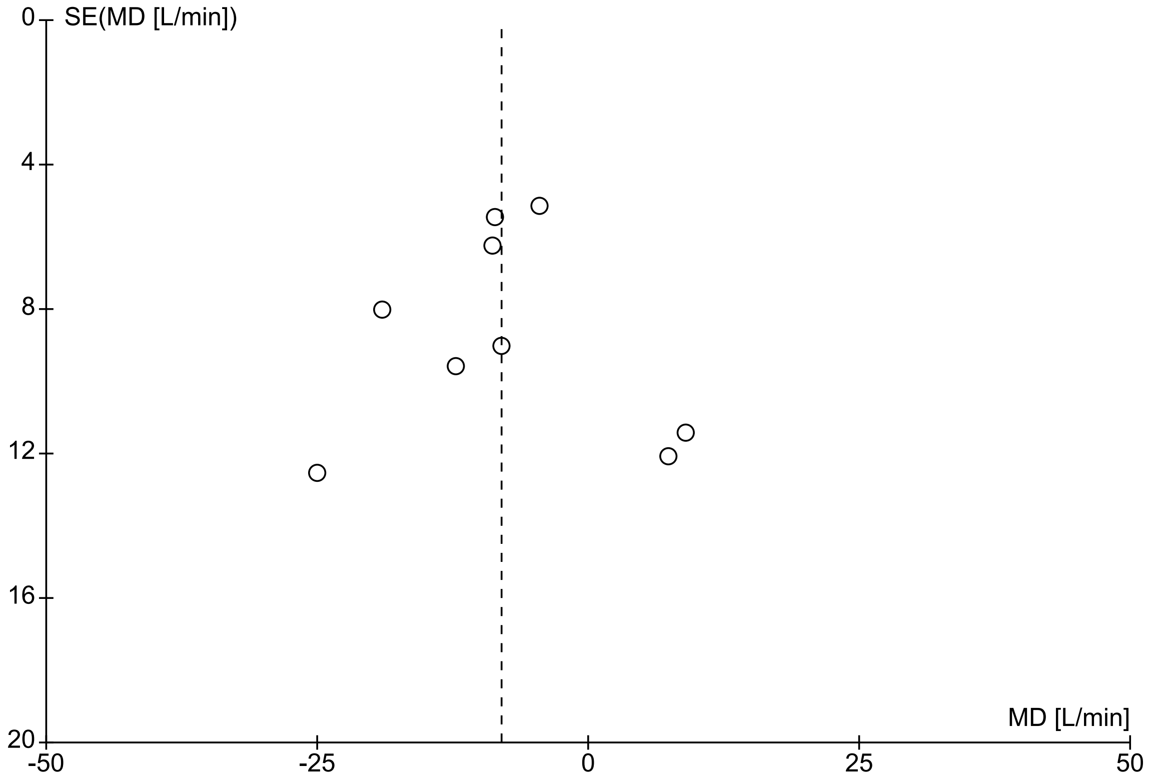

Figure S13: Risk of bias assessment of included studies.

	Eligibility & Sampling	Population & Setting Reporting	Exposure Ascertainment	Outcome Definition / Diagnostic Criteria	Confounders—Identification	Confounders—Control/Adjustment	Outcome Measurement Validity/Reliability	Statistical Methods Appropriateness
Aldossari et al.	−	+	+	−	+	−	−	+
Bruzzese et al.	−	−	+	−	−	−	+	+
Csulak et al.	−	+	+	+	+	+	+	+
Csulak et al. (longitudinal)	−	+	+	+	+	+	+	+
Fikenzer et al.	−	+	−	−	+	−	+	+
Fikenzer et al. (longitudinal)	−	+	−	−	+	−	+	+
Keller et al.	+	−	−	−	+	−	−	−
Komici et al.	+	+	+	+	+	−	+	+
Moulson et al.	+	+	−	+	+	+	+	+
Parpa et al.	+	−	+	+	−	−	+	+
Roczniok et al.	+	−	−	−	−	−	+	+
Śliż et al.	+	+	+	+	+	−	+	+
Stavrou et al.	+	+	−	−	+	−	+	+
Stojmenović et al.	−	−	+	−	−	−	+	+

Note: A red circle means high risk of bias, while a green circle means low risk of bias.

Table S3: The summary of findings in terms of the certainty of evidence regarding relative VO_{2max}, absolute VO_{2max}, HR_{max}, and VE_{max}.

Certainty assessment							№ of patients		Effect		Certainty	Importance
№ of studies	Study design	Risk of bias	Inconsistency	Indirectness	Imprecision	Other considerations	COVID-19 (+)	COVID-19 (-)	Relative (95% CI)	Absolute (95% CI)		
**Relative VO2max**												
12	non-randomized studies	serious	serious	not serious	not serious	publication bias strongly suspected	415	1303	-	MD **1.83 lower** (3.16 lower to 0.49 lower)	⊕◯◯◯ Very low	IMPORTANT
**Absolute VO2max**												
7	non-randomized studies	serious	serious	not serious	not serious	publication bias strongly suspected	124	159	-	MD **0.15 lower** (0.29 lower to 0.01 lower)	⊕◯◯◯ Very low	IMPORTANT
**HRmax**												
12	non-randomized studies	serious	serious	not serious	not serious	publication bias strongly suspected	237	259	-	MD **0.34 lower** (1.54 lower to 0.86 higher)	⊕◯◯◯ Very low	NOT IMPORTANT
**VEmax**												
9	non-randomized studies	serious	serious	not serious	not serious	none	196	194	-	MD **7.99 lower** (12.94 lower to 3.04 lower)	⊕◯◯◯ Very low	IMPORTANT

Supplementary references

1. Aldossari, H.A.; Alsubaiei, M.E.; Al Humoud, S.Y.; Aldossari, H.A.; Shanb, A.A. Impact of Covid-19 on maximal oxygen uptake, physical activity, and fatigue in Saudi recreational athletes: A cross-sectional study. Acta Biomed. 2025, 96. https://doi.org/10.23750/abm.v96i3.17107.

2. Bruzzese, M.F.; Bazán, N.E.; Echandía, N.A.; Garcia, G.C. Evaluation of maximum oxygen consumption pre and post COVID-19 in elite soccer player from Argentina. Arch. Med. Deporte 2023, 40, 217–221. https://doi.org/10.18176/archmeddeporte.00138.

3. Csulak, E.; Petrov, Á.; Kováts, T.; Tokodi, M.; Lakatos, B.; Kovács, A.; Staub, L.; Suhai, F.I.; Szabó, E.L.; Dohy, Z.; et al. The impact of covid-19 on the preparation for the tokyo olympics: A comprehensive performance assessment of top swimmers. Int. J. Environ. Res. Public. Health 2021, 18, 9770. https://doi.org/10.3390/ijerph18189770.

4. Fikenzer, S.; Kogel, A.; Pietsch, C.; Lavall, D.; Stöbe, S.; Rudolph, U.; Laufs, U.; Hepp, P.; Hagendorff, A. SARS-CoV2 infection: Functional and morphological cardiopulmonary changes in elite handball players. Sci. Rep. 2021, 11, 17798. https://doi.org/10.1038/s41598-021-97120-x.

5. Keller, K.; Friedrich, O.; Treiber, J.; Quermann, A.; Friedmann-Bette, B. Former SARS-CoV-2 Infection Was Related to Decreased VO2 Peak and Exercise Hypertension in Athletes. Diagnostics 2023, 13, 1792. https://doi.org/10.3390/diagnostics13101792.

6. Komici, K.; Bianco, A.; Perrotta, F.; Dello Iacono, A.; Bencivenga, L.; D'Agnano, V.; Rocca, A.; Bianco, A.; Rengo, G.; Guerra, G. Clinical Characteristics, Exercise Capacity and Pulmonary Function in Post-COVID-19 Competitive Athletes. J. Clin. Med. 2021, 10, 3053. https://doi.org/10.3390/jcm10143053.

7. Moulson, N.; Gustus, S.K.; Scirica, C.; Petek, B.J.; Vanatta, C.; Churchill, T.W.; Guseh, J.S.; Baggish, A.; Wasfy, M.M. Diagnostic evaluation and cardiopulmonary exercise test findings in young athletes with persistent symptoms following COVID-19. Br. J. Sports Med. 2022, 56, 927–932. https://doi.org/10.1136/bjsports-2021-105157.

8. Parpa, K.; Michaelides, M. Aerobic capacity of professional soccer players before and after COVID-19 infection. Sci. Rep. 2022, 12, 11850. https://doi.org/10.1038/s41598-022-16031-7.

9. Roczniok, R.; Terbalyan, A.; Pietraszewski, P.; Mikrut, G.; Zielonka, H.; Stastny, P.; Swinarew, A.; Manilewska, D.; Ornowski, K.; Jabłoński, T.; et al. Impact of SARS-CoV-2 on Aerobic and Anaerobic Capacity in Professional Ice Hockey Players. J. Clin. Med. 2025, 14, 3478. https://doi.org/10.3390/jcm14103478.

10. Stavrou, V.T.; Kyriaki, A.; Vavougios, G.D.; Fatouros, I.G.; Metsios, G.S.; Kalabakas, K.; Karagiannis, D.; Daniil, Z.; Gourgoulianis, K.I.; Basdekis, G. Athletes with mild post-COVID-19 symptoms experience increased respiratory and metabolic demands: A cross-sectional study. Sports Med. Health Sci. 2023, 5, 106–111. https://doi.org/10.1016/j.smhs.2022.10.004.

11. Stojmenović, T.; Marković, S. Impaired Cardiorespiratory Fitness of Elite Athletes after Asymptomatic or Mild SARS-CoV-2 Infection. Medicina 2024, 60, 786. https://doi.org/10.3390/medicina60050786.

12. Śliż, D.; Wiecha, S.; Ulaszewska, K.; Gąsior, J.S.; Lewandowski, M.; Kasiak, P.S.; Mamcarz, A. COVID-19 and athletes: Endurance sport and activity resilience study—CAESAR study. Front. Physiol. 2022, 13, 1078763. https://doi.org/10.3389/fphys.2022.1078763.
